# Supplementary material for: Repetitive marsquakes in Martian upper mantle
Source: Nat Commun. 2022 Mar 30;13:1695. doi: 10.1038/s41467-022-29329-x (PMC8967838; doi:10.1038/s41467-022-29329-x)
Supplement: Supplementary file 1 — Supplementary Information [file 41467_2022_29329_MOESM1_ESM.pdf]

Supplementary Materials for

Repetitive marsquakes in Martian upper mantle

Weijia Sun, Hrvoje Tkalčić

Correspondence to: hrvoje.tkalcic@anu.edu.au

This file includes:

Supplementary Notes 1 to 4

Supplementary Figures 1 to 84

Supplementary Tables 1 to 4

Supplementary Notes

Here we describe the data pre-processing steps for the matched filter (MF) method and show all successful detections of marsquakes in **Supplementary Note 1**. The synthetic experiments that guided the selection of the MF method parameters are given in **Supplementary Note 2**. We further investigate cross-correlation coefficients, signal-to-noise ratios, detected vs. template events amplitude ratios, and their relationship with the local mean solar time in **Supplementary Note 3**. Finally, **Supplementary Note 4** shows an analysis supporting our interpretation of the repetitive marsquakes.

Supplementary Note 1: Data processing for MF method and successful detections of marsquakes

1.1 Data processing workflow

In this section, we summarize the workflow of the MF method in more detail than in the main text. The waveform data pre-processing consists of the following steps:

1) Identify the timing of well-documented Martian events. The nine marsquakes used in this work are listed in Supplementary Table 1. The S0173a and S0235b events are assigned quality A and have the smallest S-wave-arrival uncertainties of 2 s.

2) Divide the continuous three-component Martian data into 24-hour segments.

3) Filter the continuous data using a two-way Butterworth bandpass filter of 0.1-0.8 Hz.

4) Trim the template waveforms in the time window between -2 s and 20 s relative to the S-wave arrivals listed in Supplementary Table 1.

5) Match the three-component template waveforms with continuous waveforms via normalized cross-correlation (Equation (3) in Materials and Methods). De-glitching and synthetic experiments considering glitches were performed to avoid possible misinterpretation (Supplementary Note 2).

6) Compute the median absolute deviation (MAD) by summing the three-component cross-correlation coefficients for each data segment, according to Equation (2) in Materials and Methods.

7) Set the detection threshold. We determine (Supplementary Note 2) that the 7 times of MAD threshold is the most optimal for detecting small events. All the potential detections are verified through visual inspections to avoid mis-detections.

8) Compute amplitude ratio relative to the template event according to Equation (5) from Materials and Methods.

The MF method can be applied to non-standard U, V, and W components or the rotated Z, N, and E components. This is because the template and continuous Martian field data are processed using precisely the same procedures.

Moreover, the InSight SEIS owns three very broadband oblique sensors with a tilt of ~30° relative to the horizon [1]. The incident angles of the P and S phases of the three located marsquakes are between 25.7° and 27.3° as shown in Supplementary Table 2. The non-standard UVW components with a dip angle of ~30°, approximately equivalent to the incident angle, could lead to more weights to tilt incident wavefield arrivals, which are weak. In addition, the horizontal (NE) components have higher coherencies with pressure

perturbations than the vertical Z components[2]. With these considerations, we apply the MF method to the UVW components.

Supplementary Table 1. Template marsquakes used in this work. The quality, the times of P- and S-wave arrivals, and the moment magnitudes were taken from Giardini, et al. [3]. The uncertainties of first arrivals are listed.

Event name	Event quality	P arrival time (uncertainty)	S arrival time (uncertainty)	Magnitude Mw	Number of new detections by MF
S0105a	B	2019-03-14T21:03:31 (±20)	2019-03-14T21:06:39 (±20)	3.2	0
S0133a	B	2019-04-12T18:14:35 (±60)	2019-04-12T18:17:56 (±20)	3.2	0
S0154a	B	2019-05-04T07:07:05 (±20)	2019-05-04T07:11:57 (±20)	3.5	0
S0173a	A	2019-05-23T02:22:59.1 (±1)	2019-05-23T02:25:53.8 (±2)	3.6	11
S0183a	B*	2019-06-03T02:27:45.8 (±1)	2019-06-03T02:32:09 (±10)	3.1	1
S0185a	B	2019-06-05T02:13:51 (±20)	2019-06-05T02:19:35 (±20)	3.1	0
S0189a	B	2019-06-09T05:40:06 (±20)	2019-06-09T05:43:20 (±20)	3.0	0
S0235b	A	2019-07-26T12:19:19.3 (±2)	2019-07-26T12:21:56.1 (±2)	3.6	34
S0325a	B	2019-10-26T06:58:58.9 (±1)	2019-10-26T07:02:56 (±10)	3.7	1

* The event quality of S0183a is corrected to B as in Mars Seismic Catalogue, Version 6 (doi:10.12686/a11).

Supplementary Table 2. Event information for the three located marsquakes assuming the focal depth of 40 km. The 1-D Martian velocity model used here can be retrieved from IPGP (doi:10.18715/IPGP.2021.kpmqrnz8).

Event name	Latitude (°)	Longitude (°)	Azimuth (°)	Back azimuth (°)	Distance (km)	Distance (°)	Incident angle of P arrival (°)	Incident angle of S arrival (°)
S0173a	3.4372	164.9642	273.1	91.0	1732.6	29.3	27.2	28.4
S0183a	15.2916	-179.0627	261.3	73.1	2709.8	45.8	25.7	27.8
S0235b	11.4070	162.8890	257.9	74.1	1647.0	27.8	27.3	28.5

1.2 Successful detections of marsquakes

We here identify 47 newly detected events occurring during the day and night. Dahmen, et al. [4] reported a thermally-controlled class of super-high frequency (SF) events, which is also repetitive. Notably, the SF events are characterized by substantially different behaviors than the LF events. The SF events are periodically repeated at a similar time of the day due to temperature variations; however, their waveforms are dominated by frequencies in the frequency band of ~5-30 Hz, thus lacking low-frequency components [4]. In contrast, our detections are dominated by frequencies in the frequency band of 0.1-0.8 Hz and are randomly distributed in time, as shown in Fig. 3. In conclusion, our newly detected events are not SF events.

This section demonstrates the successful detections of marsquakes using the MF method (Supplementary Figures 1-49). Supplementary Figure 50 illustrates Benford’s Law method applied on the two most prominent marsquakes.

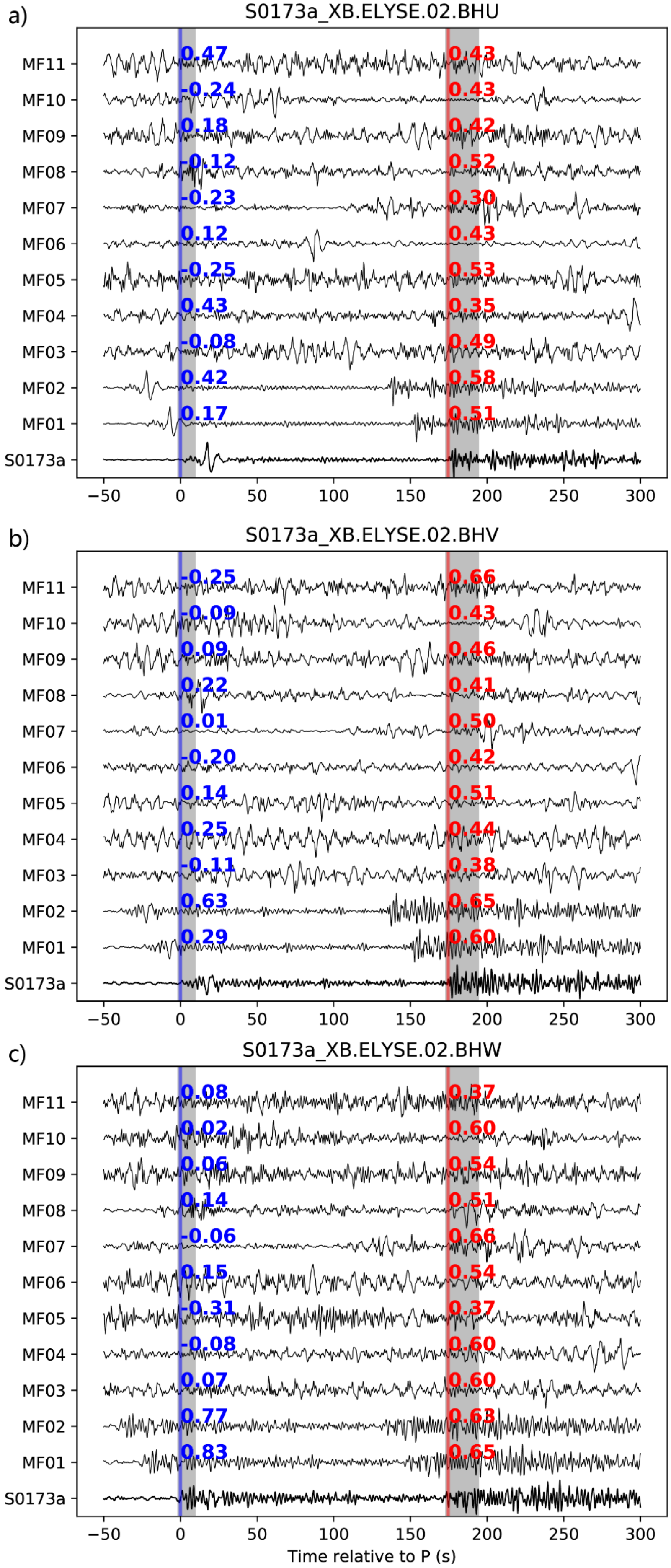
a)
S0173a_XB.ELYSE.02.BHU
MF11
MF10
MF09
MF08
MF07
MF06
MF05
MF04
MF03
MF02
MF01
S0173a
0.47
-0.24
0.18
-0.12
-0.23
0.12
-0.25
0.43
-0.08
0.42
0.17
0.43
0.43
0.42
0.52
0.30
0.43
0.53
0.35
0.49
0.58
0.51
−50
0
50
100
150
200
250
300
b)
S0173a_XB.ELYSE.02.BHV
-0.25
-0.09
0.09
0.22
0.01
-0.20
0.14
0.25
-0.11
0.63
0.29
0.66
0.43
0.46
0.41
0.50
0.42
0.51
0.44
0.38
0.65
0.60
c)
S0173a_XB.ELYSE.02.BHW
0.08
0.02
0.06
0.14
-0.06
0.15
-0.31
-0.08
0.07
0.77
0.83
0.37
0.60
0.54
0.51
0.66
0.54
0.37
0.60
0.60
0.63
0.65
Time relative to P (s)

Supplementary Figure 1. The correlation coefficients of the P- and S-wave arrivals of the newly detected events using the MF technique for S0173a. A) The BHU components of the Martian events aligned so that the P-wave arrival is at 0 s. The blue and red lines denote the P- and S-wave arrivals. The time windows used in calculating correlation coefficients with the known S0173a event are shown in shaded gray. They range from -2 to 10 s relative to P-wave arrival and from -2 to 20 s relative to S-wave arrival. The coefficients are labeled correspondingly. B) and C) same as A) but for BHV and BHW components. All waveforms are filtered in the bands of 0.1-0.8 Hz.

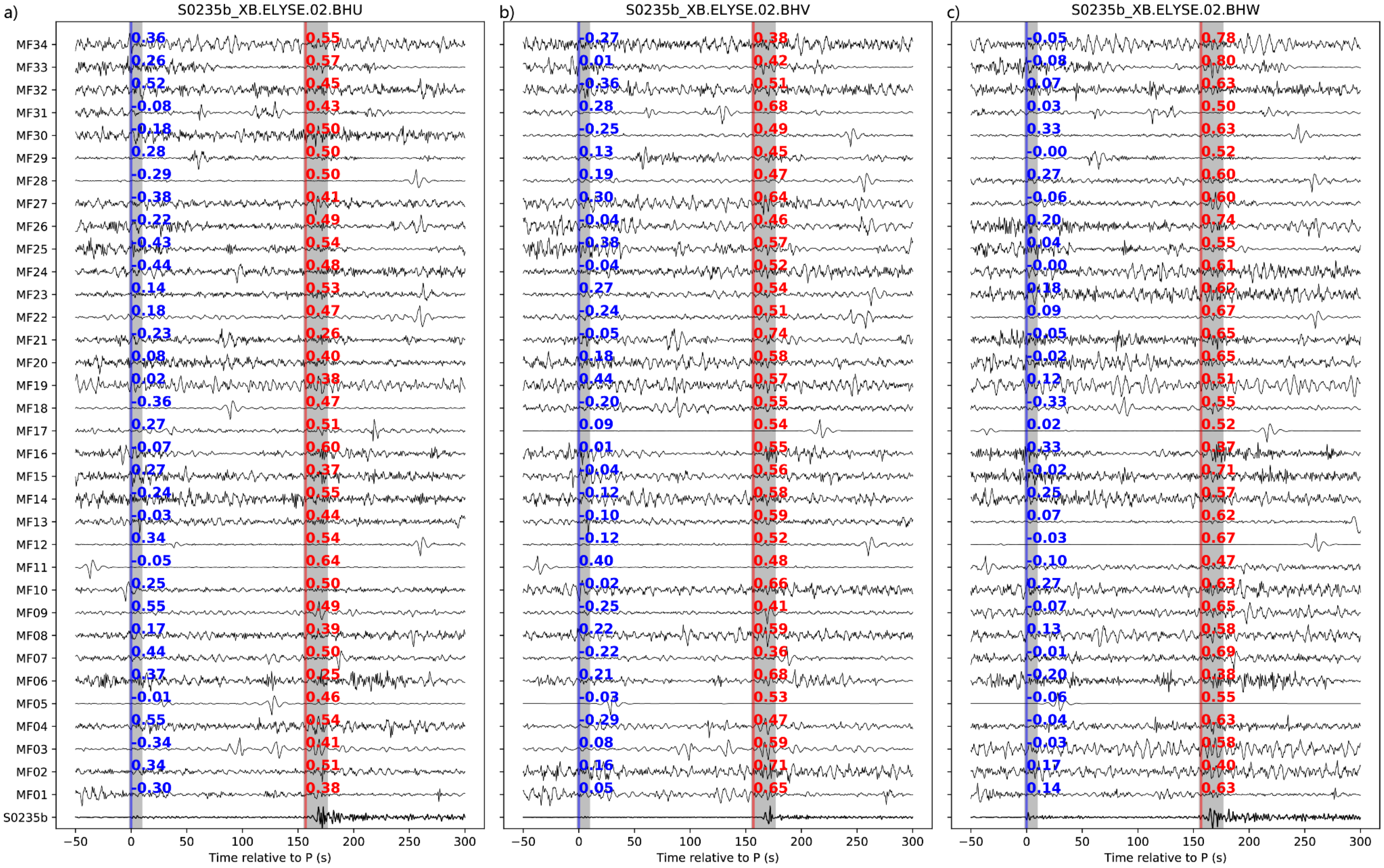
a)
S0235b_XB.ELYSE.02.BHU
b)
S0235b_XB.ELYSE.02.BHV
c)
S0235b_XB.ELYSE.02.BHW
MF34
MF33
MF32
MF31
MF30
MF29
MF28
MF27
MF26
MF25
MF24
MF23
MF22
MF21
MF20
MF19
MF18
MF17
MF16
MF15
MF14
MF13
MF12
MF11
MF10
MF09
MF08
MF07
MF06
MF05
MF04
MF03
MF02
MF01
S0235b
−50 0 50 100 150 200 250 300
Time relative to P (s)

Supplementary Figure 2. Same as Supplementary Figure 1 but for the S0235b event.

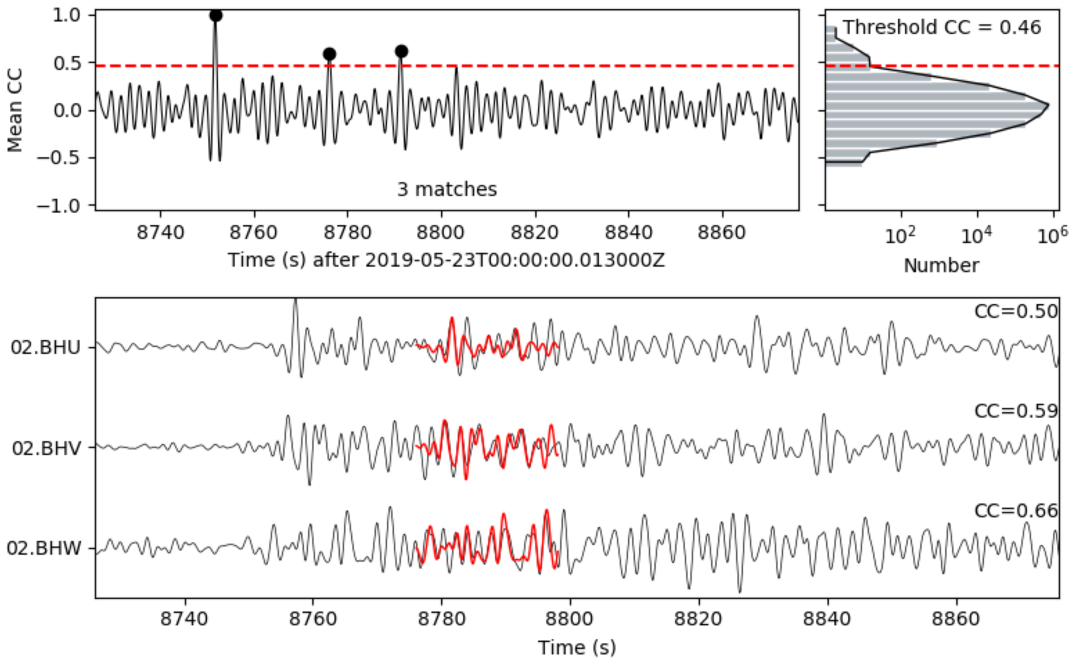

Supplementary Figure 3. The matched-filter detection of S0173a-MF01.

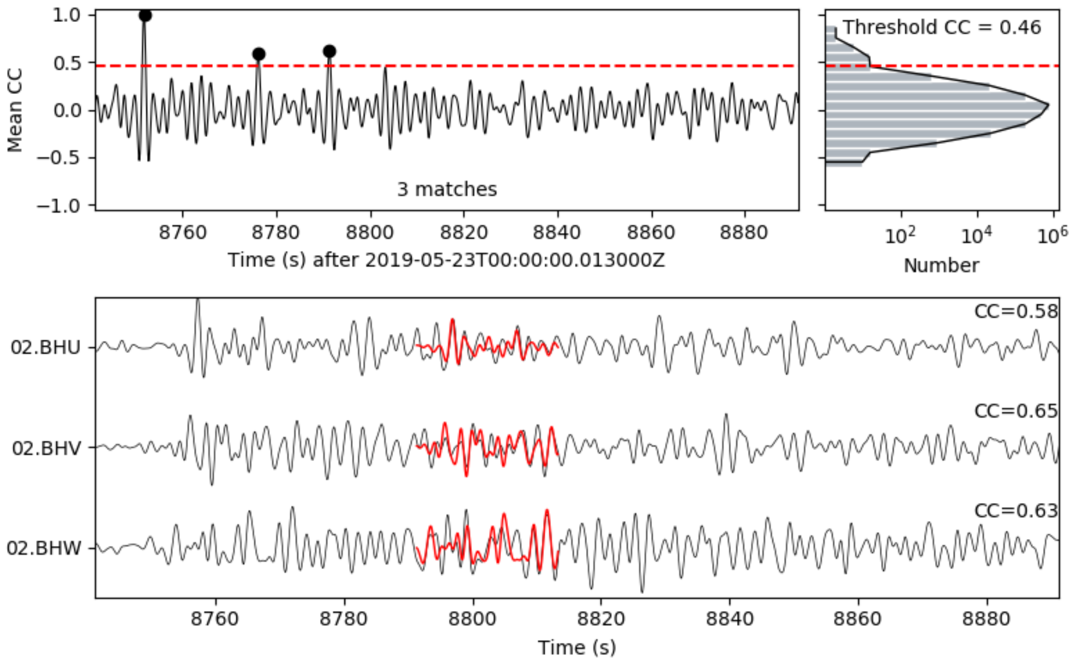

Supplementary Figure 4. The matched-filter detection of S0173a-MF02.

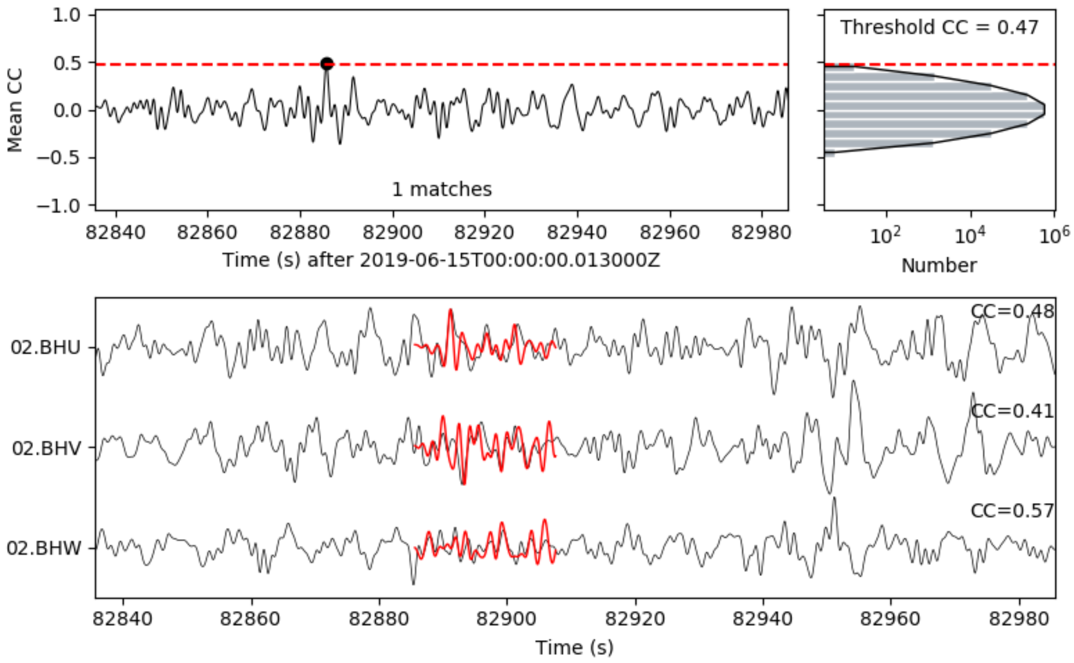

Supplementary Figure 5. The matched-filter detection of S0173a-MF03.

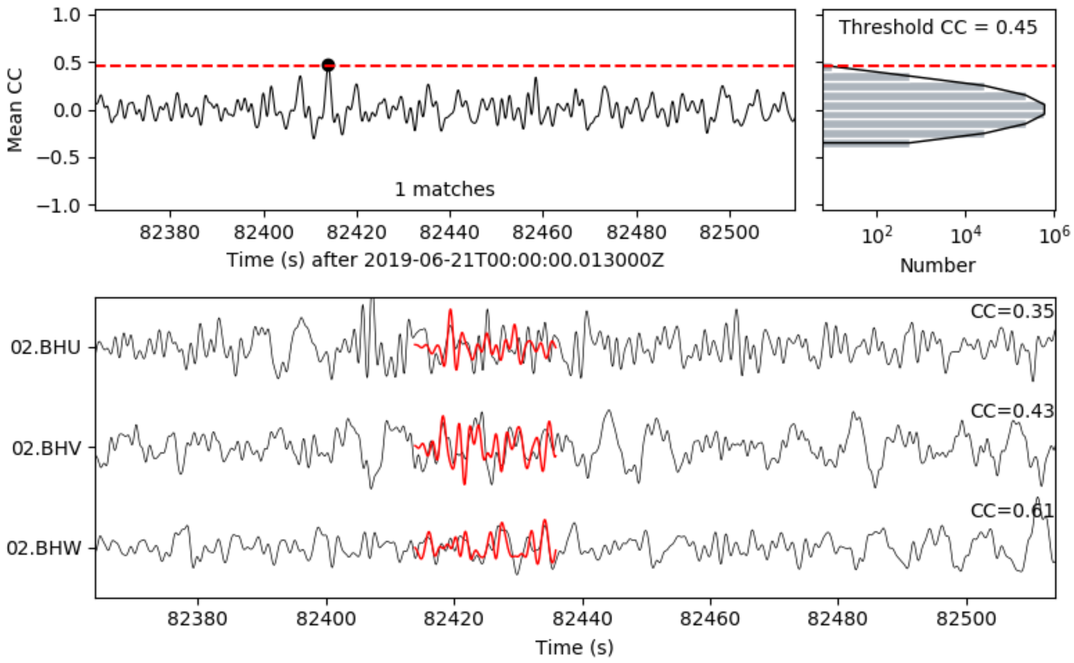

Supplementary Figure 6. The matched-filter detection of S0173a-MF04.

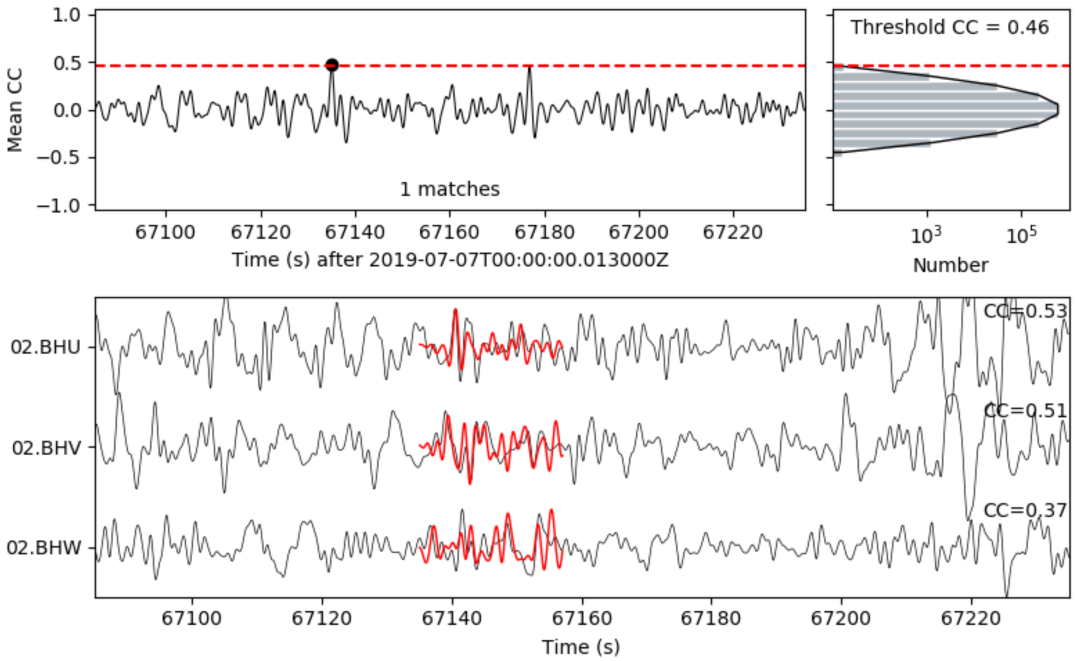

Supplementary Figure 7. The matched-filter detection of S0173a-MF05.

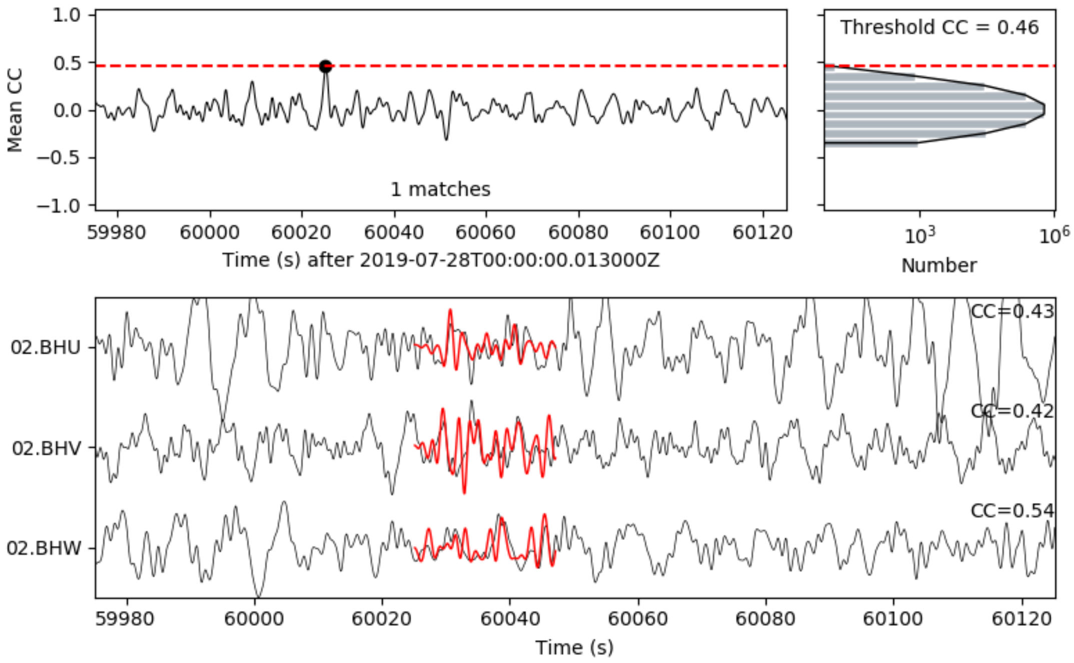

Supplementary Figure 8. The matched-filter detection of S0173a-MF06.

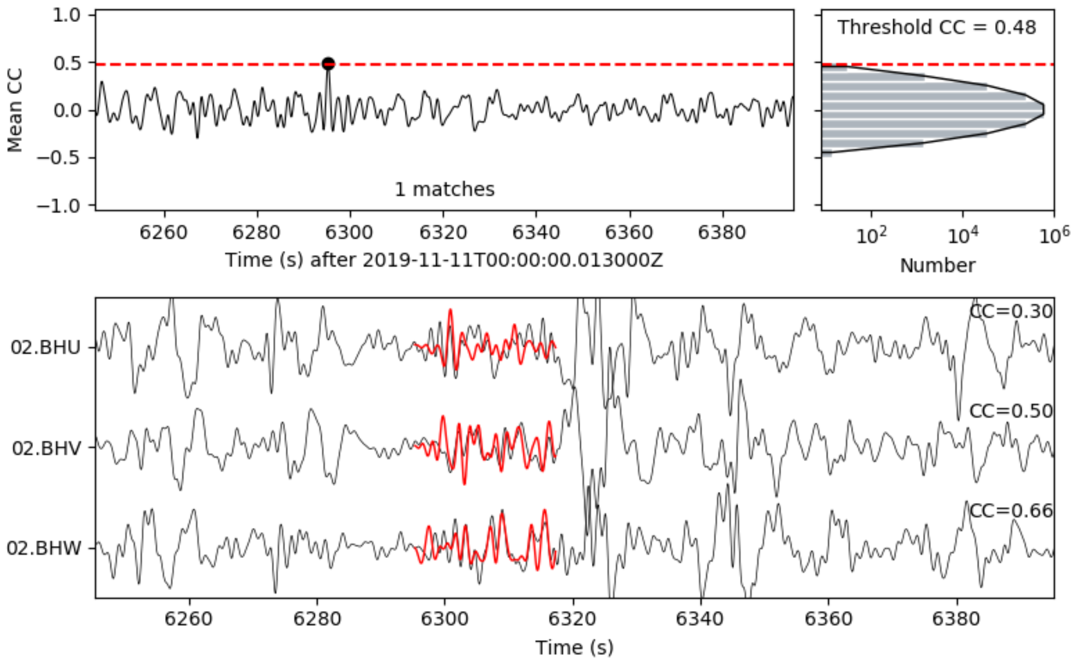

Supplementary Figure 9. The matched-filter detection of S0173a-MF07.

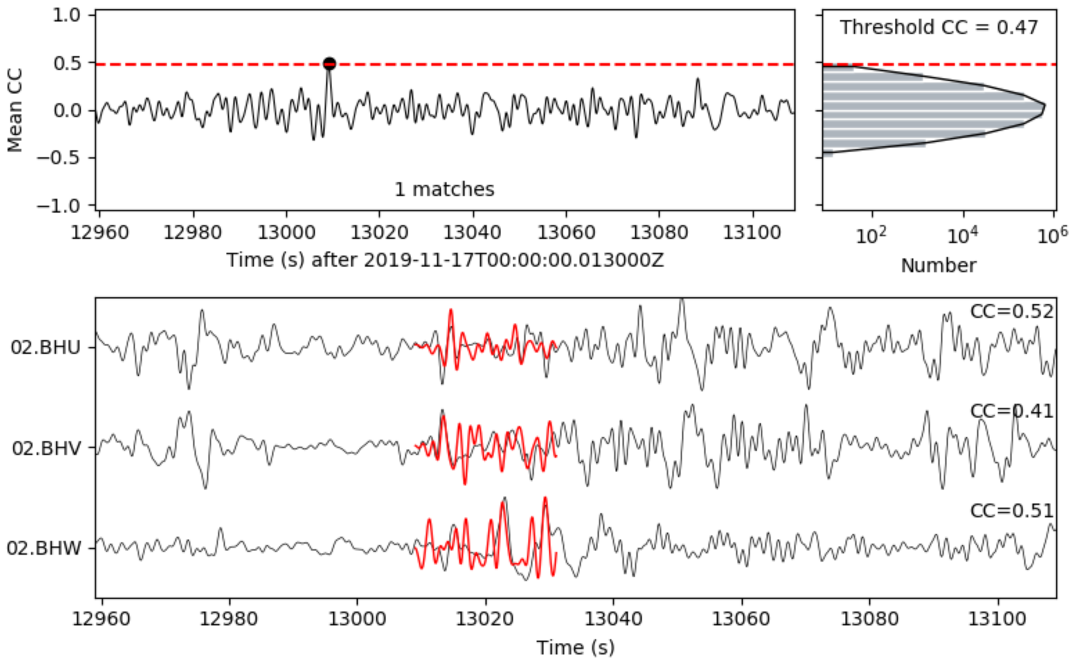

Supplementary Figure 10. The matched-filter detection of S0173a-MF08.

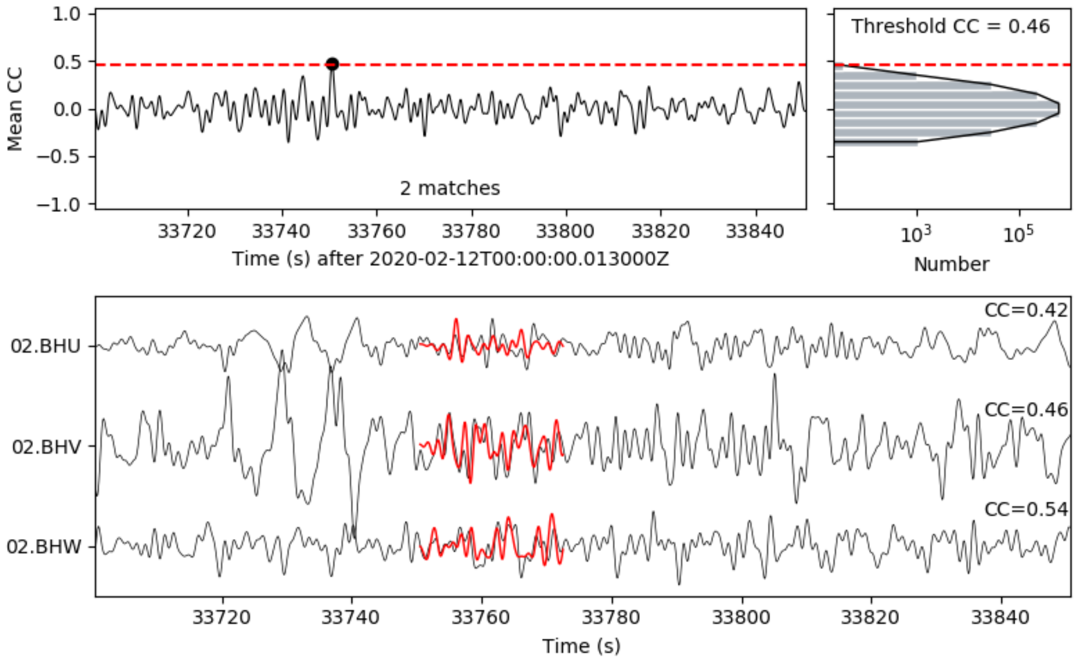

Supplementary Figure 11. The matched-filter detection of S0173a-MF09.

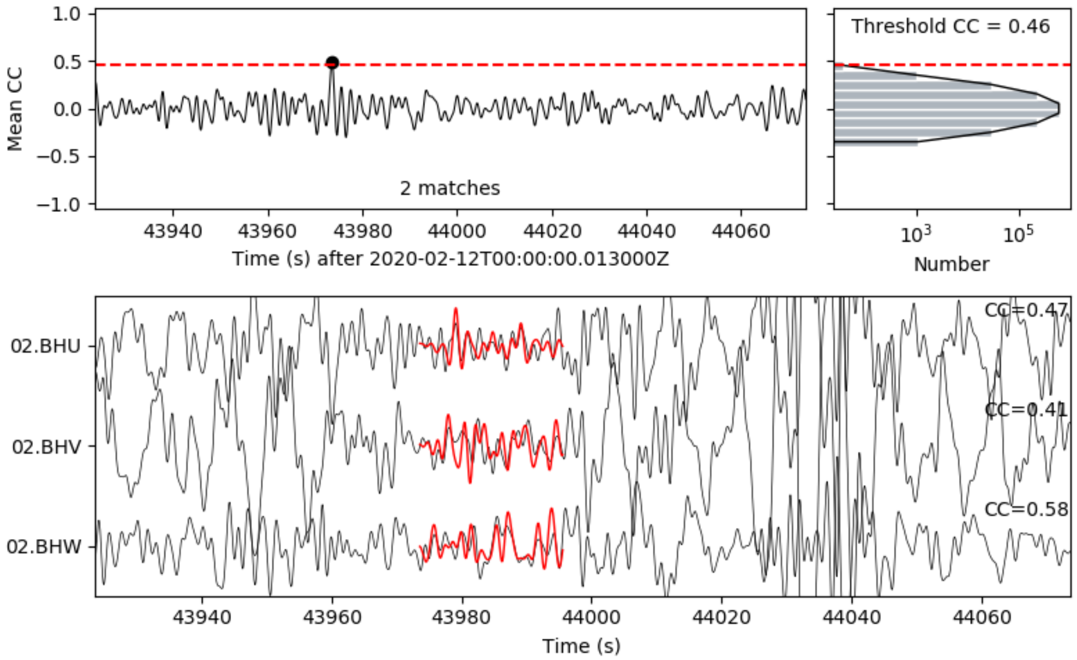

Supplementary Figure 12. The matched-filter detection of S0173a-MF10.

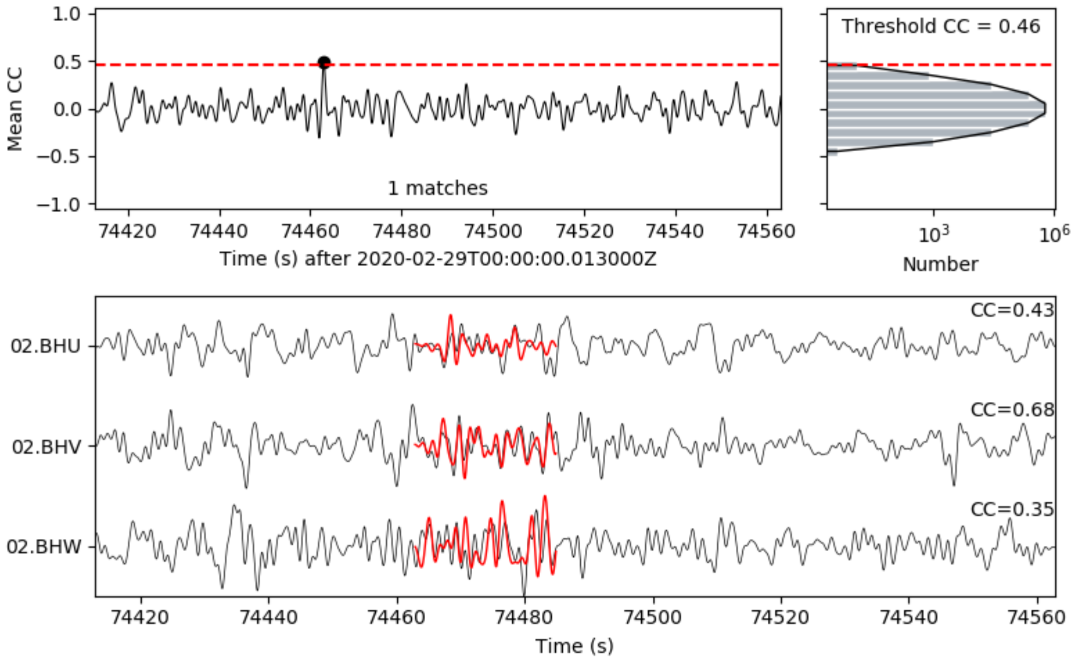

Supplementary Figure 13. The matched-filter detection of S0173a-MF11.

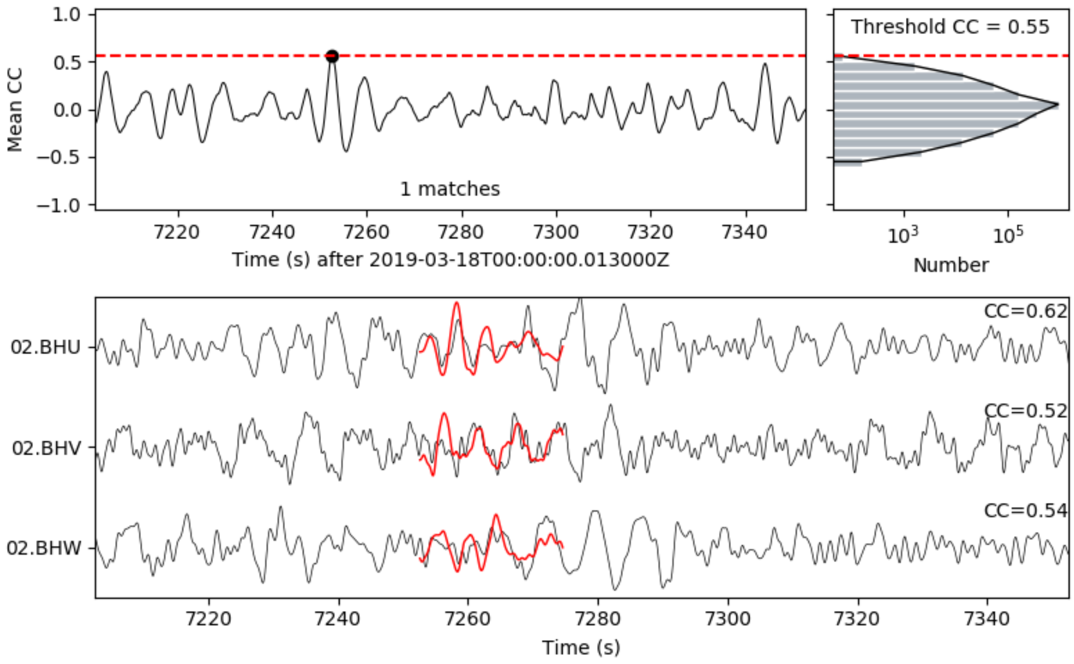

Supplementary Figure 14. The matched-filter detection of S0183a-MF01.

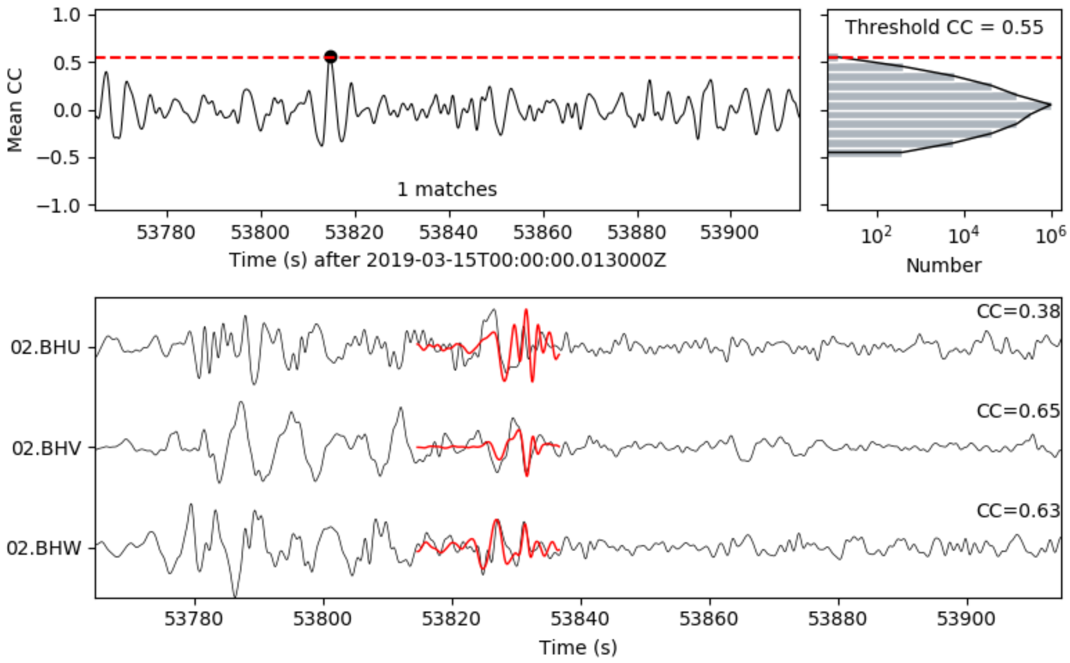

Supplementary Figure 15. The matched-filter detection of S0235b-MF01.

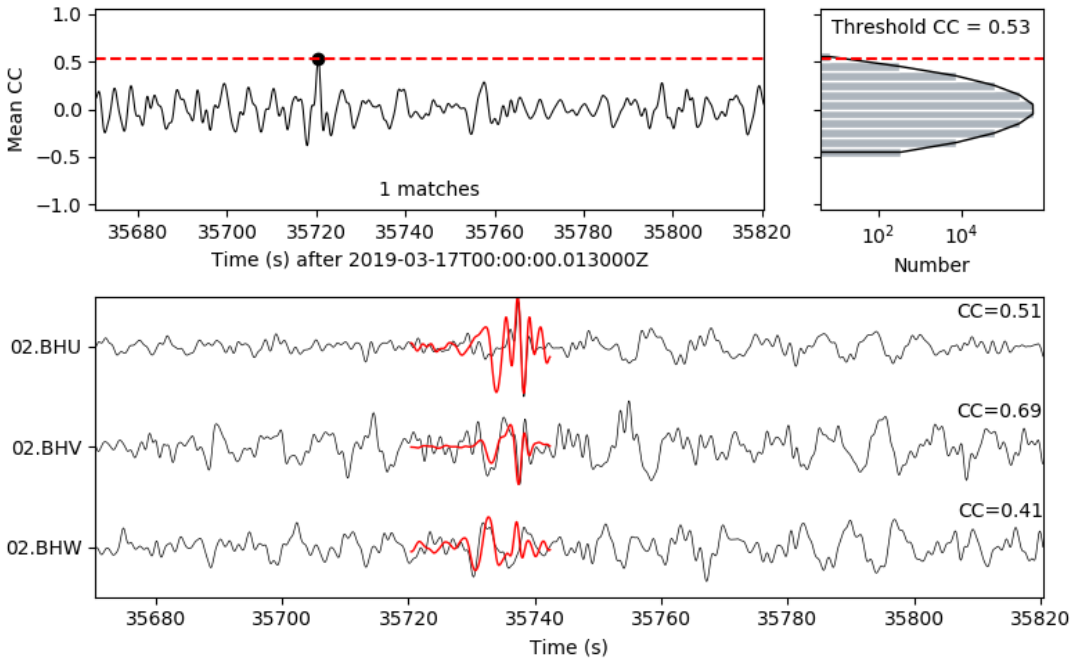

Supplementary Figure 16. The matched-filter detection of S0235b-MF02.

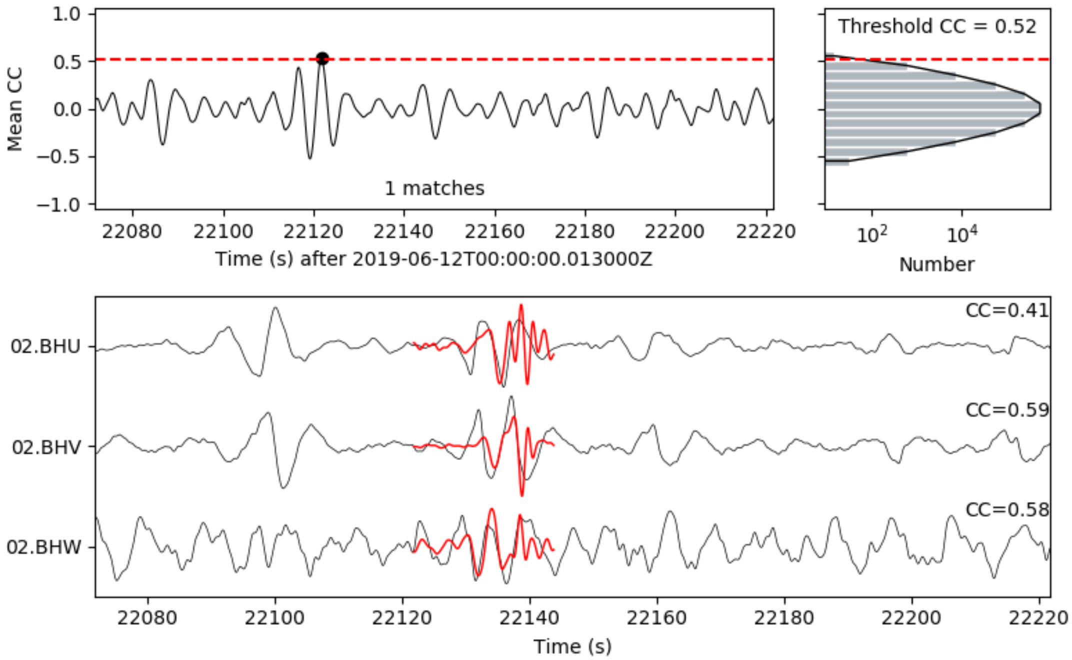

Supplementary Figure 17. The matched-filter detection of S0235b-MF03.

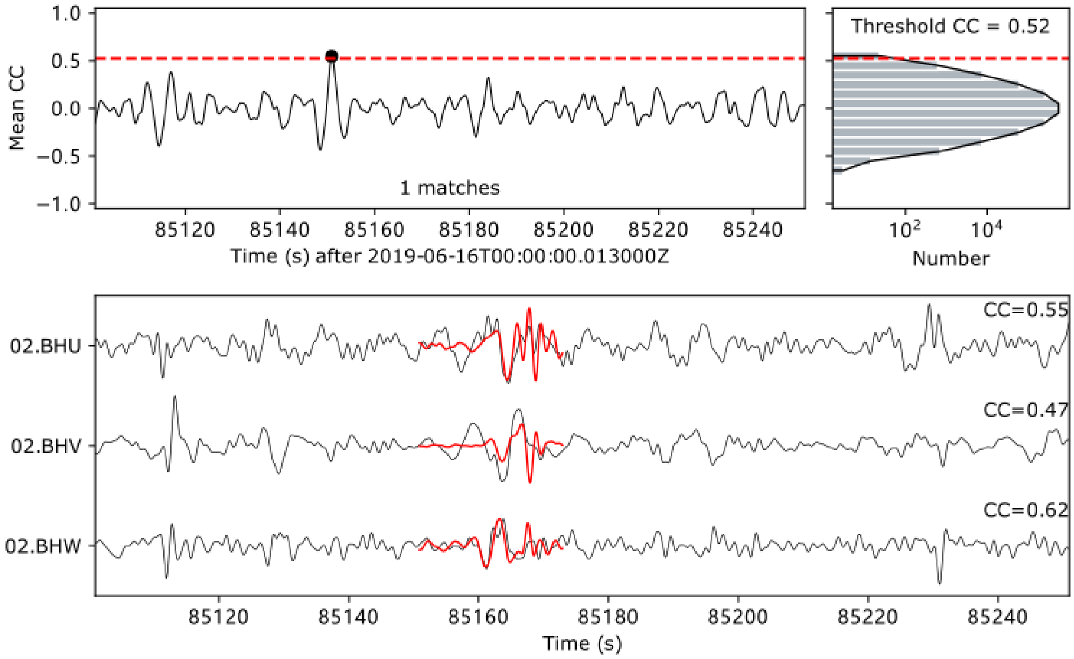

Supplementary Figure 18. The matched-filter detection of S0235b-MF04.

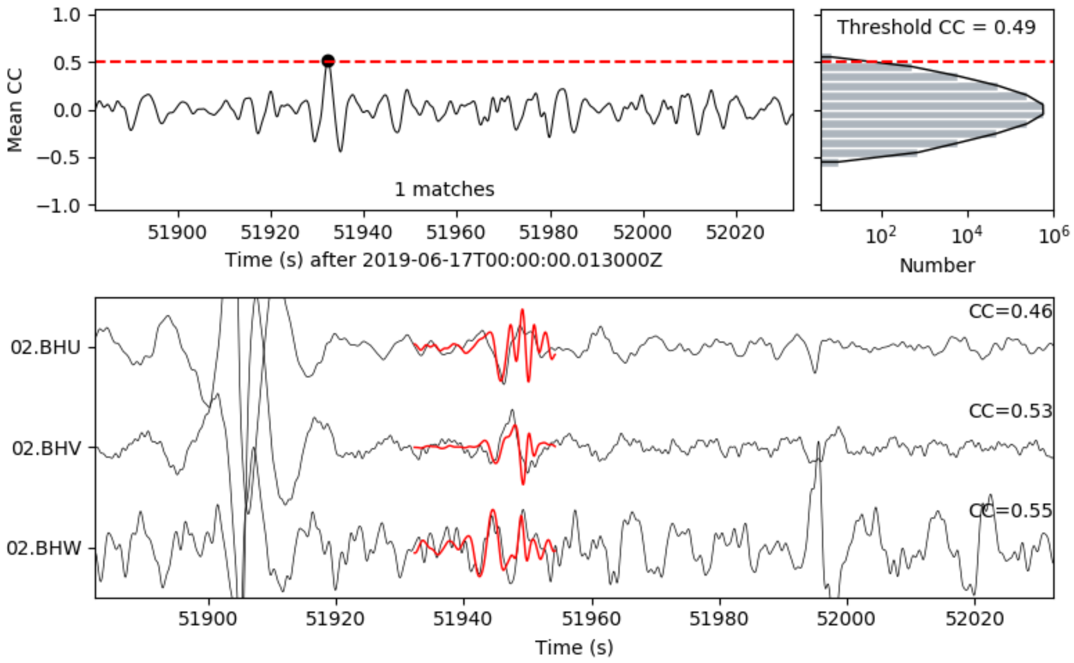

Supplementary Figure 19. The matched-filter detection of S0235b-MF05.

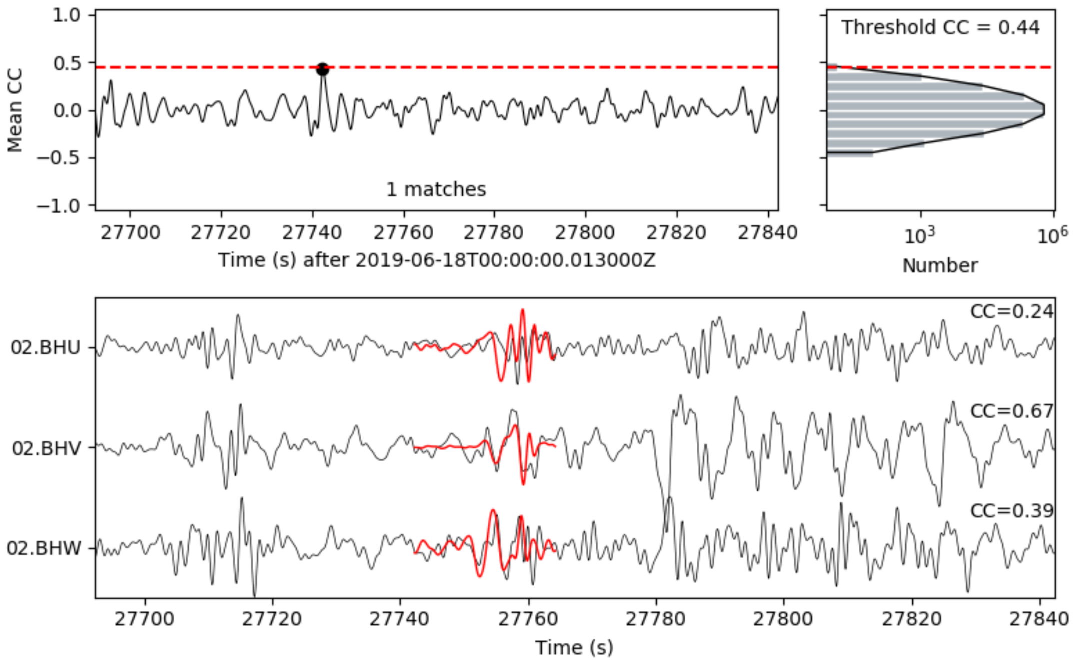

Supplementary Figure 20. The matched-filter detection of S0235b-MF06.

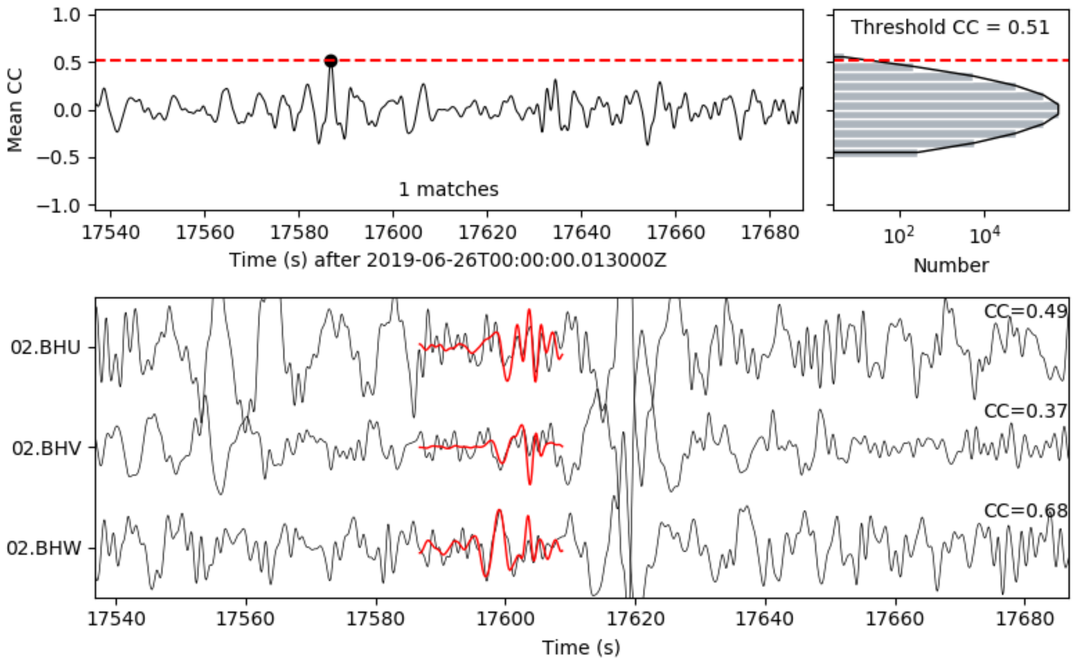

Supplementary Figure 21. The matched-filter detection of S0235b-MF07.

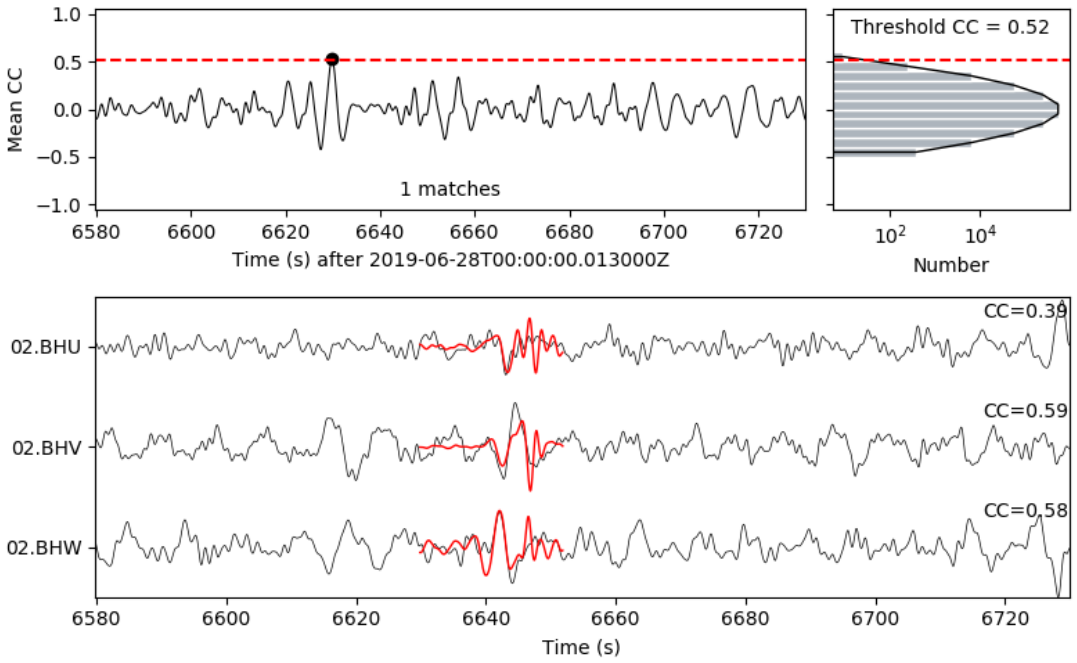

Supplementary Figure 22. The matched-filter detection of S0235b-MF08.

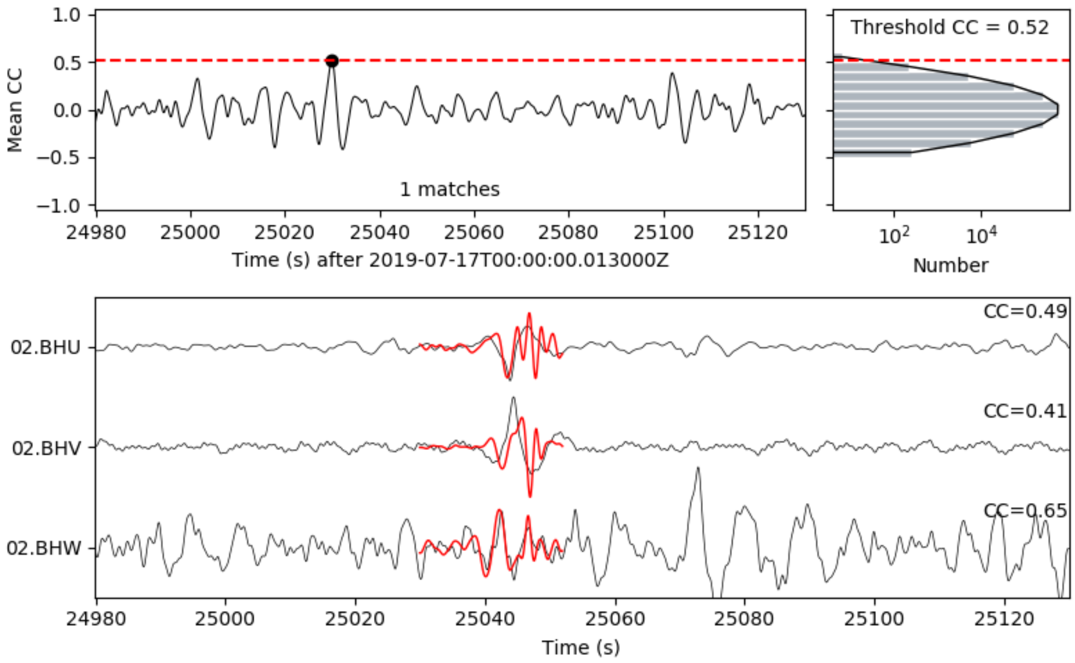

Supplementary Figure 23. The matched-filter detection of S0235b-MF09.

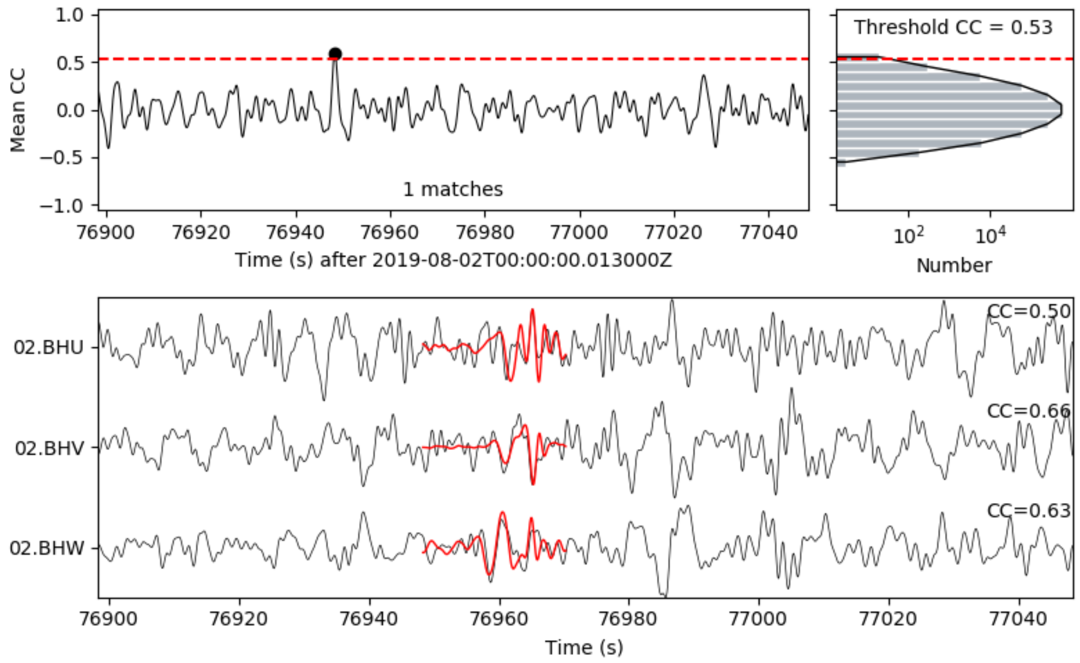

Supplementary Figure 24. The matched-filter detection of S0235b-MF10.

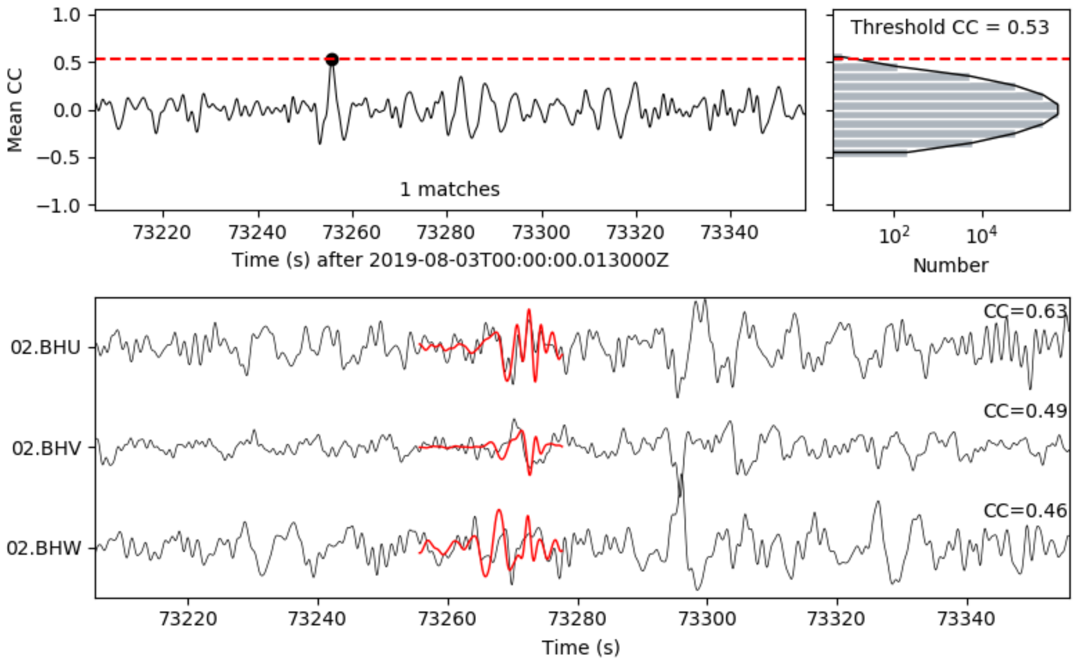

Supplementary Figure 25. The matched-filter detection of S0235b-MF11.

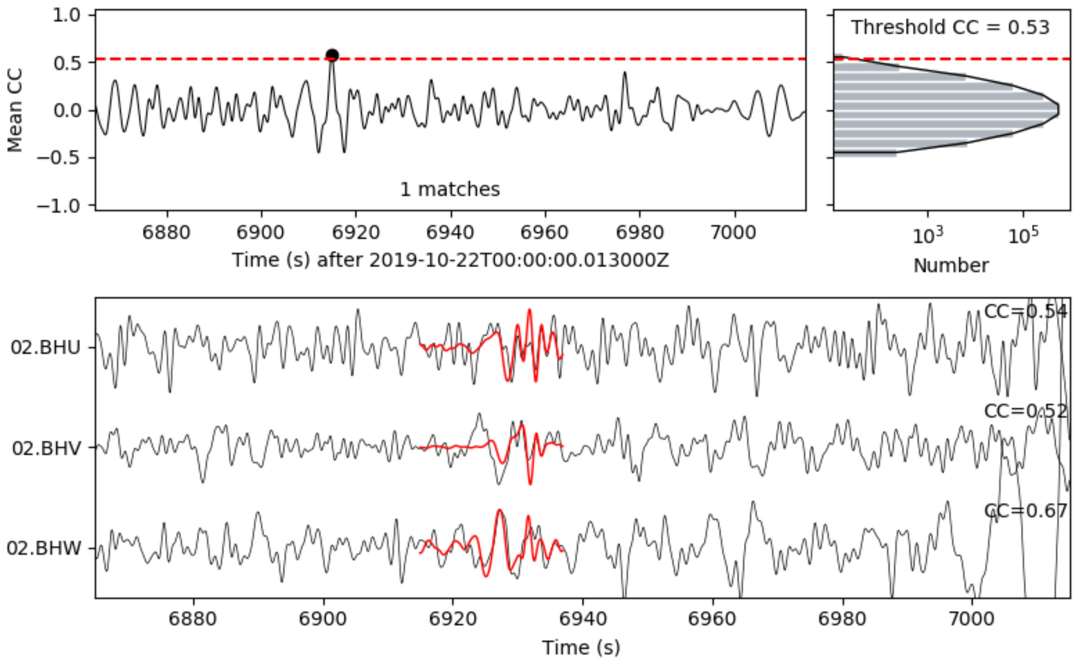

Supplementary Figure 26. The matched-filter detection of S0235b-MF12.

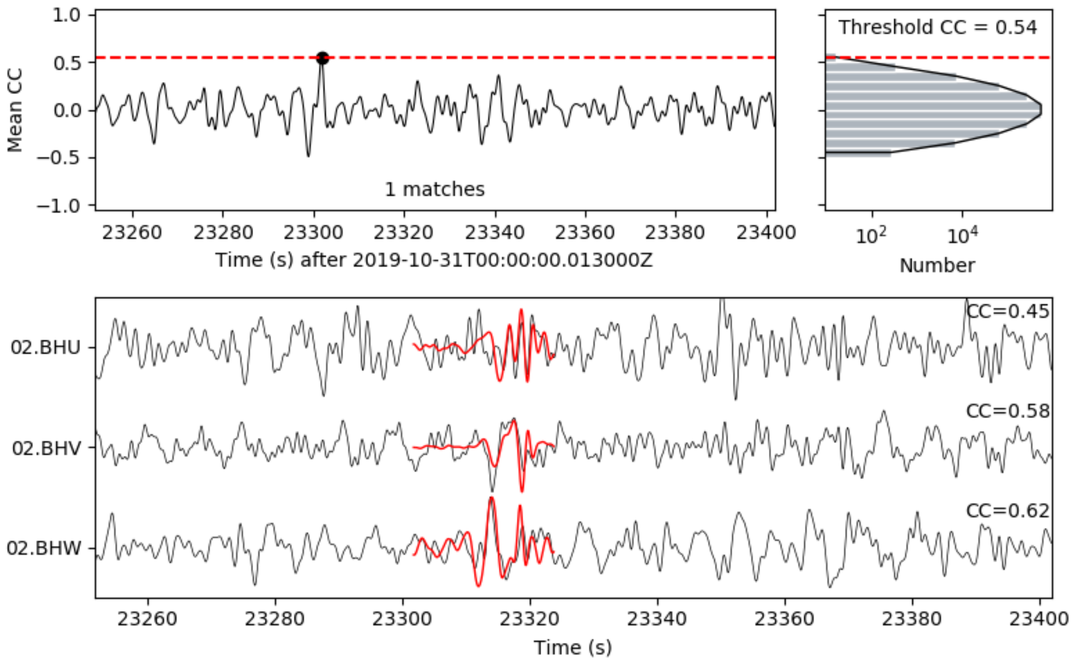

Supplementary Figure 27. The matched-filter detection of S0235b-MF13.

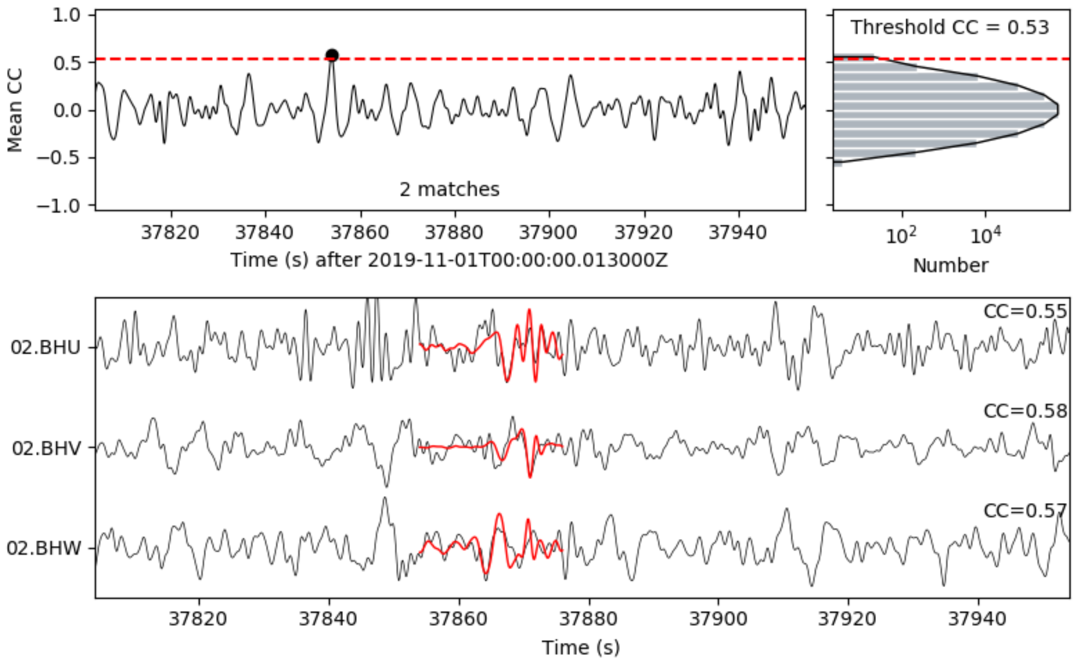

Supplementary Figure 28. The matched-filter detection of S0235b-MF14.

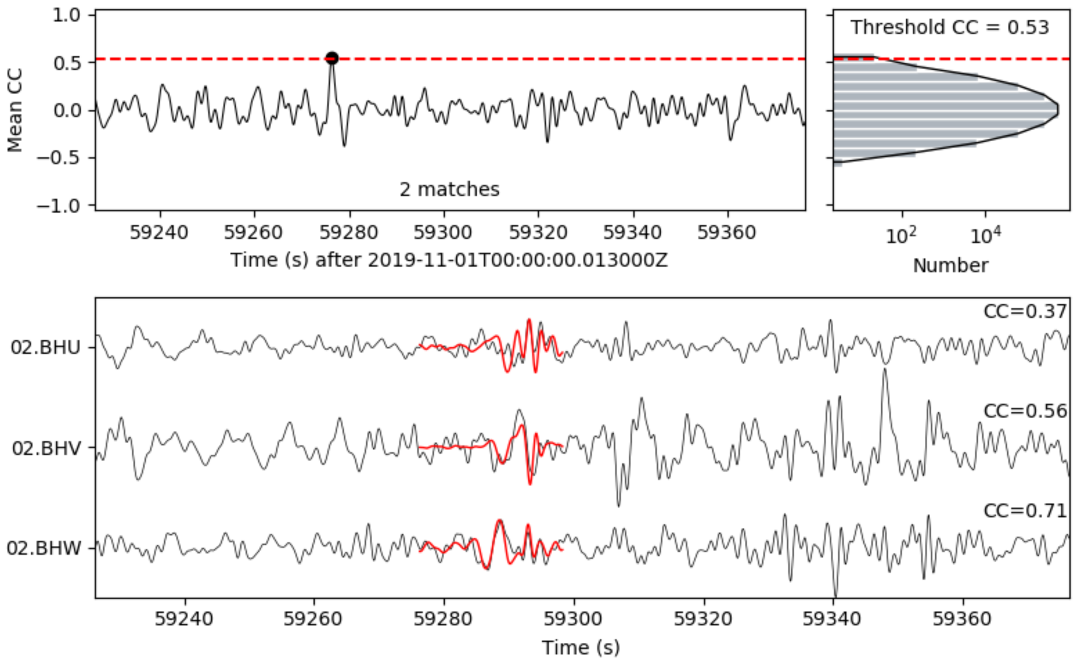

Supplementary Figure 29. The matched-filter detection of S0235b-MF15.

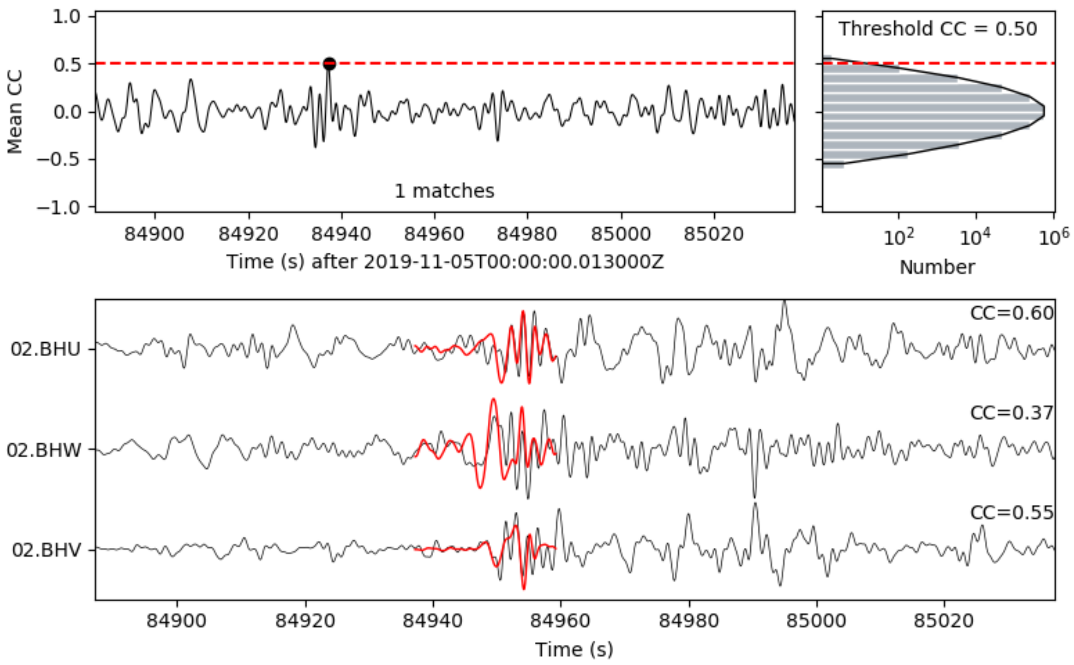

Supplementary Figure 30. The matched-filter detection of S0235b-MF16.

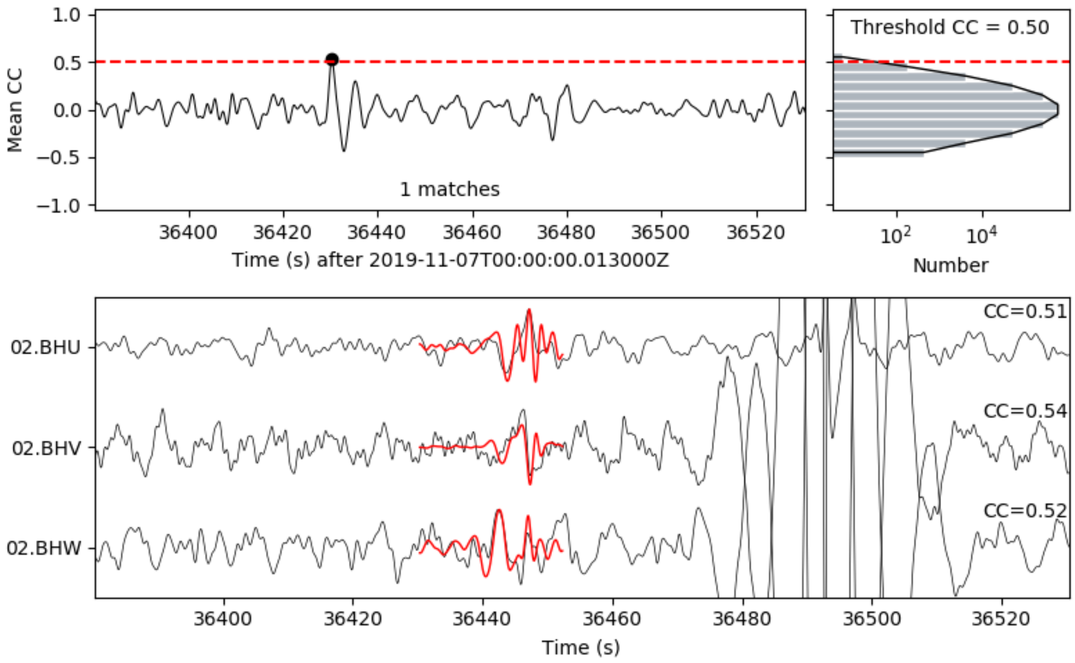

Supplementary Figure 31. The matched-filter detection of S0235b-MF17.

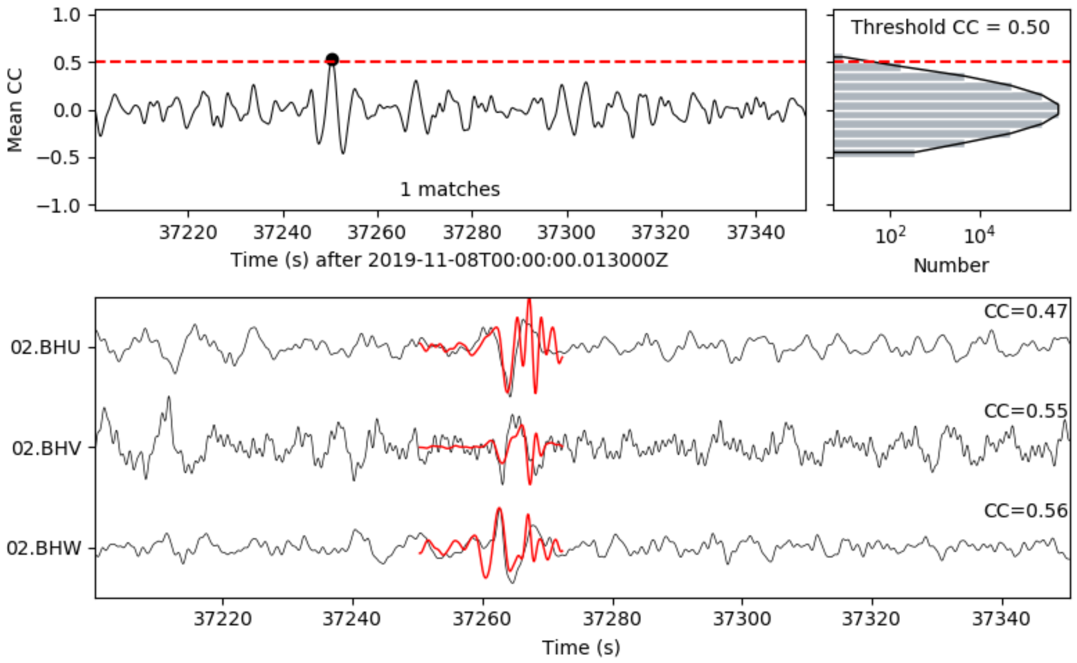

Supplementary Figure 32. The matched-filter detection of S0235b-MF18.

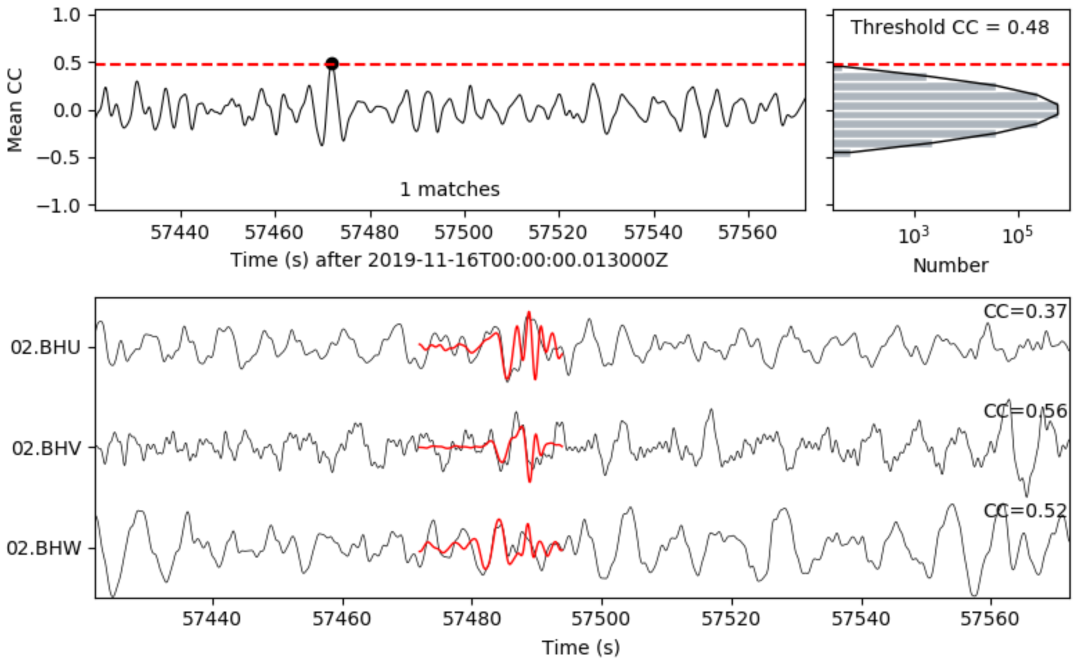

Supplementary Figure 33. The matched-filter detection of S0235b-MF19.

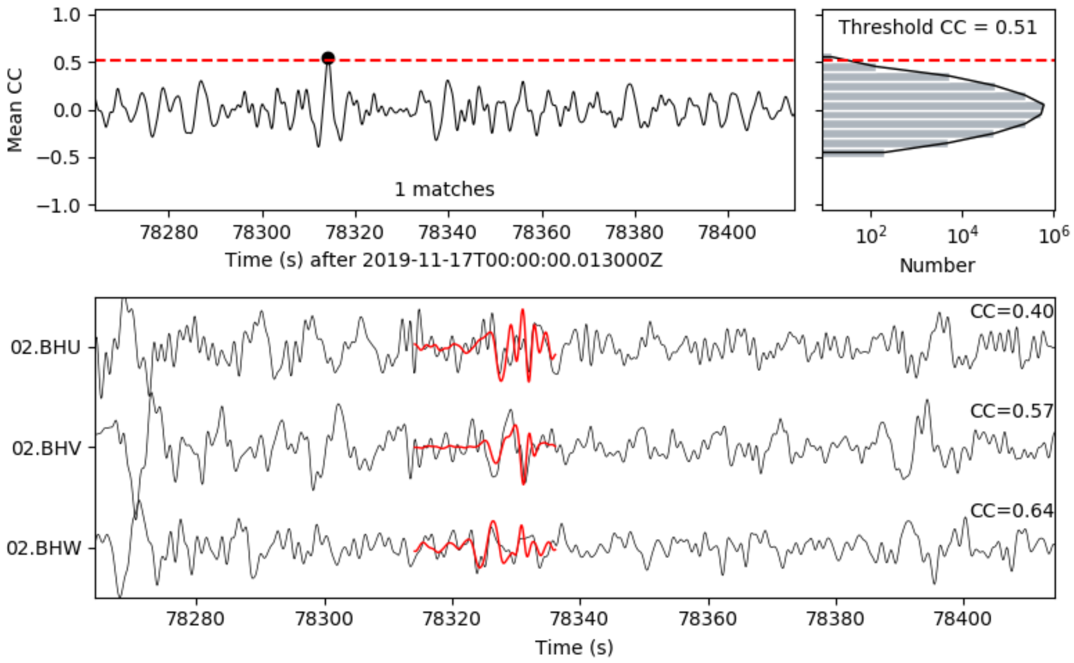

Supplementary Figure 34. The matched-filter detection of S0235b-MF20.

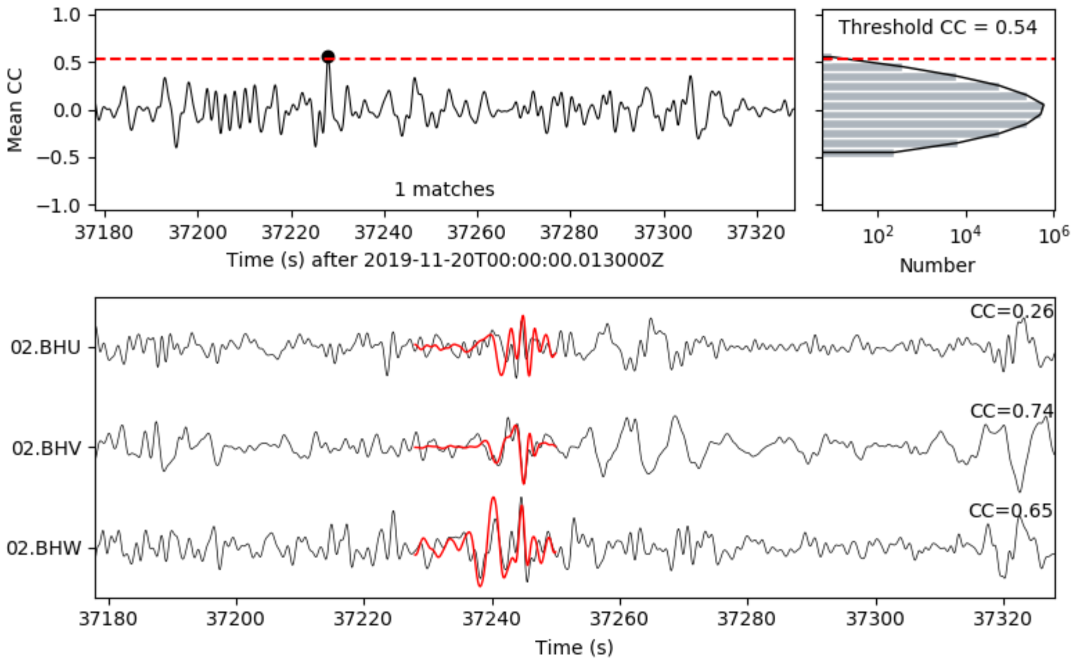

Supplementary Figure 35. The matched-filter detection of S0235b-MF21.

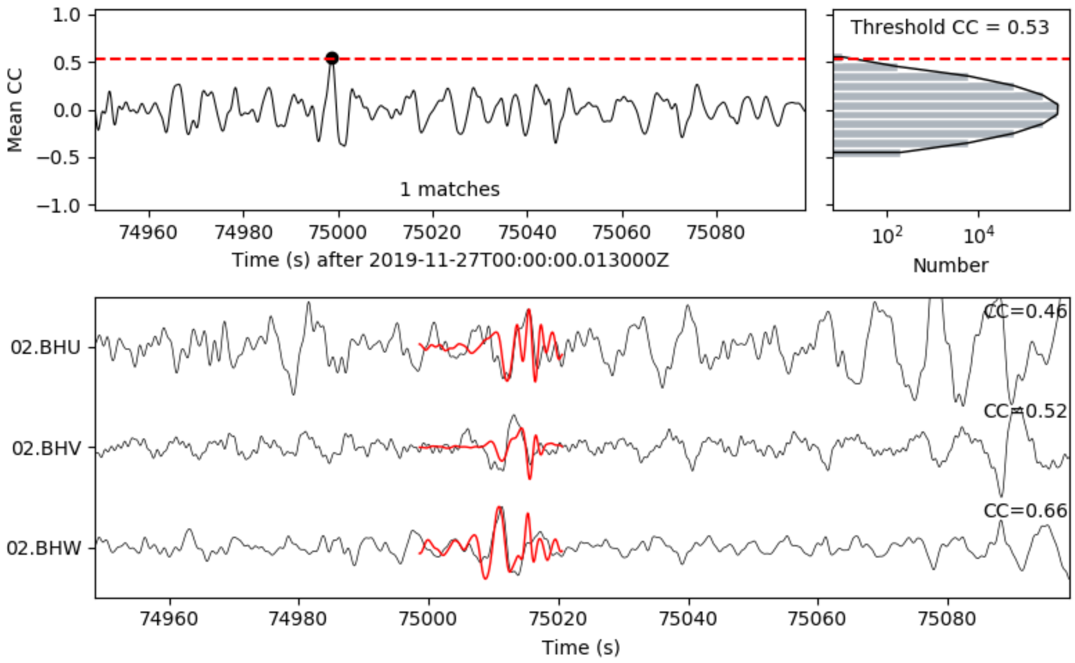

Supplementary Figure 36. The matched-filter detection of S0235b-MF22.

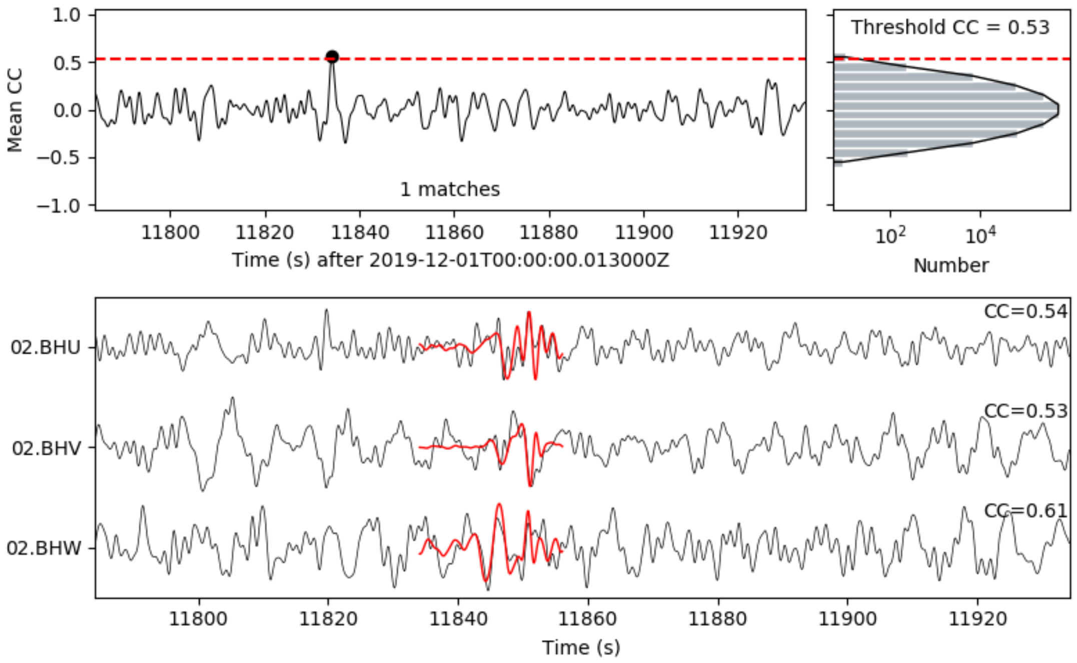

Supplementary Figure 37. The matched-filter detection of S0235b-MF23.

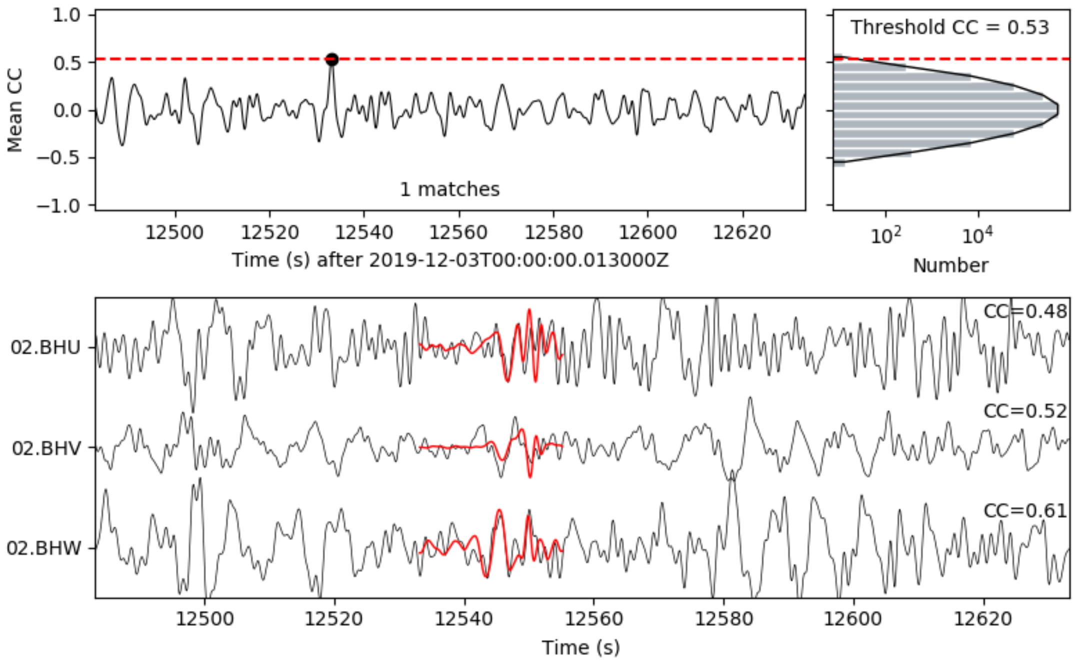

Supplementary Figure 38. The matched-filter detection of S0235b-MF24.

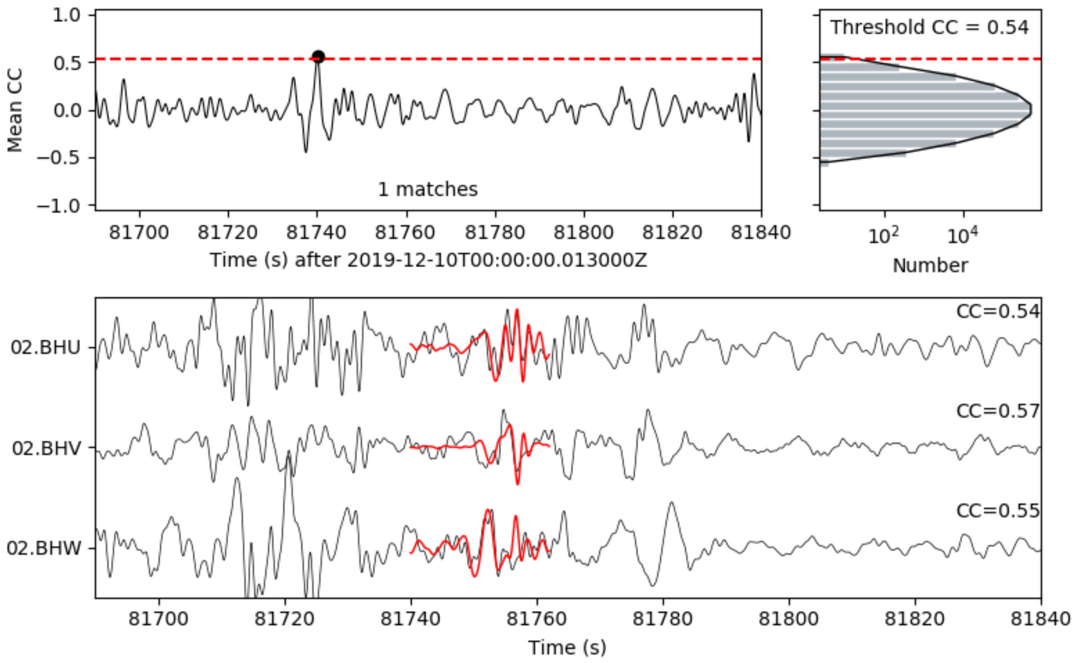

Supplementary Figure 39. The matched-filter detection of S0235b-MF25.

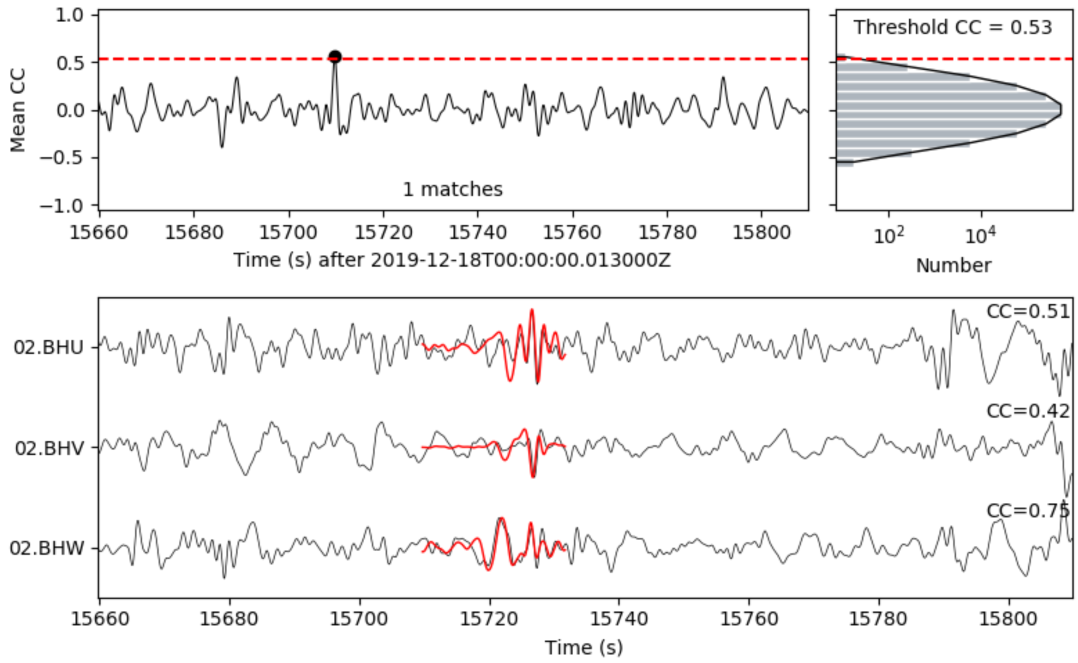

Supplementary Figure 40. The matched-filter detection of S0235b-MF26.

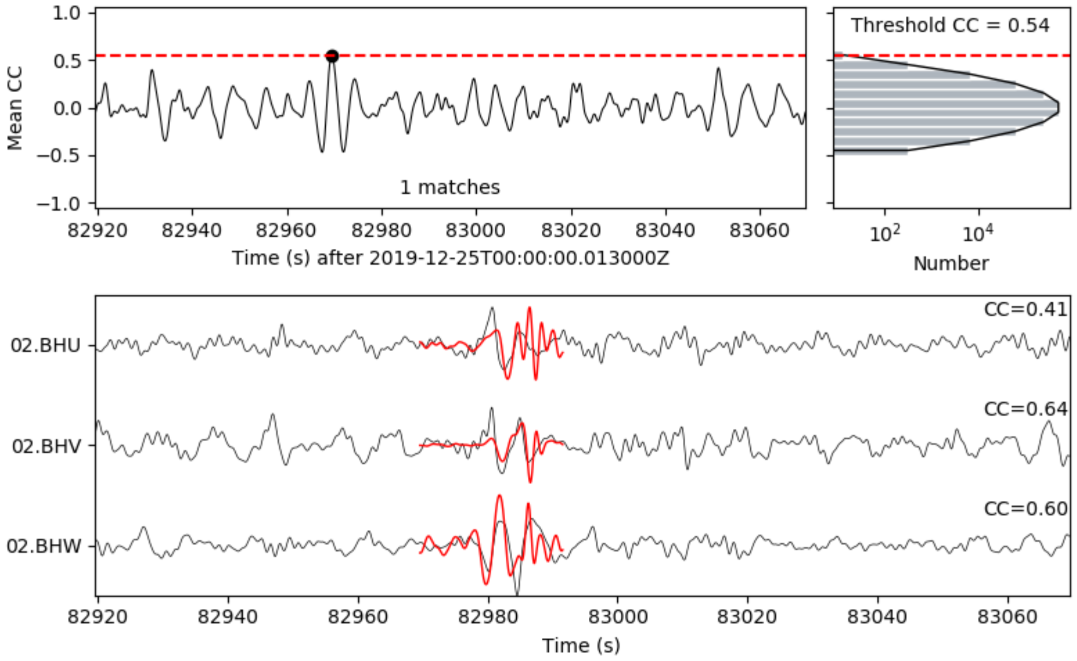

Supplementary Figure 41. The matched-filter detection of S0235b-MF27.

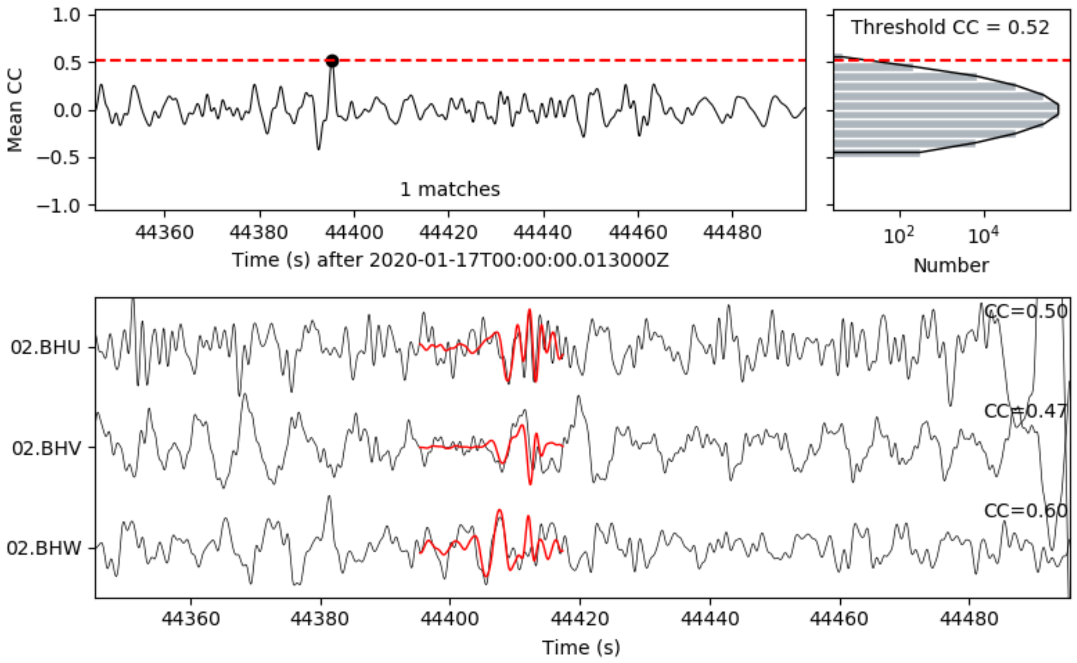

Supplementary Figure 42. The matched-filter detection of S0235b-MF28.

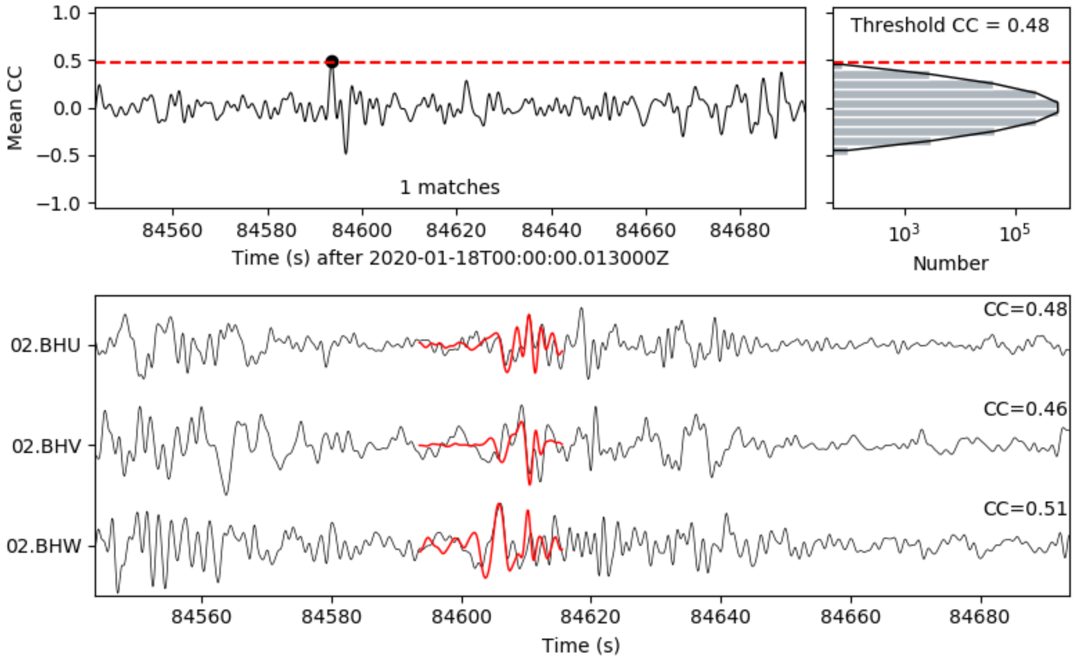

Supplementary Figure 43. The matched-filter detection of S0235b-MF29.

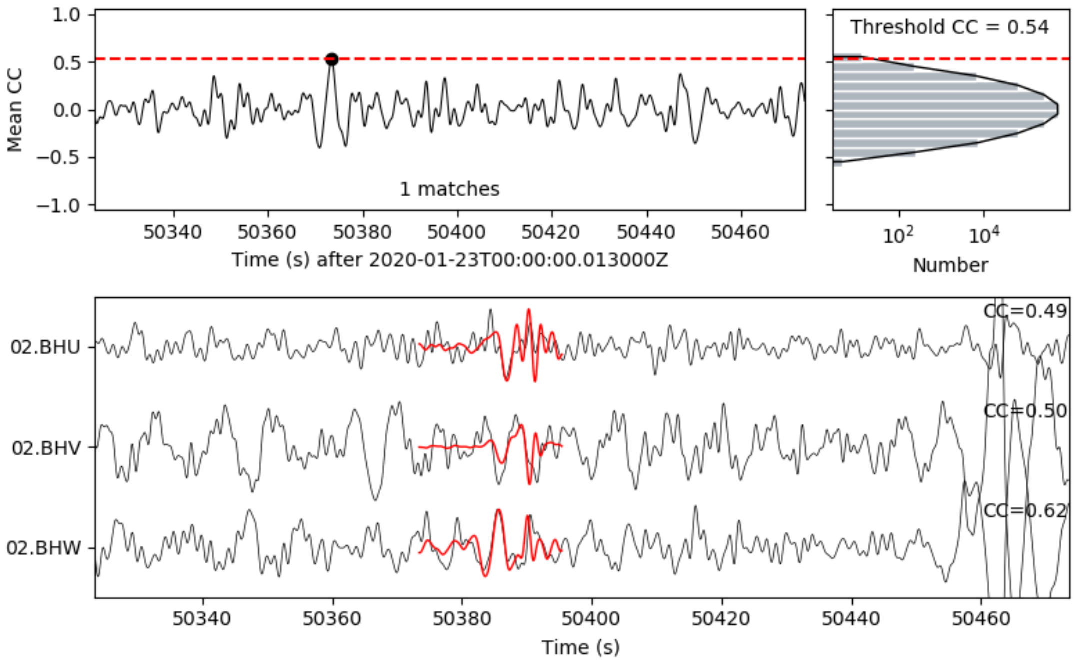

Supplementary Figure 44. The matched-filter detection of S0235b-MF30.

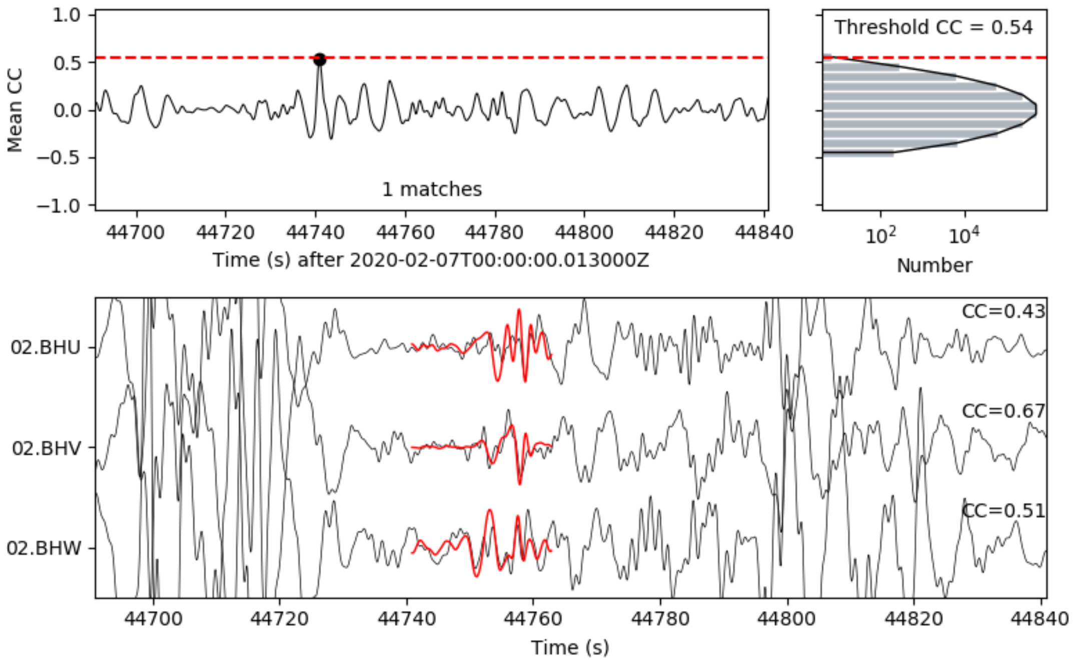

Supplementary Figure 45. The matched-filter detection of S0235b-MF31.

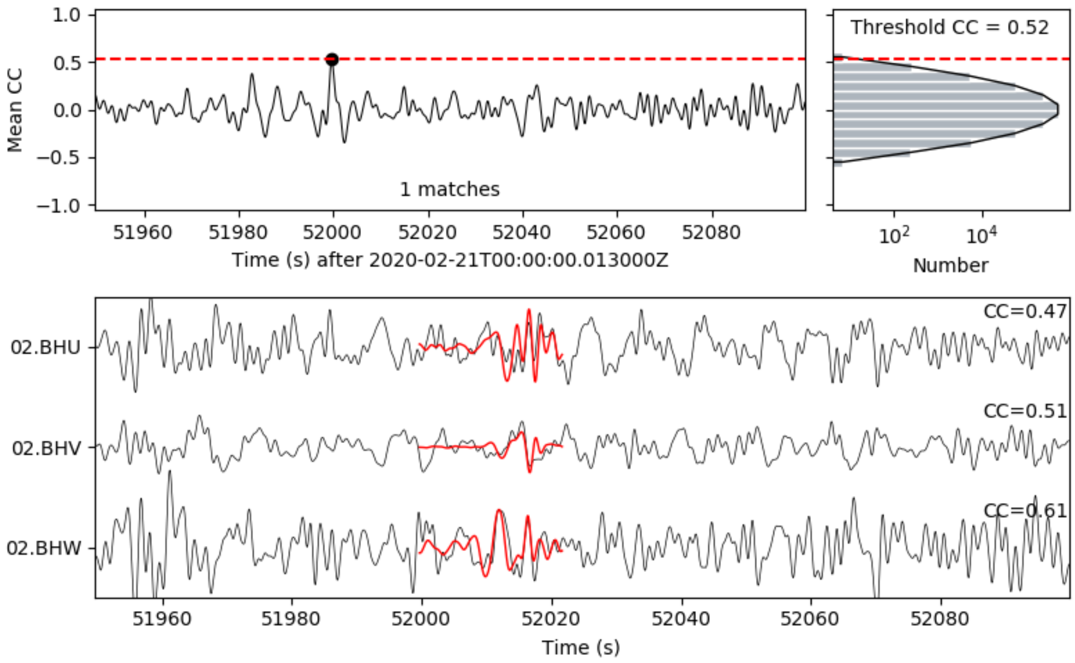

Supplementary Figure 46. The matched-filter detection of S0235b-MF32.

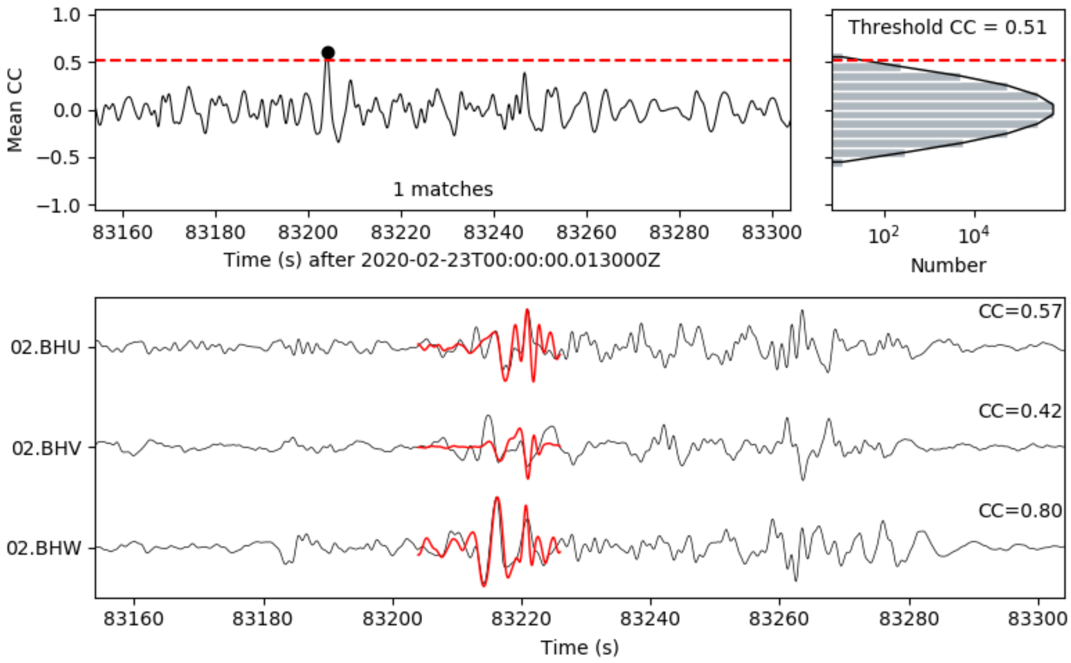

Supplementary Figure 47. The matched-filter detection of S0235b-MF33.

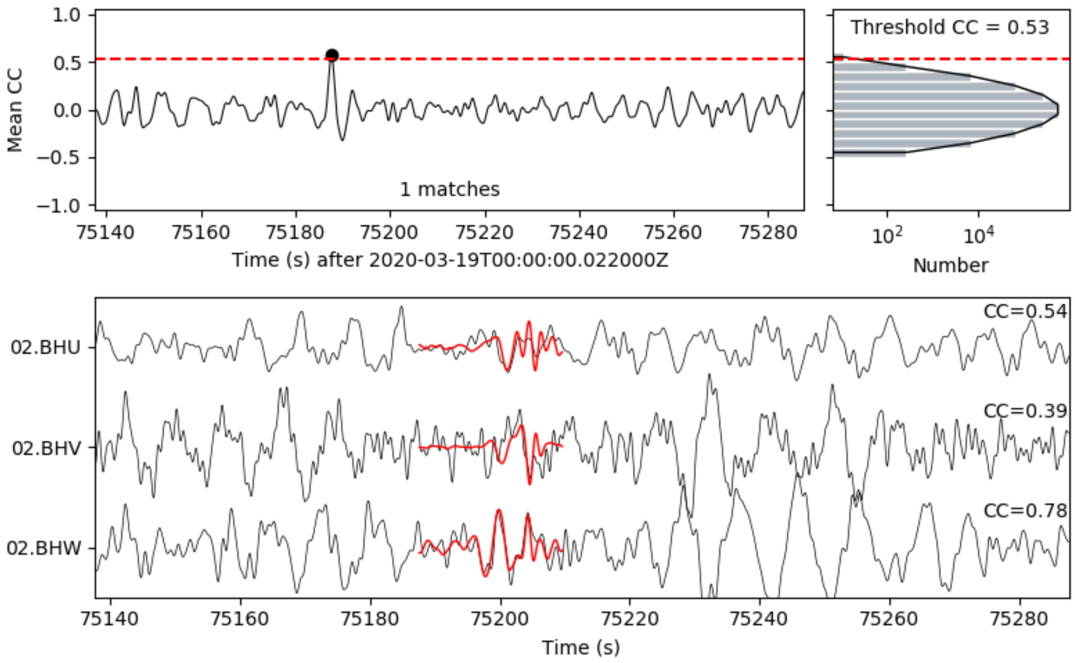

Supplementary Figure 48. The matched-filter detection of S0235b-MF34.

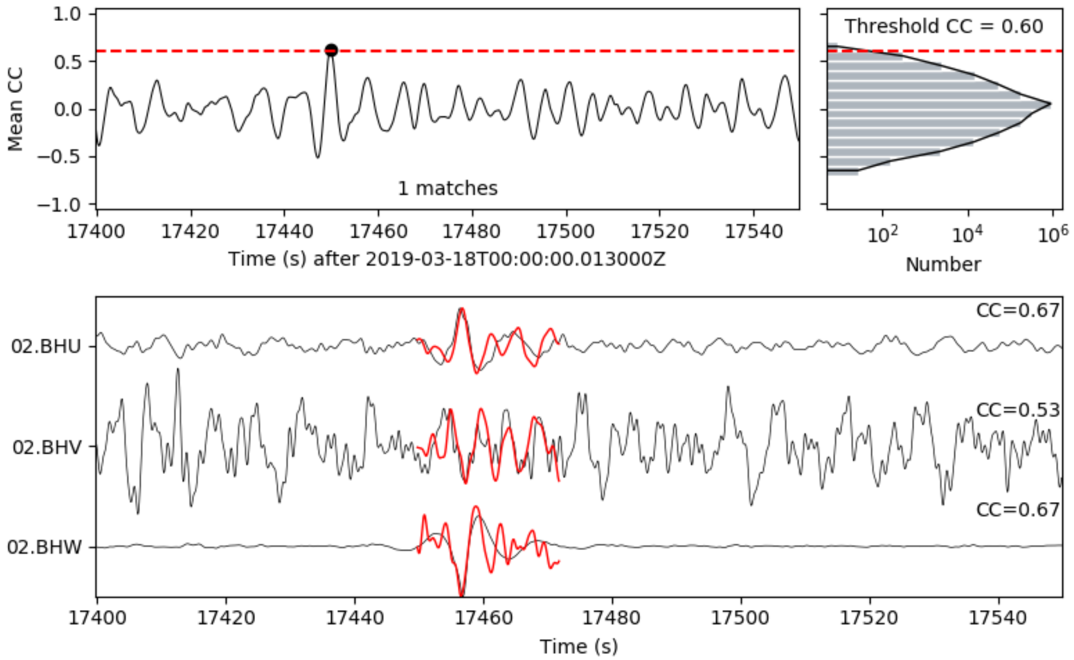

Supplementary Figure 49. The matched-filter detection of S0325a-MF01.

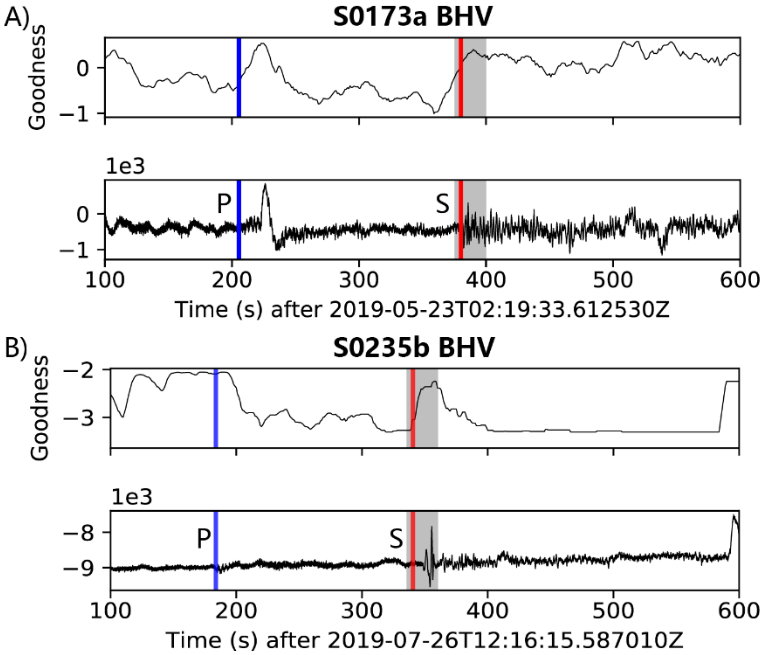

Supplementary Figure 50. Examples of Benford's Law (BL) tests for the two quality-A marsquakes from Supplementary Table 2. A) The S0173a event. The top panel shows the goodness of fit w.r.t. BL prediction (see Equation 1 in Materials and Methods). The definition of the dimensionless goodness of fit is also given in [5]. The bottom panel shows the original waveform recorded on 02.BHV component. Blue and red vertical lines mark the P and S arrivals. B) same as A, but for the S0235b event. The shaded time window is the template of marsquakes used in the MF method, shown here for reference.

1.2 Benford's Law

Supplementary Figure 50 shows the BL feasibility of the two A-quality marsquakes identified earlier: S0173a and S0235b. In this figure, we demonstrate how the BL predictions match the observations of P and S waves. For the first event (Supplementary Figure 50A), the apparent agreement of the P arrival with the BL theoretical prediction for the distribution of the ground-

velocity first-digits exists only because of a large-amplitude glitch about 25 s after the initial P-wave onset. In contrast, there is a sound performance of the BL prediction for the S-wave arrival. Supplementary Figure 50B shows that the second event's P-arrival amplitudes are comparable to the ambient noise, and BL prediction fails. The S arrival again is predicted well by the BL method.

Supplementary Note 2: Robustness of the matched filter method

The matched filter (MF) method has been successfully and extensively applied to detect low SNR earthquakes such as precursors, aftershocks, and volcanic events (see the references in the main part of the manuscript). Motivated by this success, we use the MF method to detect the marsquakes below the visual observational threshold, buried in the Martian seismic noise. In this section, we test the robustness of the MF method via synthetic and realistic Martian noise data, including the selection of key parameters.

2.1 The choice of the frequency band

Before matching the template waveform with the continuous waveforms, we apply a bandpass filter to both time series to improve the signal-to-noise ratio. An ideal frequency band should filter most of the noise out and keep most of the signal. In practice, it is challenging to find a perfect frequency band to differentiate between the seismic signal and noise in the Martian case.

Clinton, et al. [6] reported that the LF events are dominated by long periods (or low frequency) within a 0.1-1 Hz band. Scholz, et al. [7] analyzed the Ps receiver function of the S0173a event in the frequency band 0.1-0.8 Hz. Guided by this early work, we adopt the frequency band of 0.1-0.8 Hz as dominant for LF events.

Although the InSight SEIS is shielded from wind and thermal effects, temperature and atmospheric pressure variations could still seriously affect the seismic records. This is because the temperature and pressure variations may induce ground displacements. Garcia, et al. [2] investigated the behaviors of pressure signals in detail. Their spectrum analysis illustrated that the low-frequency seismic records are coherent with the atmospheric pressure in the frequency band 0.03-0.5 Hz during the Martian day. As seen in Figure 2 of Garcia, et al. [2], the largest coherence occurs at 0.1 Hz, and it rapidly decreases to a low level as the frequency becomes higher than 0.3 Hz at

night for the vertical component. The horizontal components also show similar phenomena; namely, the coherency is reduced significantly to a low level at night in the frequency band 0.1-0.3 Hz.

To select an appropriate frequency band, we perform a time-frequency analysis over the continuous waveform containing substantial amplitude variations from wind and temperature. We first use a single-day record, namely 1 July 2019, also used by Scholz, et al. [7]. Supplementary Figure 51 shows the unfiltered three-component waveforms and their corresponding spectrograms. It is evident that the raw waveforms are heavily perturbated in the time range of ~30,000-60,000 s due to the wind and pressure variations [7]. As seen from the spectrograms, these noise sources have a peak frequency of ~0.1-0.2 Hz and rapidly attenuate above 0.3 Hz.

In conclusion, the signals and noise have an overlapping frequency band, i.e., 0.1-0.3 Hz, and the potential marsquakes could be buried in the ambient noise. We apply a broad frequency band of 0.1-0.8 Hz to bandpass-filter low-frequency marsquakes and continuous waveforms.

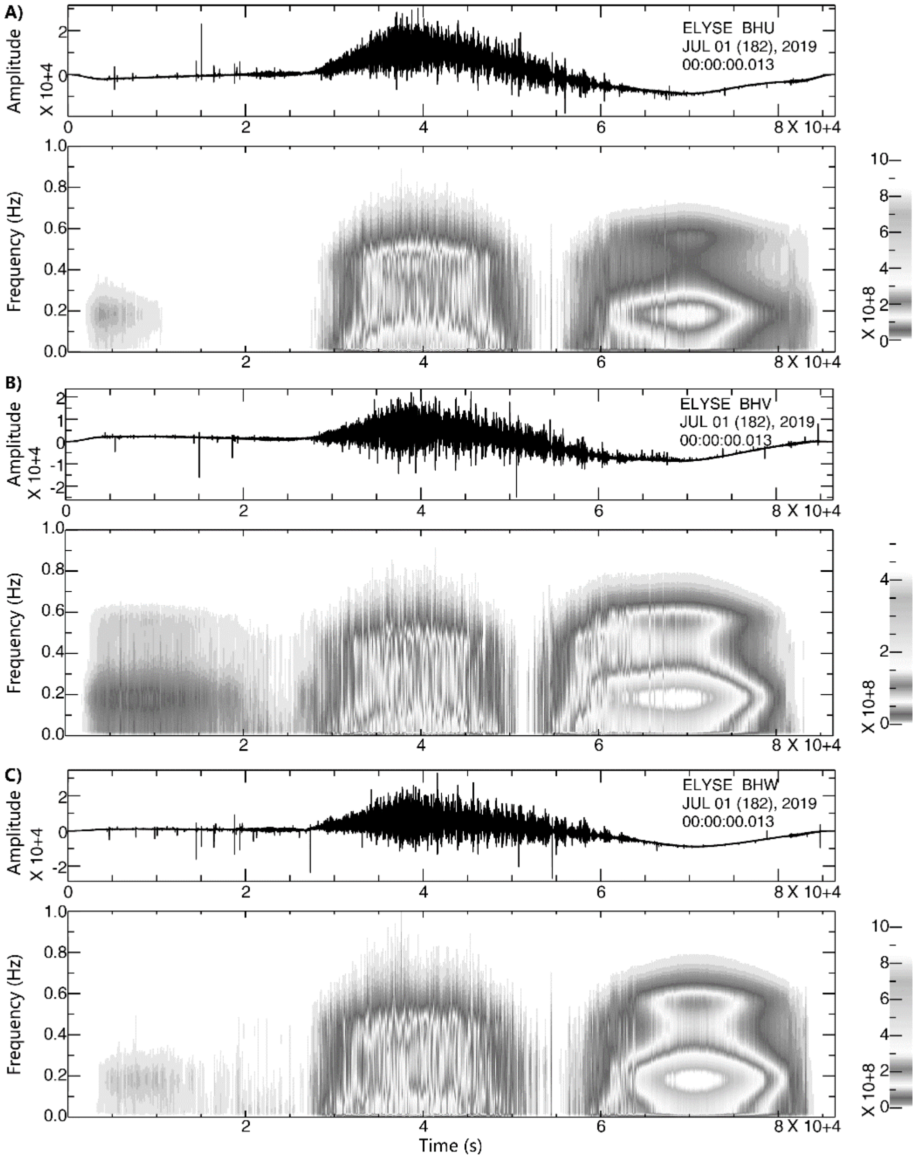

Supplementary Figure 51. The unfiltered one-day waveform and corresponding spectrogram on 1 July 2019. A), B) and C) show the three seismogram components: U, V, and W. In each subfigure, the top panel illustrates the raw waveform, and the lower panel shows the corresponding spectrogram.

To scrutinize the chosen frequency band further, we perform two bandpass filters: 0.1-0.3 Hz and 0.1-0.8 Hz and perform the self-detection tests of S0173a and S0235b marsquakes (Supplementary Figures 52 and 53). The length of the time window is required to be longer than twice the longest period, which will be discussed in Supplementary Note 2.5. In this case, the periods are T=1.25-10 s. We here take 22 s, a slightly longer time than twice the longest period, T=10 s. The window starts 2 s before and ends 20 s after the S arrivals given in Supplementary Table 1. For the frequency band 0.1-0.3 Hz overlapping with noise, our algorithm fails to self-detect the S0173a event (for selecting the threshold amplitude, see Supplementary Note 2.2). As seen in Supplementary Figure 52, the mean CC for the 0.1-0.3 Hz frequency band has a larger amplitude than the mean CC for the 0.1-0.8 Hz band, leading to the threshold of 1.004554, which is larger than the CC=1.0 (self-detection). Although the S0235b self-detection is successful for both frequency bands (0.1-0.3 Hz, 0.1-0.8 Hz), we also observe a similar phenomenon, i.e., the mean CC in the frequency band of 0.1-0.3 Hz is much larger than the one in the 0.1-0.8 Hz band.

The smaller mean CC for the broader frequency band test suggests the template signals have a lower correlation when higher frequency content is present, which is intuitive. In other words, when a higher frequency content is present, there is quantitatively less similarity between the template and the continuous waveform, but it is also easier to isolate the true similarity as it becomes more pronounced. We, therefore, apply a broad frequency band of 0.1-0.8 Hz for MF.

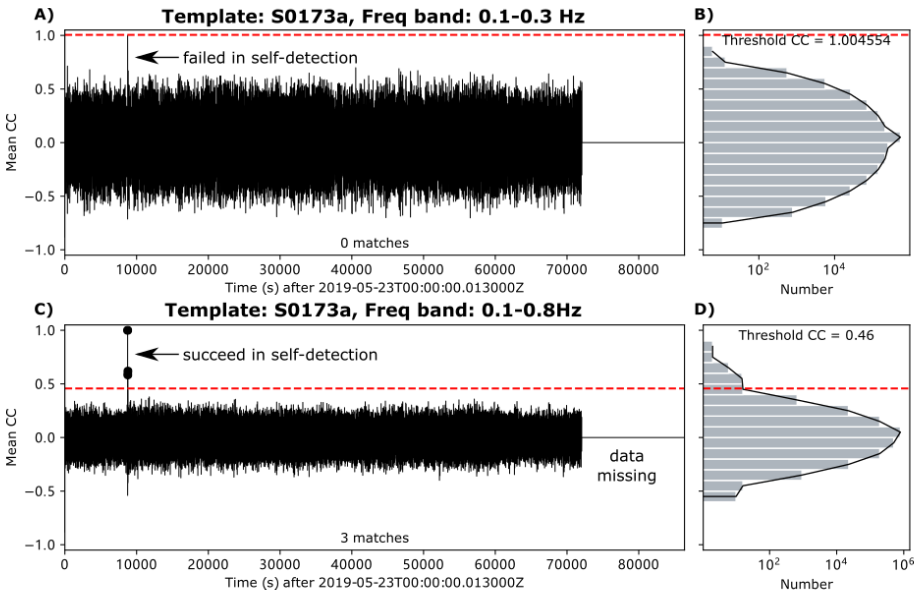

Supplementary Figure 52. Self-detection of S0173a for different frequency bands: A-B) 0.1-0.3 Hz, and C-D) 0.1-0.8 Hz. The left panel (A and C) illustrates the mean CC between the matching template of S0173a and the continuous waveform for 23 May 2019. The right panel (B and D) gives the histograms of the mean CC on the left.

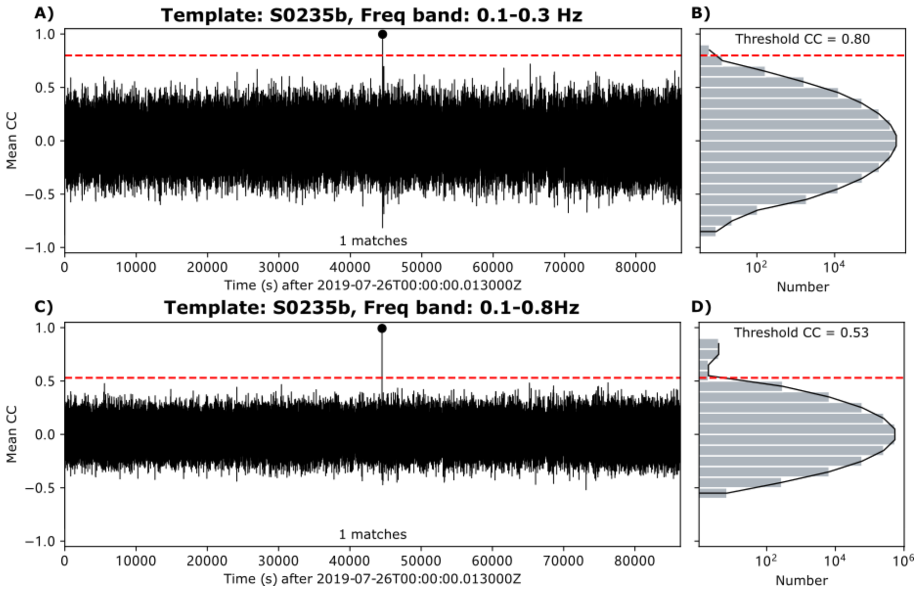

Supplementary Figure 53. Same as Supplementary Figure 52, but for S0235b.

2.2 The threshold of matched-filter detection

The successful detection of a Martian event mainly relies on the empirical selection of the threshold, as illustrated in Equation (2) of the main manuscript. Intuitively, a smaller threshold may result in more detections and vice versa. We here evaluate the selection of threshold via synthetic and realistic Martian noise data, accounting for the glitches in the Martian data time series. Our main criterion is that the noise (including the glitches) should not be detected as a possible event match.

The Martian noise mainly comes from the atmospheric pressure and temperature variations [7]. One ubiquitous feature of noise is the presence of the large-amplitude glitches caused by the SEIS-internal stress relaxation in the harsh temperature environment [7]. We generate white noise as a simulation of the wind perturbations, to which we applied the bandpass filter of 0.01-1 Hz. Three different types of glitches (see Figure 54) are trimmed from the continuous data on 23 May 2019. Supplementary Figure 54A shows the glitches on all three components, while Supplementary Figure 54B for a single component. The glitches in Supplementary Figure 54C demonstrate quite different frequency characters from those in Supplementary Figures 54A and 54B.

We keep the relative time and amplitude settings of the glitches the same as in the actual data. These glitches are then added to the white noise. Supplementary Figure 55A exhibits the white noise filtered in the frequency band 0.01-1Hz. We then apply the same filter as the MF processing to the white noise, and Supplementary Figure 55B demonstrates the summation of large-amplitude glitches and the white noise.

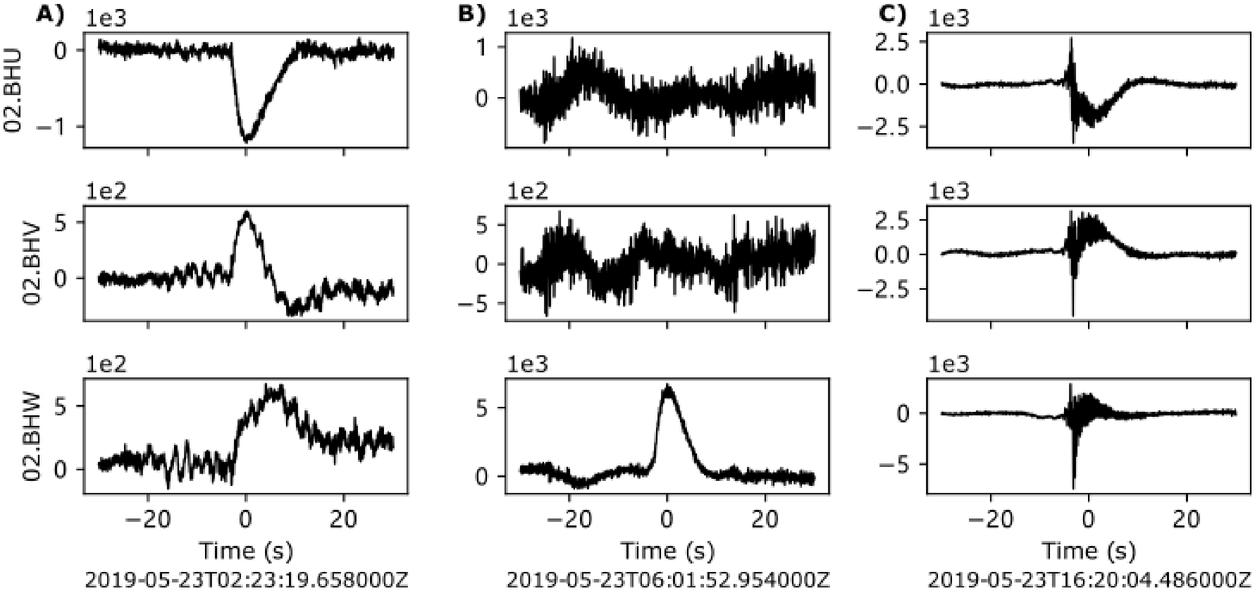

Supplementary Figure 54. The three types of glitches are extracted from real InSight SEIS data. The length of glitches is ~20 s, and the reference time is noted at the bottom of each subfigure. Please note the amplitude variations.

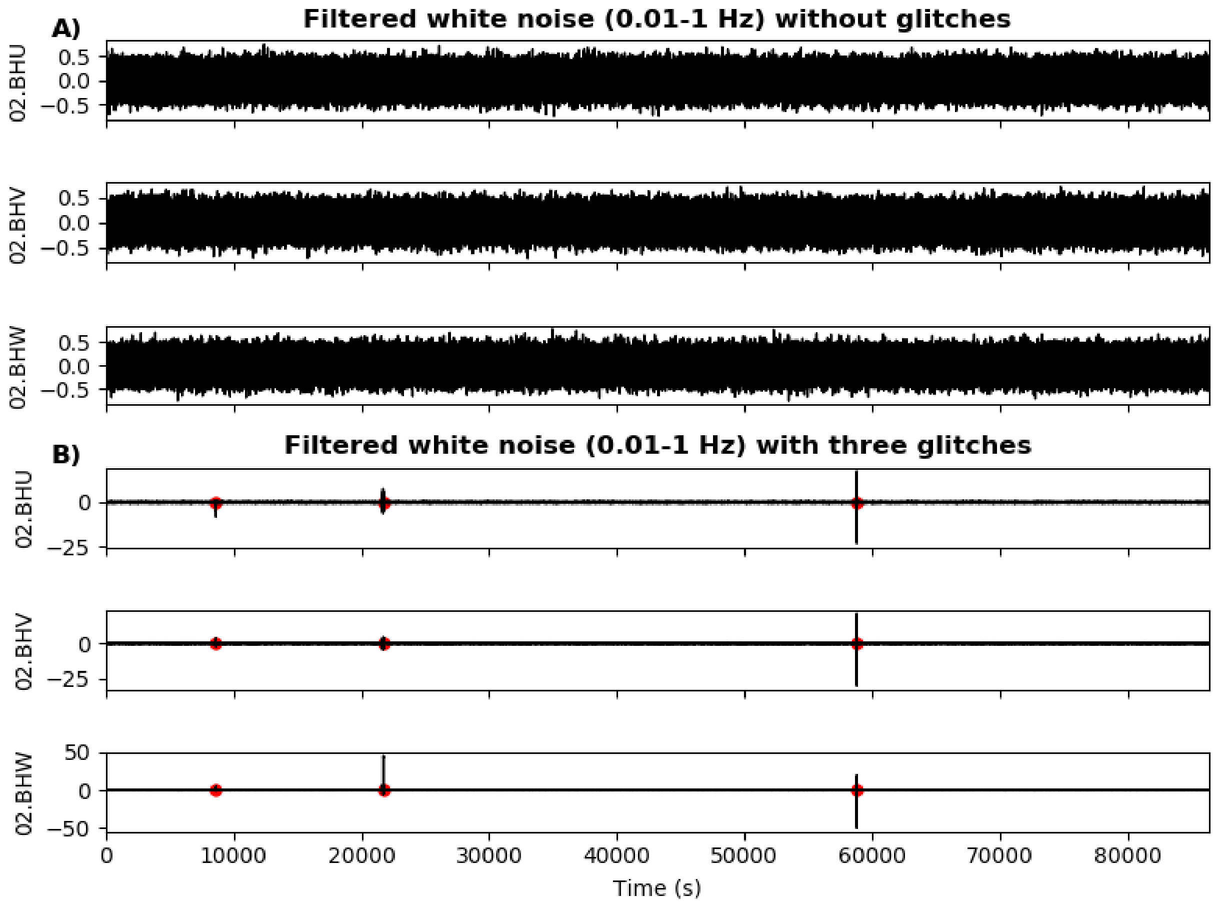

Supplementary Figure 55. Synthetic Martian ambient noise. A) white noise filtered between 0.01 and 1 Hz. B) Summation of the white noise with the glitches shown in Supplementary Figure 54. Note the amplitude variations. The relative amplitudes of the three glitches are the same as the ones in original Martian data.

We take the S0173a and S0235b as templates to match the synthetic noise in Supplementary Figure 55B and the realistic Martian noise on 1 July 2019 independently investigated by four groups [7]. Supplementary Figure 56 demonstrates the detections for the S0173a template using six- and seven-times MAD (for the definition of MAD, see Supplementary Note 1.1 and Equation 2). With the smaller threshold of 6 times MAD, five "fake detections" are identified, but significantly, they are unrelated to glitches (the blue dashed lines). This is also true for taking S0235b as a template in Supplementary Figure 57. We, therefore, found empirically that the threshold of 7 times MAD must be implemented for no "fake events" to occur.

We now switch from the synthetic to the actual Martian data. Supplementary Figures 58 and 59 illustrate the cross-correlation of the S0173a and S0235b templates with the noisy Martian data carefully analyzed by the ISAE, IPGP, MPS, and UCLA groups [7]. Like the synthetic experiment, the low threshold of 6 times of MAD produces "fake detections" while the high threshold of 7 times of MAD produces no "fake detections." Thus, we set 7 times of MAD as a threshold of MF detections based on synthetic and actual Martian data evaluations.

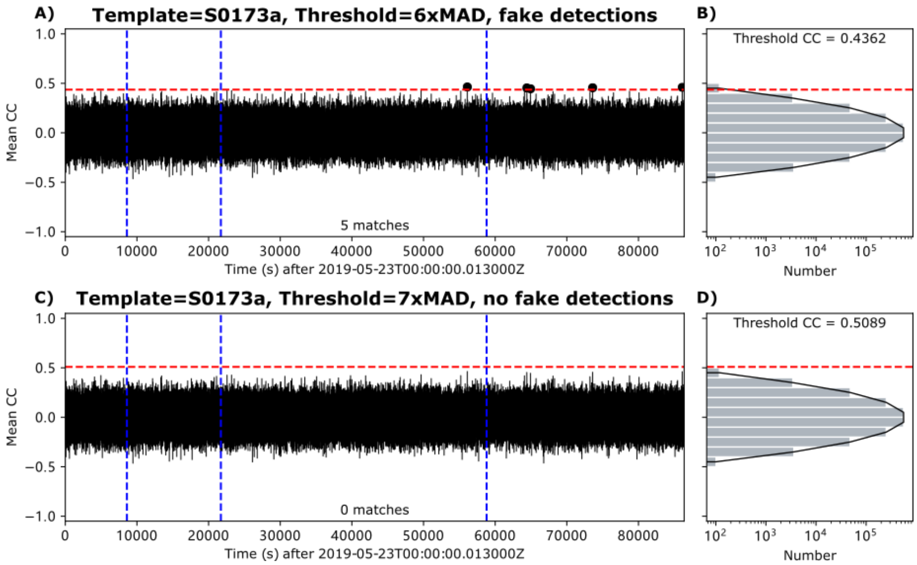

Supplementary Figure 56. Detections of the event S0173a using synthetic noise (white noise + glitches in Supplementary Figure 55B) with different thresholds: A-B) 6 times of MAD, and C-D) 7 times of MAD. The left panel (A and C) illustrates the mean CC between the template of S0173a and the continuous waveform on 23 May 2019. The right panel (B and D) gives the histograms of the mean CC on the left. The blue dashed lines indicate the position of the glitches introduced according to Supplementary Figure 54.

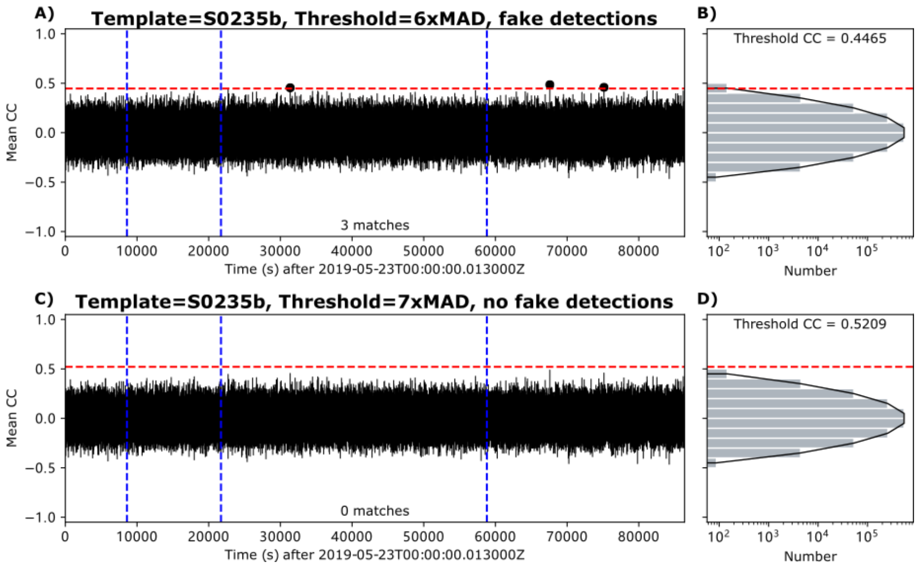

Supplementary Figure 57. Same as Supplementary Figure 56, but using S0235b as a template.

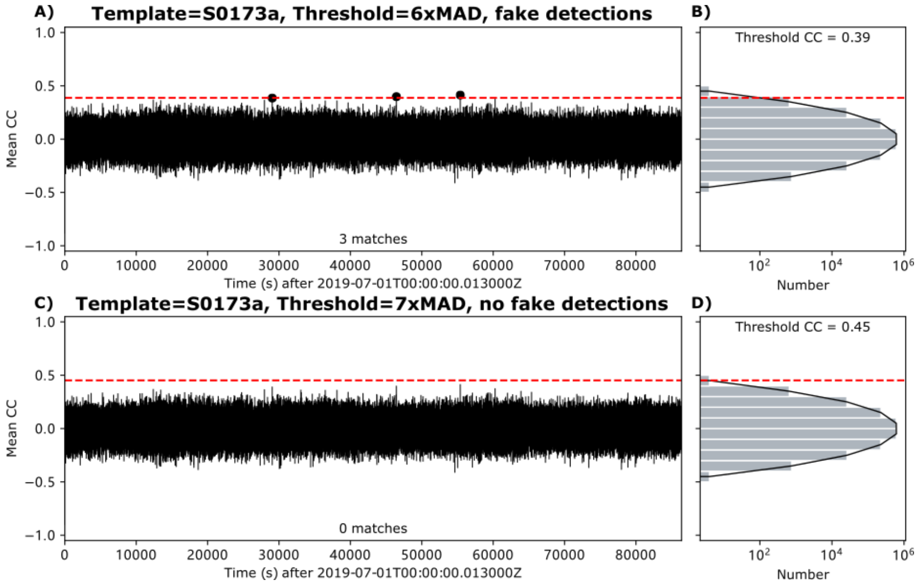

Supplementary Figure 58. Same as Supplementary Figure 56, but for the actual Martian data recorded on 1 July 2019. The three-component raw waveforms are shown in Supplementary Figure 51.

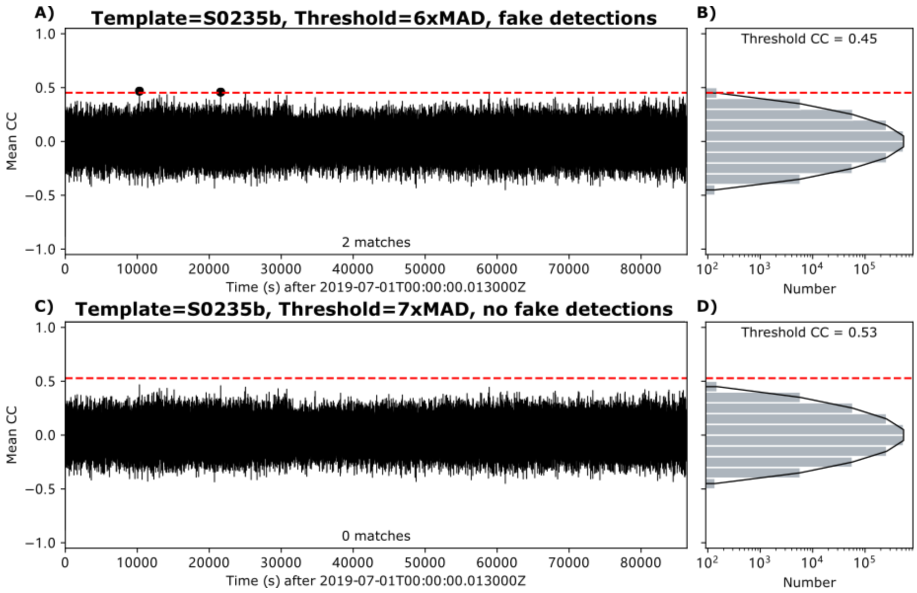

Supplementary Figure 59. Same as Supplementary Figure 57, but for the actual Martian data recorded on 1 July 2019.

2.3 The effect of glitches on MF detections

The raw Martian data is contaminated by large pulses of glitches resulting from the SEIS-internal stress relaxations in harsh temperature environments [7]. This section extends the experiments presented in the previous section to comprehensively assess the influence of glitches on MF detections through synthetic and actual noise.

We have embedded three glitches retrieved from the recorded data in the background white noise, as shown in Supplementary Figures 55. Taking S0173a and S0235b as templates, none of the glitches are detected as the newly detected events, as shown in Supplementary Figures 56 and 57.

We further test the detection across the 24-hour data on 1 July 2019, as illustrated in Supplementary Figures 60A~60C. About 140 glitches were detected by the MPS and ISAE groups, while around 250 glitches by UCLA and IPGP [7]. These glitches provided in the supplementary dataset of Scholz, et al. [7] are removed and shown in Supplementary Figures 60D~60F.

As in Supplementary Figures 58 and 59, no new events are detected, suggesting no influences of glitches on the MF detections, at least for the S0173a and S0235b templates. We further run the MF detections on the deglitched data on 1 July 2019 for S0173a and S0235b, as shown in Supplementary Figures S2.11. The test also demonstrates no detections. Overall, these assessments suggest the large pulses do not influence the MF detections, which means that the MF detections can be applied to the raw Martian data containing glitches.

Finally, we manually examine each newly-detected event to confirm that the glitches do not contaminate it.

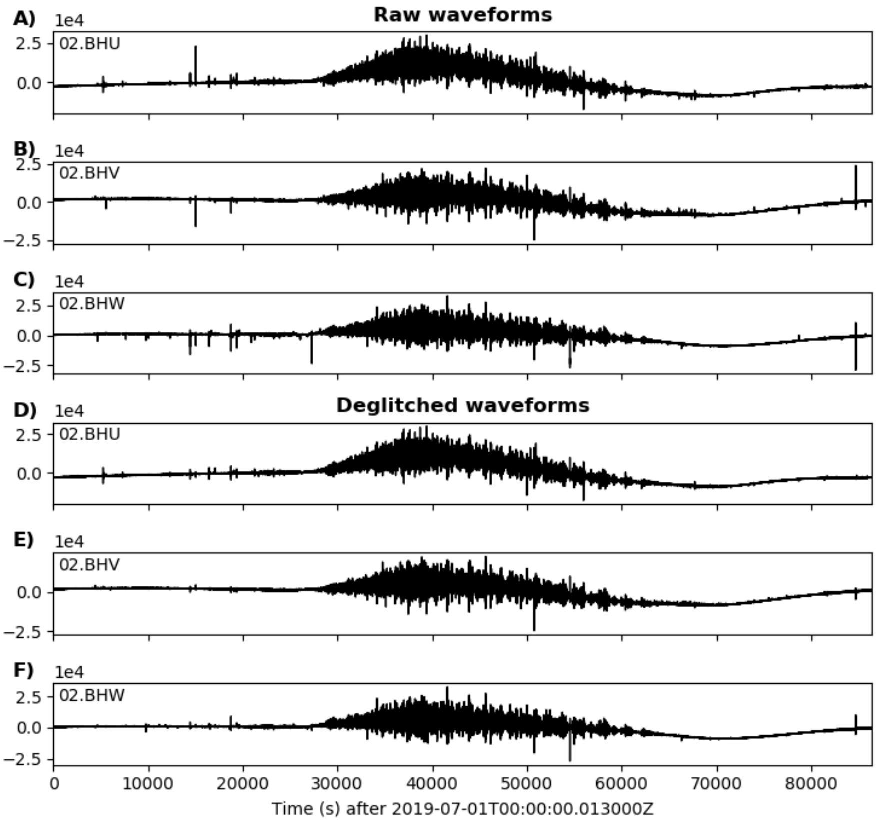

Supplementary Figure 60. The 24-hour Martian data on 1 July 2019. A-C) raw three-component waveforms of U, V, W; D-F) the deglitched waveforms of the U, V, and W components. The original amplitudes are shown.

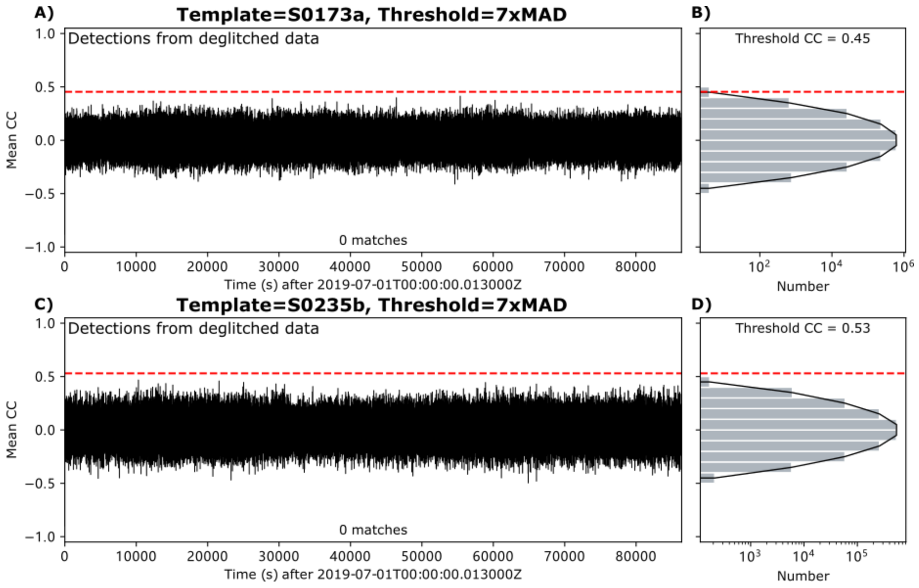

Supplementary Figure 61. MF detections over the 24-hour Martian data on 1 July 2019 using templates of A) S0173a and B) S0235b.

2.4 Tests of events buried in continuous noise

In this section, we embed the S0173a and S0235b events in the continuous noise. The noise is the same as in the previous tests. The start and end times of the two events are listed in Supplementary Table 3 (the durations are a bit over 33 minutes). The event waveforms are normalized according to the amplitudes in a 10-second-long time window starting 5 s before S arrivals. Then the amplitudes of S arrival are scaled to 1.0, 0.75, and 0.5 times of the maximum noise amplitude (normalized to 1). The resulting sum waveforms of the synthetic noise waveforms and the events are shown in Supplementary Figures 62 to 64. As seen, the event waveforms contain large-amplitude glitches, which allows systematic tests of background noise and real glitches.

The corresponding MF tests for S0173a are given in Supplementary Figures 65 to 67. For the test with the S-wave scale set to 1.0, two new detections are observed, of which one is a self-detection, and the other is the S0173a-MF01. As the scale is set to smaller values, 0.75 and 0.5 (the noise becoming more significant), only the self-detection is observed with the cross-correlation coefficients reduced significantly. For example, the cross-coefficients of the self-detection reduce from 0.68 to 0.30 when the scale is changed from 1.0 to 0.5 for the 02.BHU component of S0173a (Supplementary Figures 65D and 67D).

The MF tests for the S0235b template embedded in the synthetic noise are illustrated in Supplementary Figures 68 to 70. The MF technique successfully performs the S0235b self-detection. In addition, we observe a similar property, i.e., for a more substantial noise, the cross-correlation coefficients are smaller. However, the decrease is relatively mild, from 0.94 to 0.88 for the 02.BHU component of S0235b (Supplementary Figures 68D and 70D).

Overall, the tests suggest that the MF method can work well for moderate noise but cannot identify those weak marsquakes or seriously contaminated by ambient noise.

Supplementary Table 3. Start and end time of the waveform containing the S0173a and S0235b events embedded in continuous noise.

Event name	Origin	Start time	End time
S0173a	2019-05-23T02:19:16	2019-05-23T02:02:53.632000Z	2019-05-23T02:36:13.632000Z
S0235b	2019-07-26T12:15:45	2019-07-26T11:59:35.605000Z	2019-07-26T12:32:55.605000Z

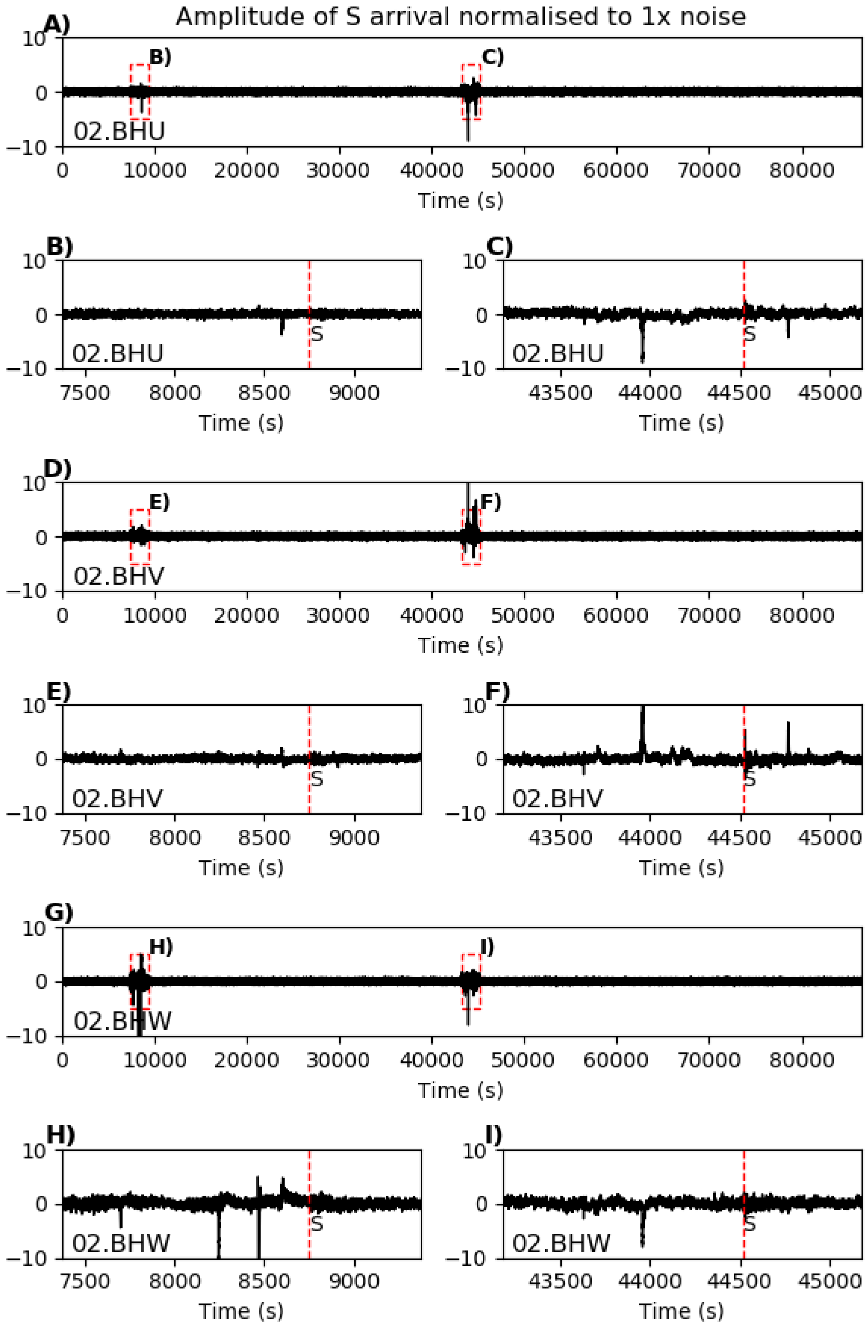

Supplementary Figure 62. The events S0173a and S0235b embedded in the synthetic background noise for three components: A)-C) U component, D)-F) V component and G)-I) W component. The three-component waveforms marked by red rectangles are magnified in B), E) and H) for S0173a, and in C), F) and I) for S0235b. The S arrivals are marked by red dashed

lines. The amplitudes of S waves are normalized to 1.0 with respect to the maximum background noise amplitude.

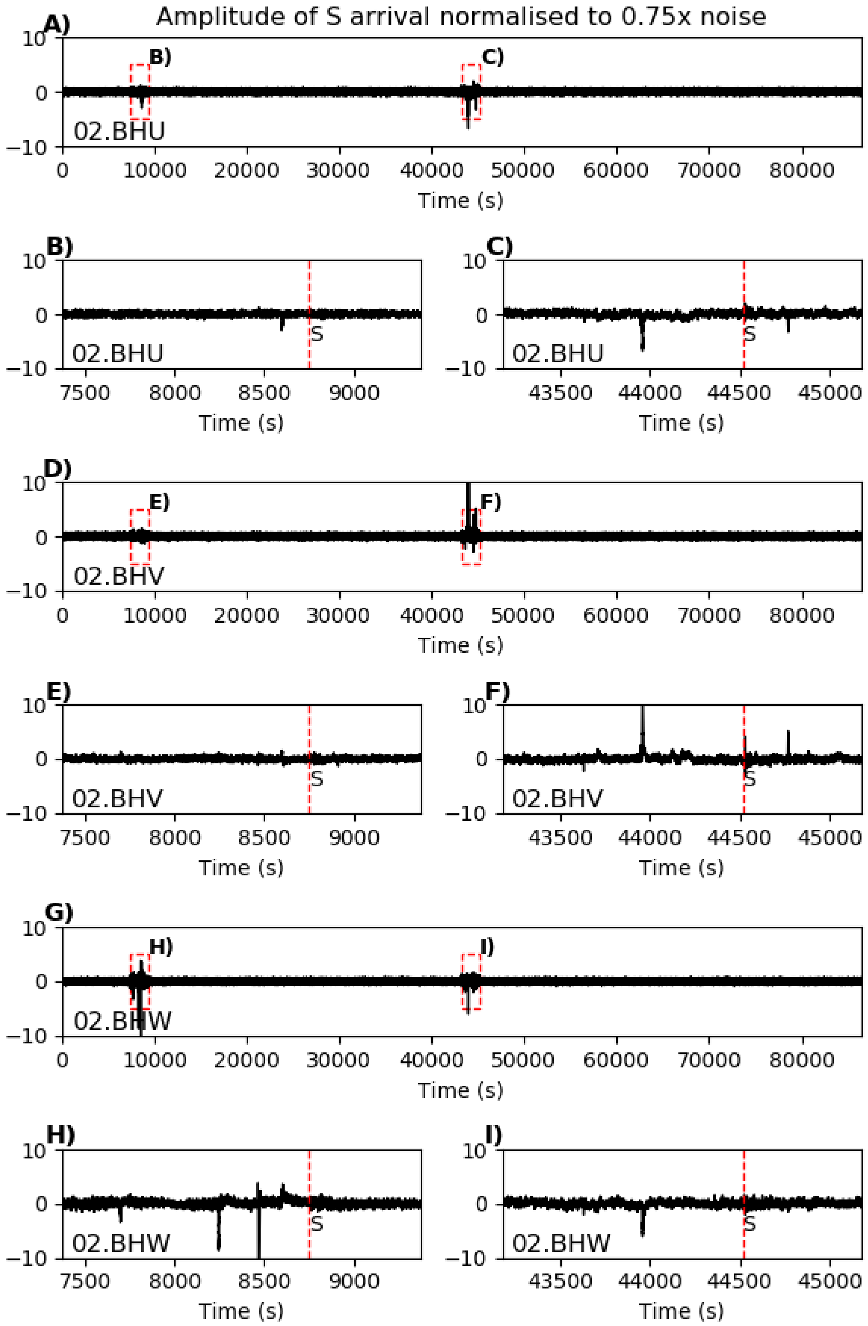

Supplementary Figure 63. Same as Supplementary Figure 62, but the amplitudes of S waves are normalized to 0.75 times with respect to the maximum background noise amplitude.

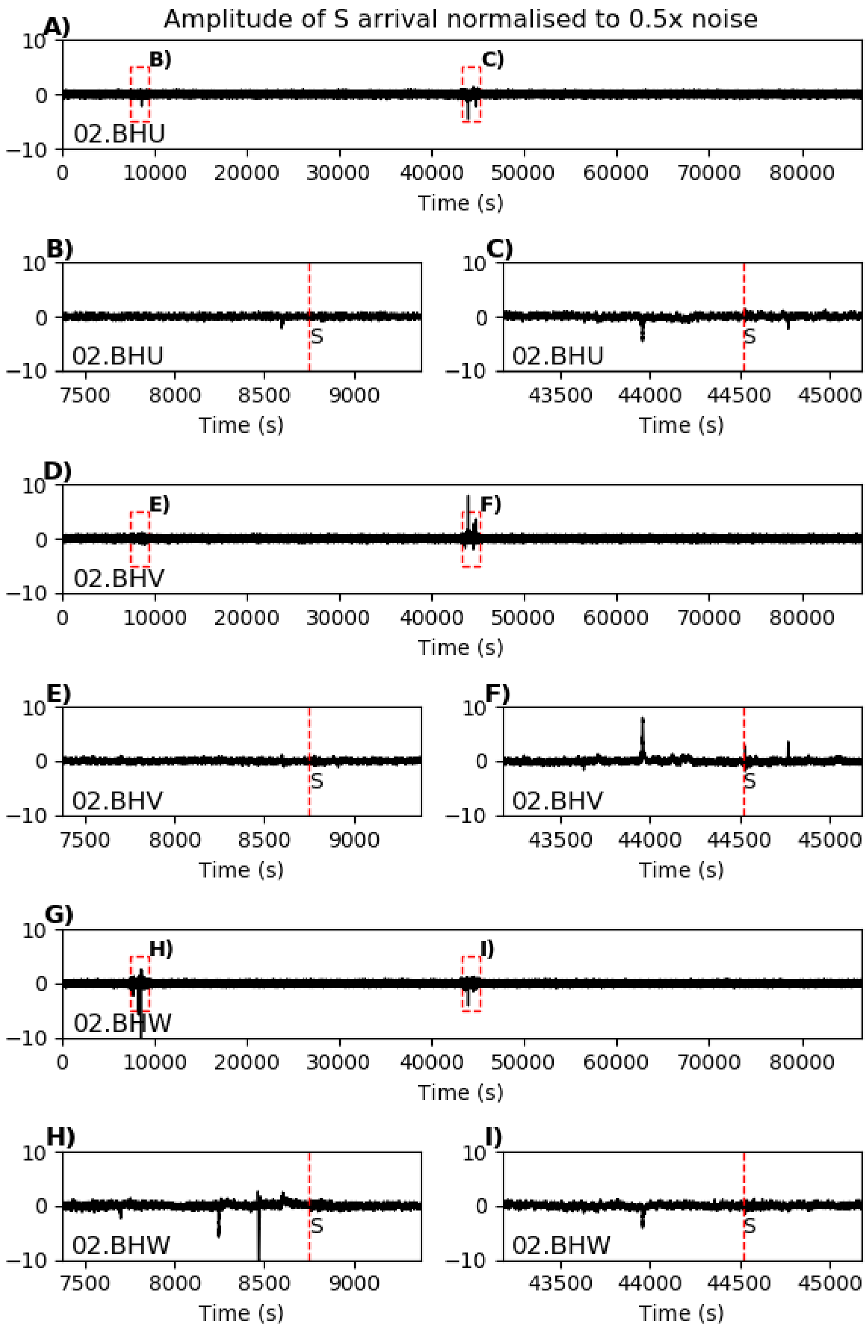

Supplementary Figure 64. Same as Supplementary Figure 62, but the amplitudes of S waves are normalized to 0.5 times with respect to the maximum background noise amplitude.

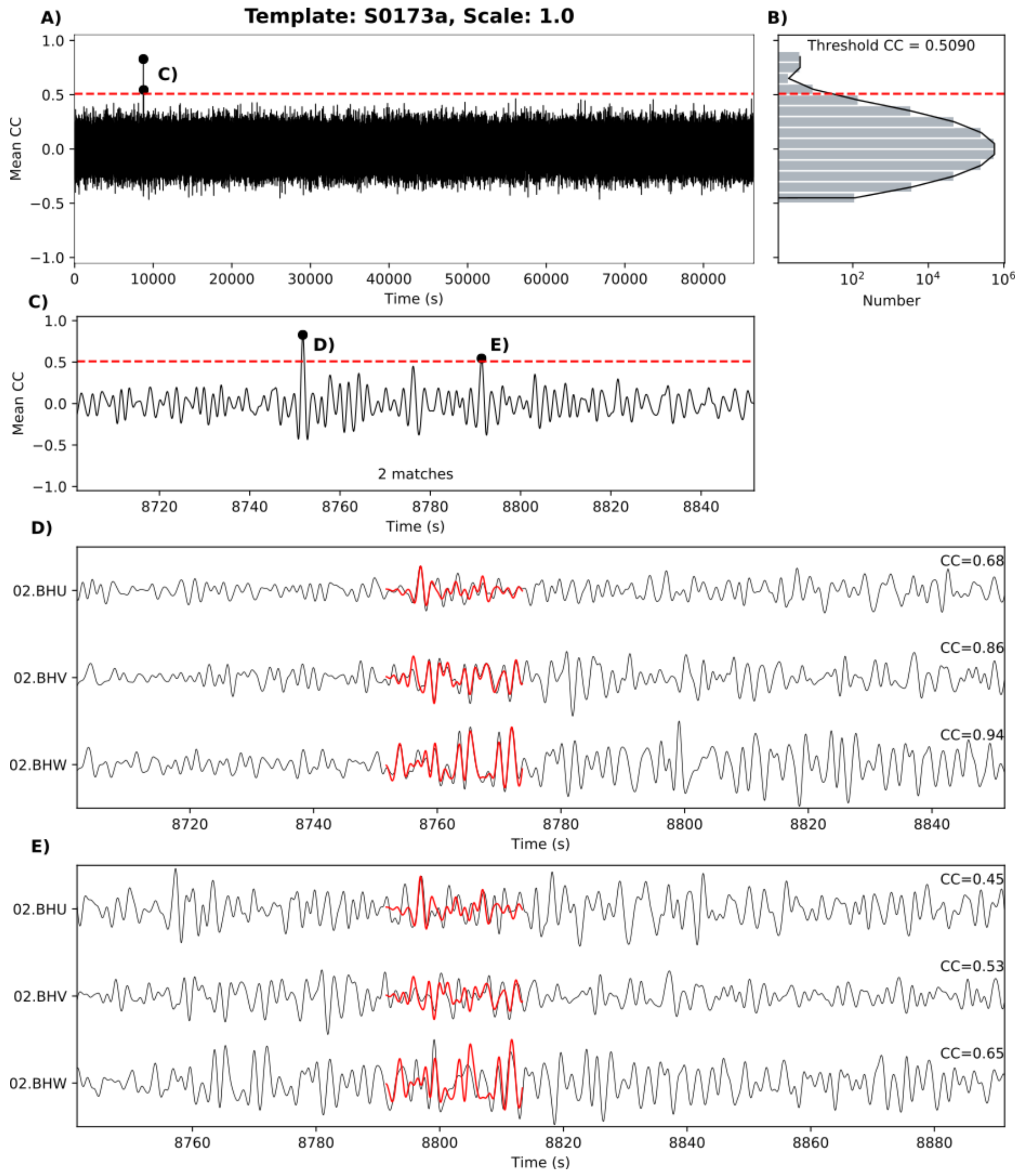

Supplementary Figure 65. The MF detections using the template of S0173a and the continuous waveforms as shown in Supplementary Figure 62. The amplitudes of S waves are normalized to 1.0 of the maximum noise amplitude. A) The averaged cross-correlation function. The dots illustrate the values and timings of positive event detection above the threshold (red dashed line). B) The histogram of the averaged cross-correlation functions with the same vertical axis as A). C) The magnification of A). D) and E) The template and continuous waveforms match. Red lines

are the template event waveforms. The trace names are labeled on the left. The cross-coefficients of each component are listed on the right.

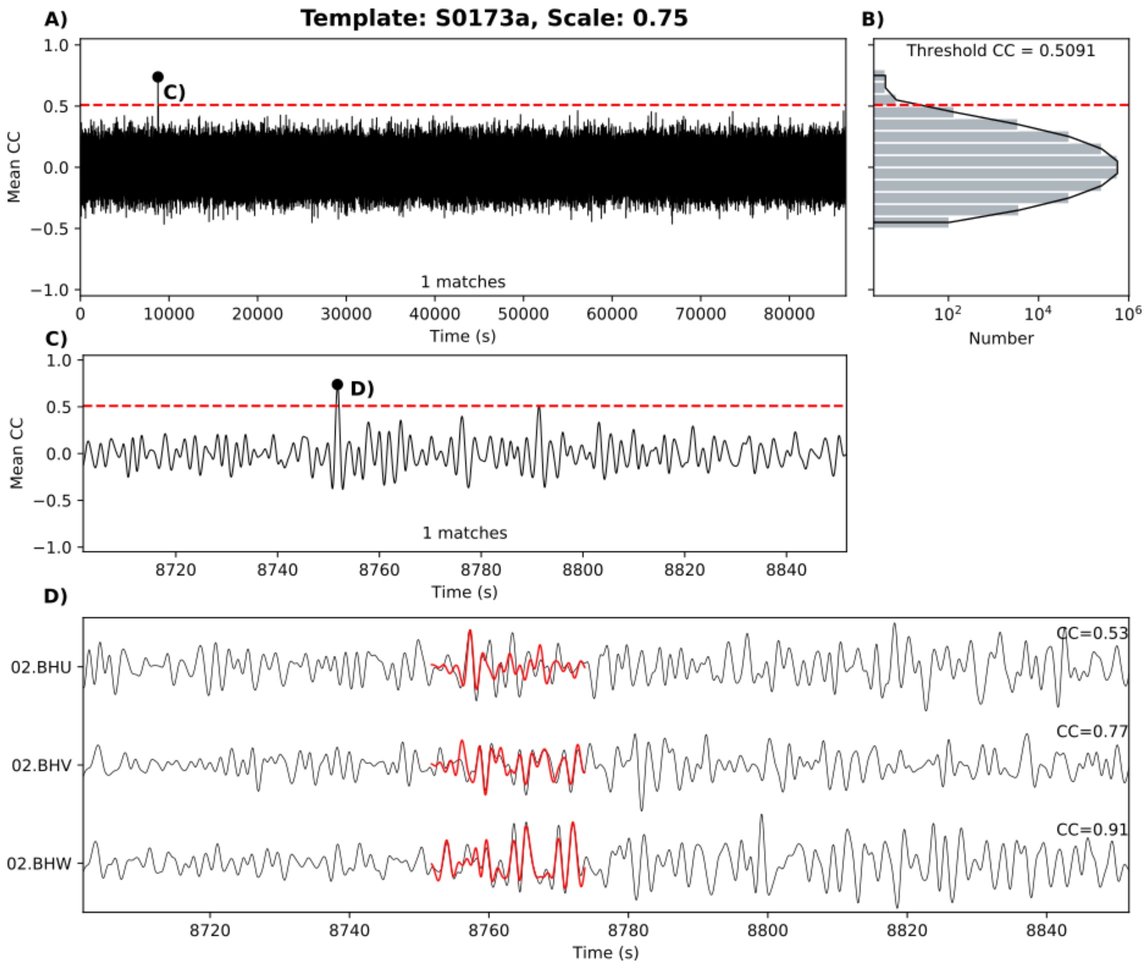

Supplementary Figure 66. Same as Supplementary Figure 65, but the amplitudes of S waves are normalized to 0.75 times of the maximum noise amplitude.

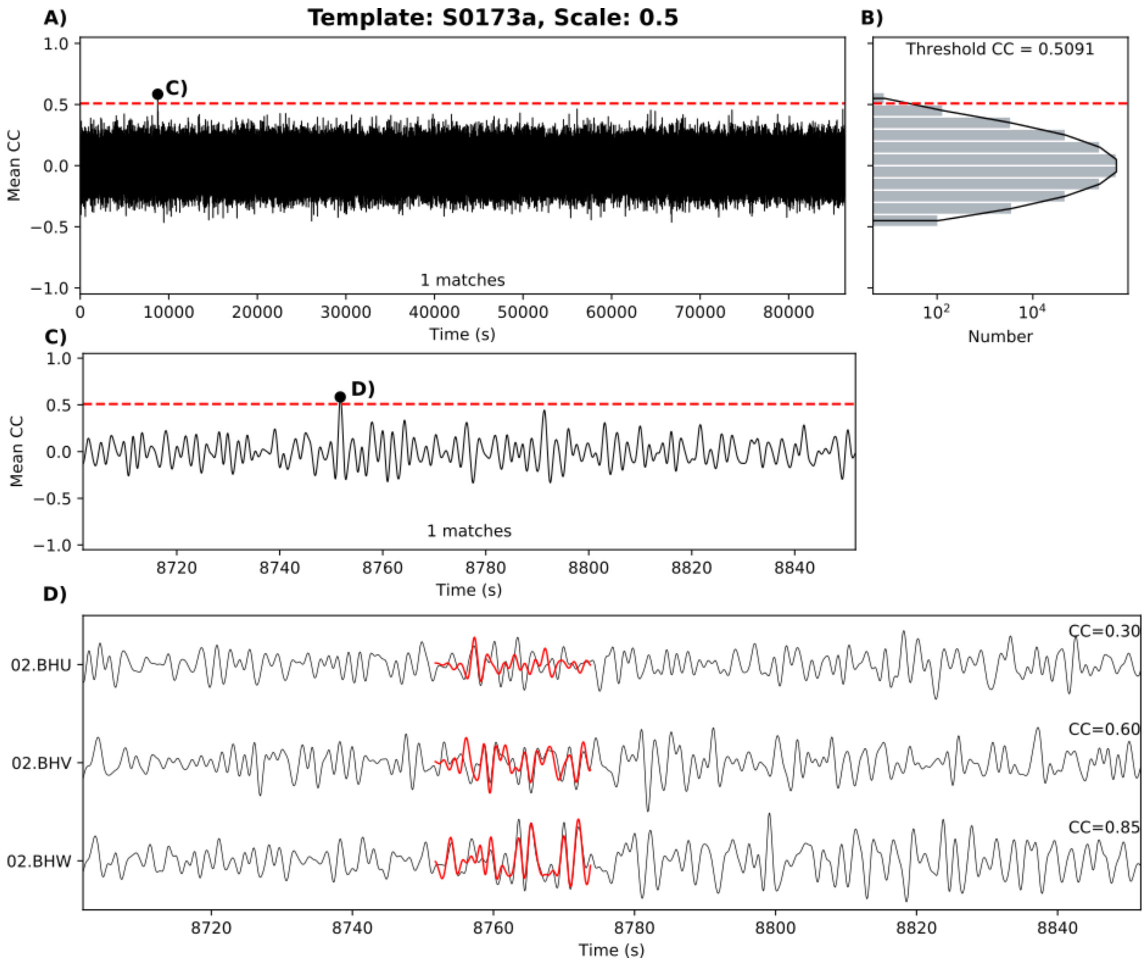

Supplementary Figure 67. Same as Supplementary Figure 65, but the amplitudes of S waves are normalized to 0.5 times of the maximum noise amplitude.

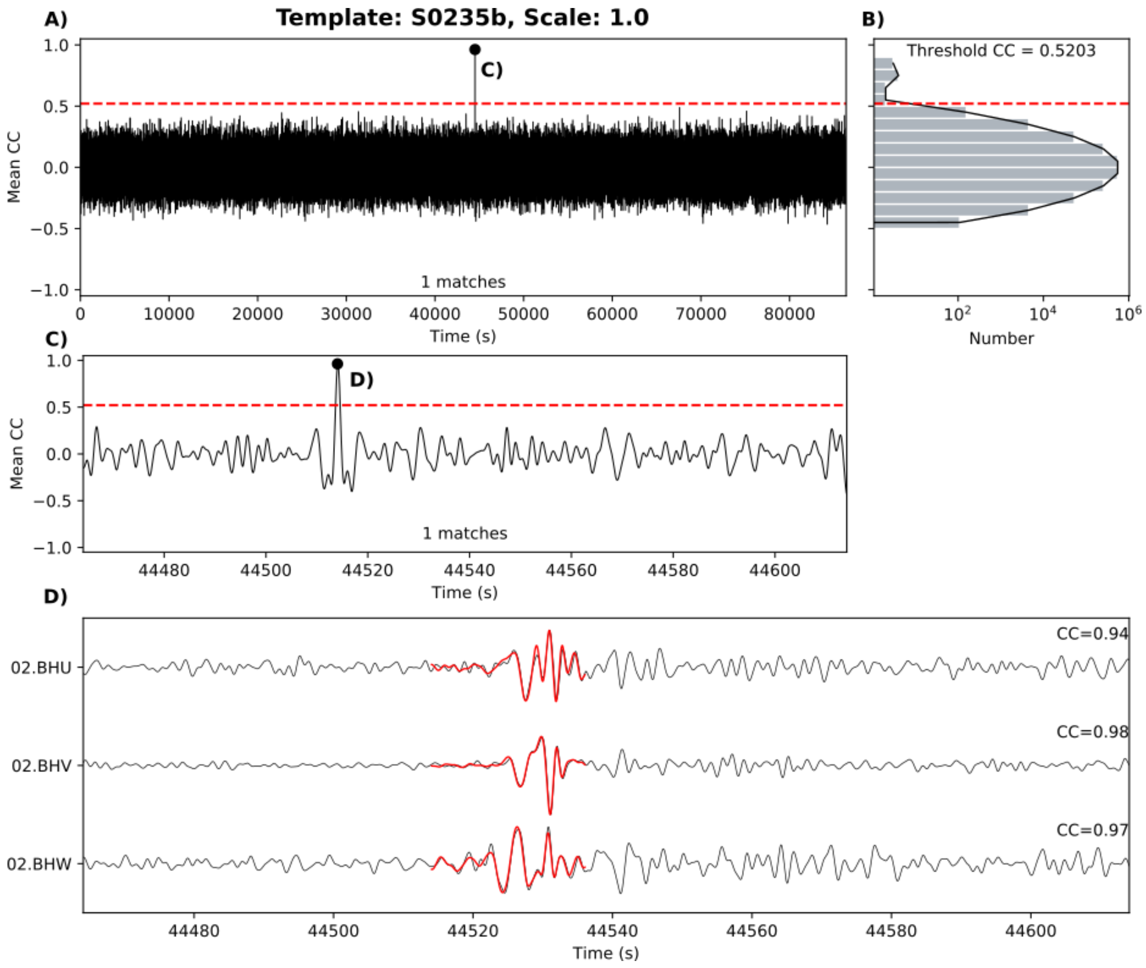

Supplementary Figure 68. The MF detections using the template of S0235b and the continuous waveforms as shown in Supplementary Figure 62. The amplitudes of S arrivals are normalized to 1.0 of the maximum noise amplitude. A) The averaged cross-correlation function. The dots illustrate the values and timings of positive event detections above the threshold (red dashed line). B) The histogram of the averaged cross-correlation functions with the same vertical axis as A). C) The magnification of A). D) The template and continuous waveforms match. Red lines are the template event waveforms. The trace names are labeled on the left. The cross-coefficients of each component are listed on the right.

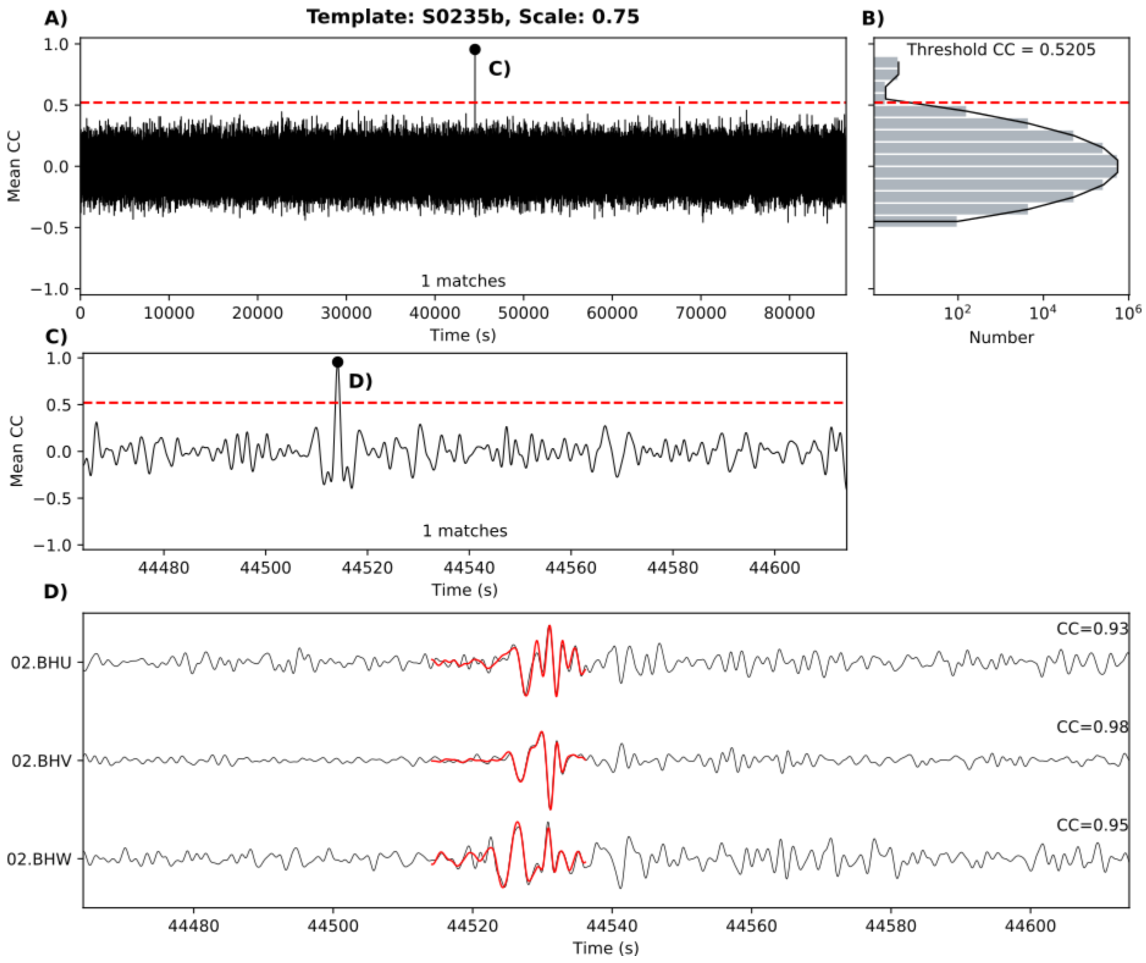

Supplementary Figure 69. Same as Supplementary Figure 68, but the amplitudes of S arrivals are normalized to 0.75 times of the maximum noise amplitude.

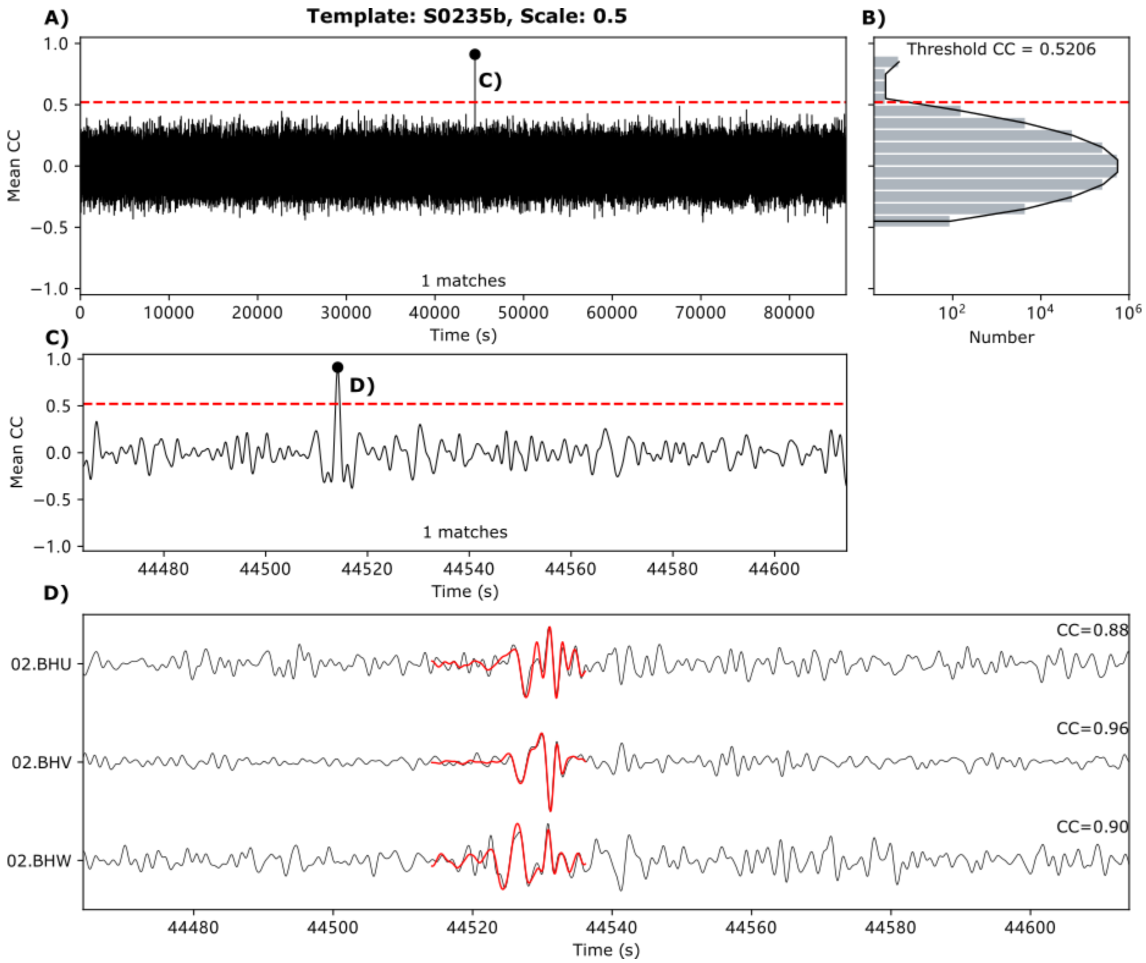

Supplementary Figure 70. Same as Supplementary Figure 68, but the amplitudes of S arrivals are normalized to 0.5 times of the maximum noise amplitude.

2.5 The effect of the template length

Empirically the length of the time window is set to be twice the longest period. The frequency band is 0.1-0.8 Hz (1.25-10 s). Thus we take 22 s, a slightly longer time window than twice the longest period (10 s). This choice is driven by the S-wave arrival uncertainties of ± 2 s for S0173a and S0235b. Ideally, the template should contain a minimal amount of noise before the S-wave arrivals. Furthermore, the template length should not be too long to minimize the weight of the late part of the waveforms affected by coda scattering. We empirically determine that the template should start 2 s before and end 20 s after the S arrivals listed in Supplementary Table 1.

We demonstrate the effect of increasing the template length by about 20% for S0173a and S0235b in Supplementary Figures 71 and 72. The template lengths are 22 s and 26 s, starting 2 s before the S-wave arrival. The second template, therefore, includes 4 s longer coda or noise than the first one. For the S0173a event, three detections in total are observed for both template lengths: the self-detection, S0173a-MF01, and S0173a-MF02 (Supplementary Figures 71A and 71D). The longer the template, the smaller the detection threshold. For 22 s length, it is 0.4573, and for 26 s length, it is 0.4313.

We perform the same evaluation for the S0235b template on the continuous data of 1 November 2019. Two detections (S0235b-MF14 and S0235b-MF15) are recorded for template length of 22 s in Supplementary Figures 72A to 72C. However, a single detection (S0235b-MF15) is recorded for the template length of 26 s in Supplementary Figures 72D to 72F. This is because the longer time window contains more coda scattering from local heterogeneities and thus more noise, leading to the failure of MF detections. Intuitively, this is expected because longer template lengths will include more S-wave coda from local heterogeneities and ambient noise, leading to the corresponding cross-correlation coefficient's reduction.

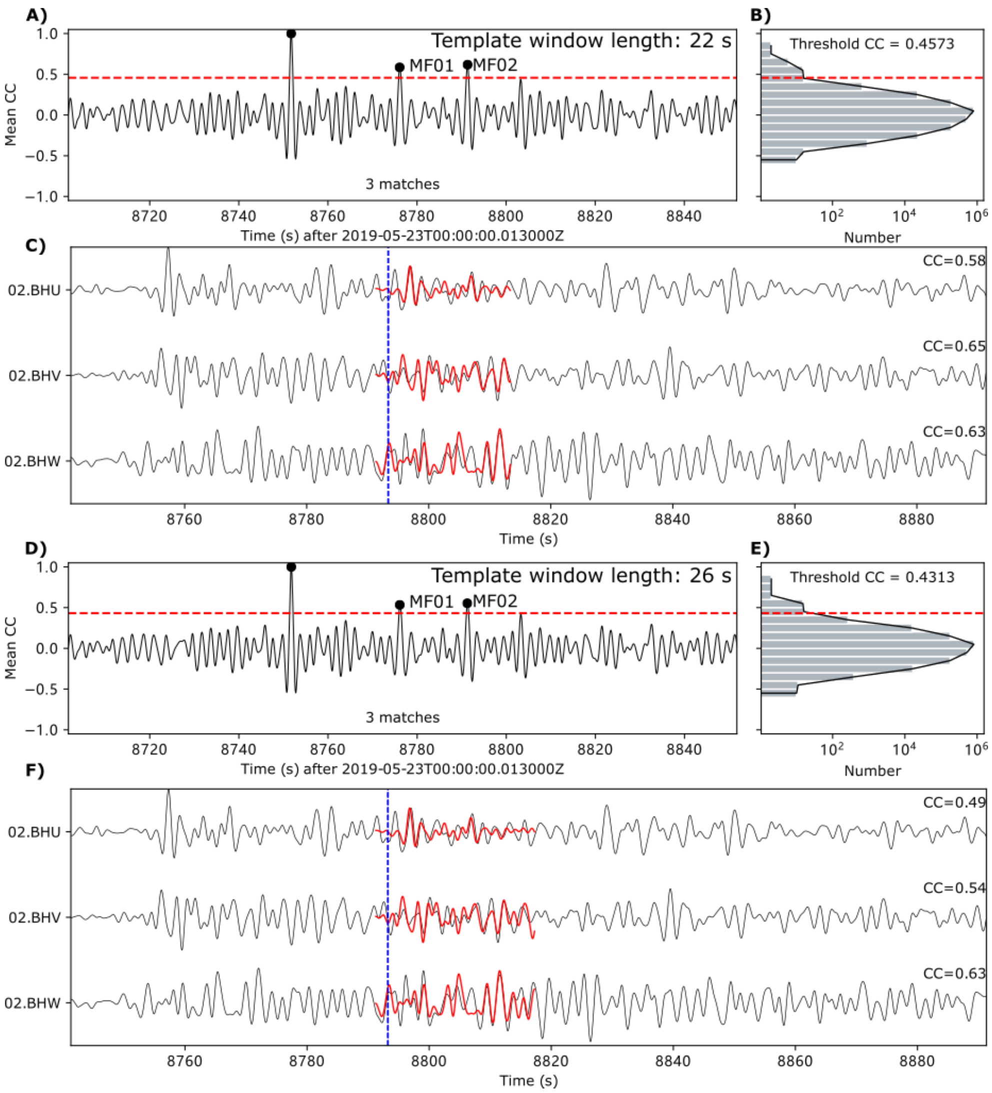

Supplementary Figure 71. The MF detections with two different lengths of template windows for the S0173a event. A) the averaged cross-correlation function with the dots illustrating the values and timings of positive event detections above the threshold (red dashed line). B) the histogram of the averaged cross-correlation function in A). C) the continuous record matched by the S0173a template. D)–F) Same as A)–C) but for the template window length of 26 s. Three identical events were detected using both template lengths (the self-detection, MF01, and MF02).

The start time of the template is 2019-05-23T02:25:51.8, which is 2 s before the reported S-wave arrival of S0173a.

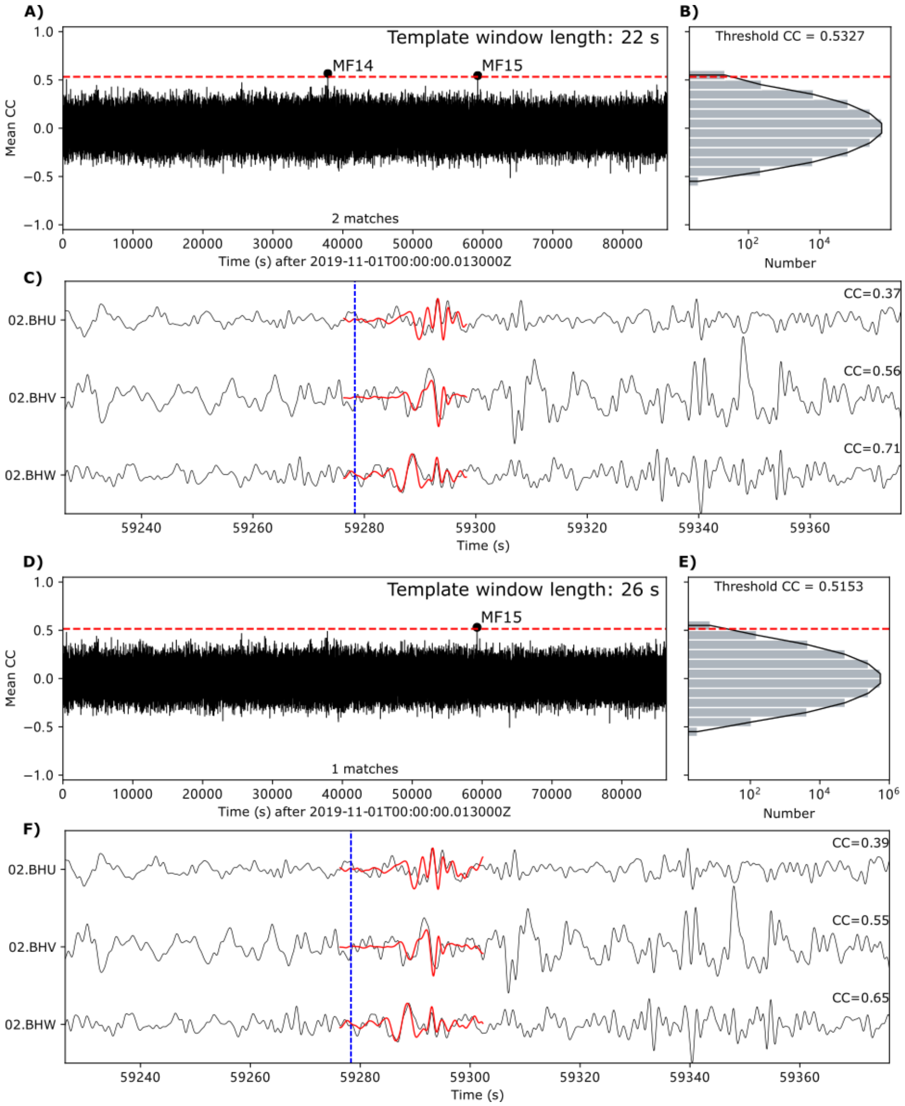

Supplementary Figure 72. Same as 71, but for the S0235b event. Two events (MF14 and MF15) were detected with the 22 s template length, while only M15 was detected with the 26 s template length. The template start time is 2019-07-26T12:21:54.1, i.e., 2 s before the reported S-wave arrival of S0235b.

2.6 The effect of the template start time

Due to the low signal-to-noise ratio, the S-wave arrivals of marsquakes are picked with significant uncertainties for different marsquakes. The seven B-quality marsquakes in Supplementary Table 1 have substantial phase-arrival uncertainties, between ± 10 s and ± 20 s. Only the S0173a and S0235b events, characterized as the quality A events, have relatively small phase-arrival uncertainties of ± 2 s.

As most detections in Table 1 originate from the two A-quality events, S0173a and S0235b, we perform additional tests with a fixed template length of 22 s (see Supplementary Note 2.5 for the effect of template length), but the template start time is varied as 0 s, $\pm 1s, \pm 2s, \pm 3s$ and $\pm$ 6s relative to the S arrivals. This deliberately spans a broader range than the $\pm 2s$ phase-arrival uncertainties for the events S0173a and S0235b. Shifting templates to too early times will result in the addition of unwanted ambient and event-generated noises (P-wave coda) to the S-wave while shifting templates to the later times will result in losing the beginning of the S-wave waveforms. We intuitively expect that shifting to a later time, i.e., losing the first part of S-waves and adding more coda, will result in the failure of detections. On the other hand, shifting the template to an earlier time will effectively reduce the template length containing marsquake signals and may result in more detections.

Supplementary Figure 73 illustrates the total number of detections for various template start times. The template start times of -2 s and -1 s have similar detection numbers, i.e., 47 and 45. The start times of -6 s and -3 s give the highest number of detections, i.e., 118 and 68. The increased detection might be due to the reduced S-wave coda content or increased content of S to P converted phases arriving before S waves. For earlier start times (e.g., -30 s, -40 s, -60 s), the number of detections decreases to a small number. Finally, the detection number drops to zero

when only the noise template (starting and ending just before the P-wave arrivals) is considered (see Supplementary Figure 73). In contrast, the number of detections with the template start time of 0, 1, 2, 3, and 6 s is lower, likely due to the inclusion of the S-wave coda.

Other factors affecting detection come from atmospheric conditions such as pressure, wind direction, and wind speed [6]. In addition, the S0235b marsquake excited higher-frequency coda scattering (up to 4 Hz) that attenuated faster than S0173a [6].

In summary, our tests indicate that the parameters such as the frequency band, the template start time and duration, all influence the MF detections. Other important factors that might influence detections are the magnitude, the focal mechanism, the source-time function, the properties of the medium through which the wavefield propagates, and the ambient noise. We have probably not exhausted all factors that can influence the results, but the tests performed here are comprehensive, and we adopt the parameter settings for the MF method given in Supplementary Note 1.

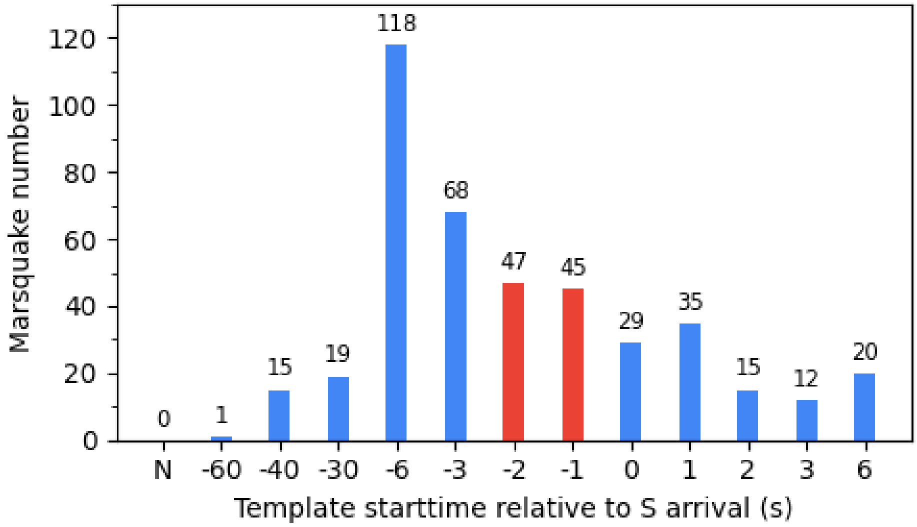

Supplementary Figure 73. The MF detections for various start times of the template relative to S arrivals, except for the label N. The label N denotes noise with the template [-24, -2] s relative to P-wave arrivals reported in Supplementary Table 1. The number of marsquake detections is highlighted in red for the start time of -2 s (our preferred template start time) and -1 s. The template window length is 22 s.

Supplementary Note 3: Cross-correlation coefficients, signal-to-noise ratios, detected vs. template events amplitude ratios and their relationship with the local mean solar time

The signal-to-noise ratio (SNR) in this study is calculated as the ratio of the maximum amplitudes of signal and noise in specific time windows. The times [-2, -10] s relative to P-wave arrivals and [-2, 20] s relative to S-wave arrivals are defined as "the signal windows." The times [-40, -2] s and [-80, -2] s relative to P and S arrivals, respectively, are considered "the noise windows." The amplitude ratio is calculated using the median amplitude values over all channels given in Equation 5 in Methods. We present a set of figures which examine the relationships of the parameters from Table 1.

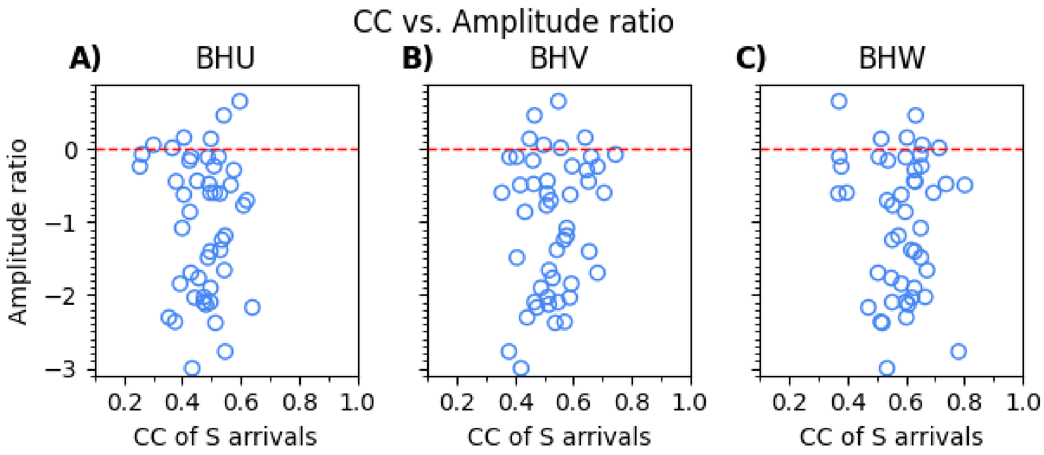

Supplementary Figure 74. The relationships between the normalized cross-correlation coefficients (CC) and MF detected vs. template amplitude ratios for three different seismograph components: A) BHU, B) BHV, C) BHW. The red dashed lines illustrate the amplitude ratios of the template events, which equals zero according to Equation 5.

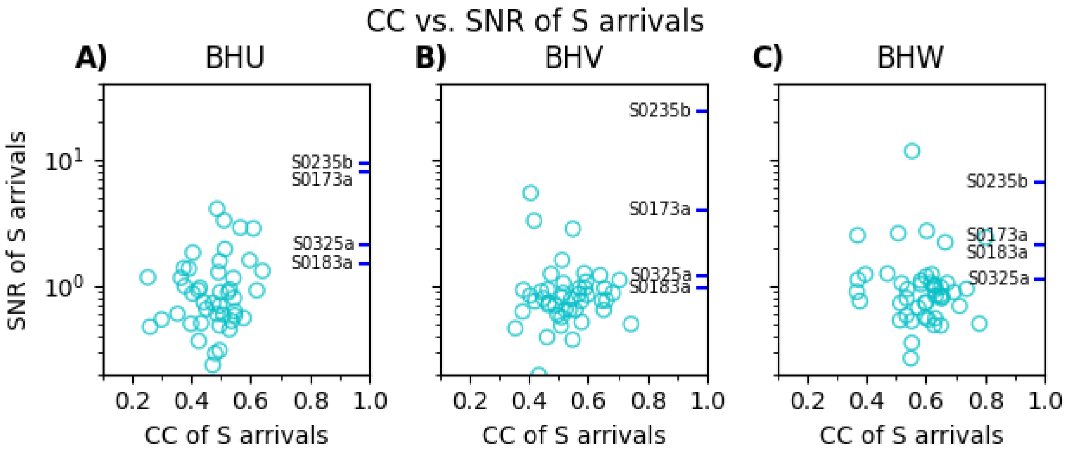

Supplementary Figure 75. The relationship between normalized cross-correlation coefficients (CC) and signal-to-noise ratio (SNR) of MF detected S-wave arrivals for three different seismograph components: A) BHU, B) BHV, C) BHW. The blue dashes illustrate the SNR of the template events (as reported in Table 1) with their names labeled.

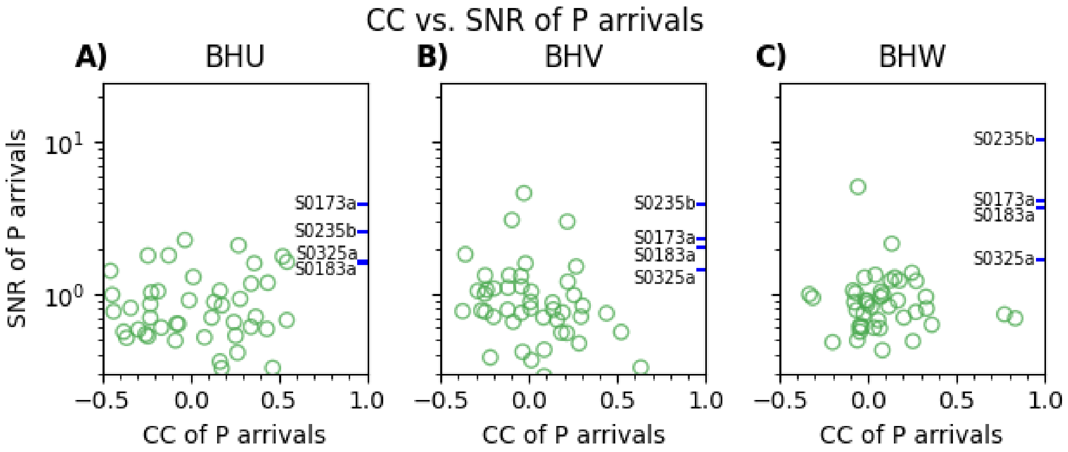

Supplementary Figure 76. The relationship between normalized cross-correlation coefficients (CC) and signal-to-noise ratio (SNR) of MF detected P-wave arrivals for three different

seismograph components: A) BHU, B) BHV, C) BHW. The blue dashes illustrate the SNR of the template events (as reported in Table 1) with their names labeled.

In Supplementary Figure 74, we show the amplitude ratios (Equation 5) of all detected vs. template events as a function of the measured cross-correlation coefficients (Equation 3) for each event. Most cross-correlation coefficients are between 0.3 and 0.8, and the amplitude ratios are between -3.0 and slightly above 0.0. We show these amplitude ratios as a function of local mean solar time (LMST) in Supplementary Figure 81.

In Supplementary Figures 75 and 76, we demonstrate the relationship between the cross-correlation coefficients and SNR. It is apparent that the majority of the MF-detected events' SNRs are smaller than those of the two most significant template events: S0235b and S0173a. For example, the S-wave arrival of S0235b has an SNR as high as 32 on the BHV component, whereas the P-wave arrival of S0235b has SNR as high as 19 on the BHW component. The cross-correlation coefficients P waves are unsurprisingly smaller than those of S waves and have higher and less scattered values on the BHW component.

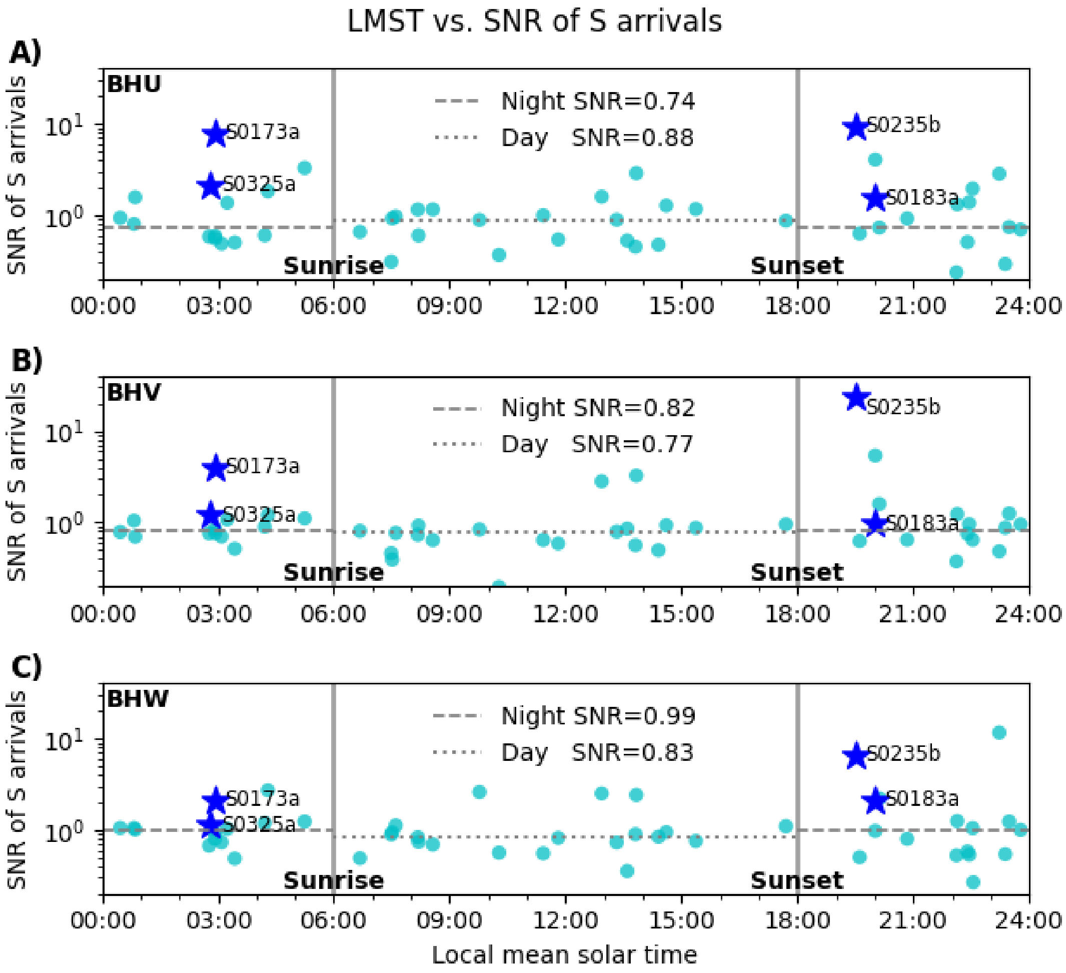

Supplementary Figure 77. The signal-to-noise ratio (SNR) of S-wave arrivals as a function of local mean solar time (LMST) on three seismograph components: A) BHU, B) BHV, and C) BHW. Note the log scale on the vertical axis. Blue stars show the template events, and cyan dots indicate the newly detected MF events. The thick gray lines show the approximate sunrise and sunset times. The dotted and dashed lines mark the median SNR of S arrivals during the day and night, respectively.

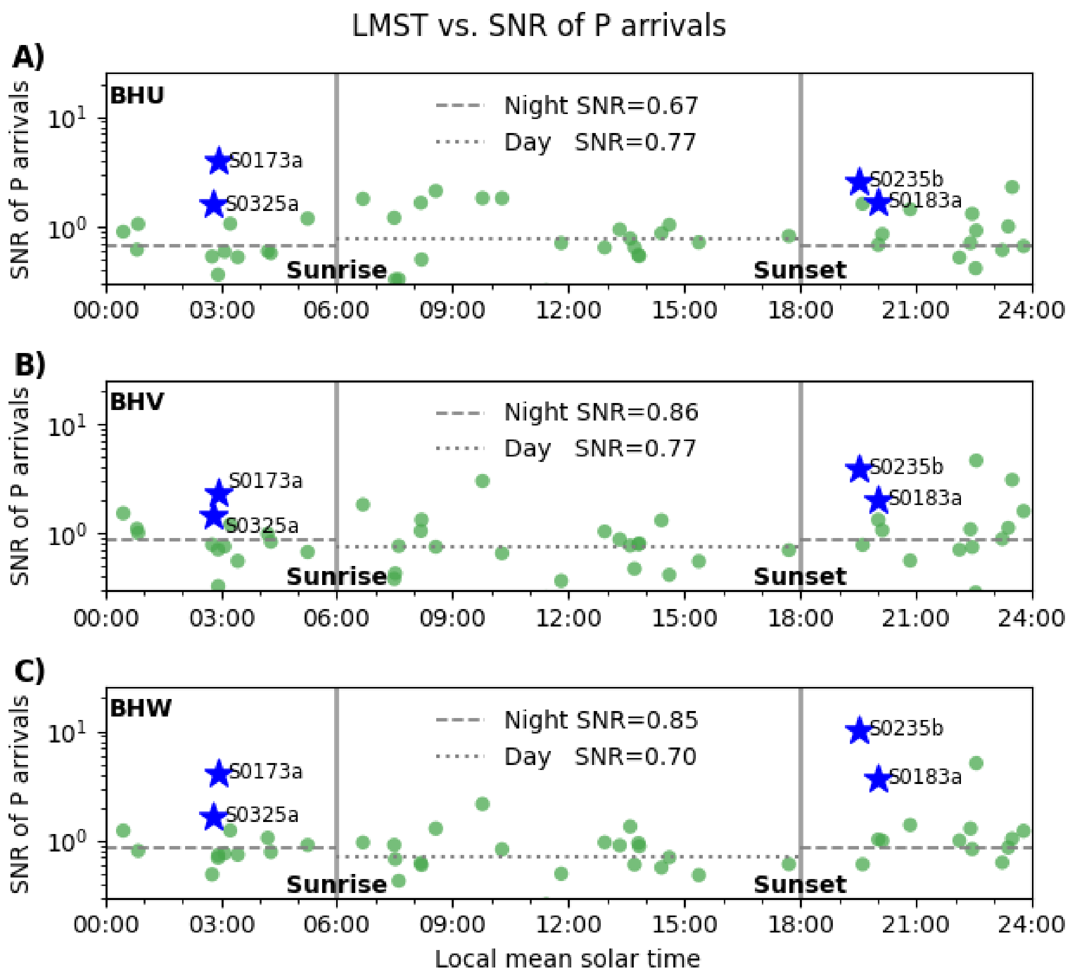

Supplementary Figure 78. The signal-to-noise ratio (SNR) of P-wave arrivals as a function of LMST on three seismograph components: A) BHU, B) BHV, and C) BHW. Note the log scale on the vertical axis. Blue stars show the template events, and cyan dots indicate the newly detected MF events. The thick gray lines show the approximate sunrise and sunset times. The dotted and dashed lines mark the median SNR of P arrivals during the day and night, respectively.

The MF detections have the poorer SNR for both S- and P-wave arrivals than the template events. The SNRs of the S-wave arrivals are generally smaller than 4.0 (Supplementary Figure 77). The SNRs of the P-wave arrivals are generally smaller than 3.0 (Supplementary Figure 78).

The SNR, however, does not display any particular trend with LMST. The SNR of P- and S-wave arrivals is centered around 1.0, which suggests the MF method works well for small event detections. The SNRs of S-wave arrivals during the day are slightly smaller than during the night in Supplementary Figure 77. The observations of P-wave arrivals on BHW and BHV components are similar to S-wave arrivals (Supplementary Figure 78).

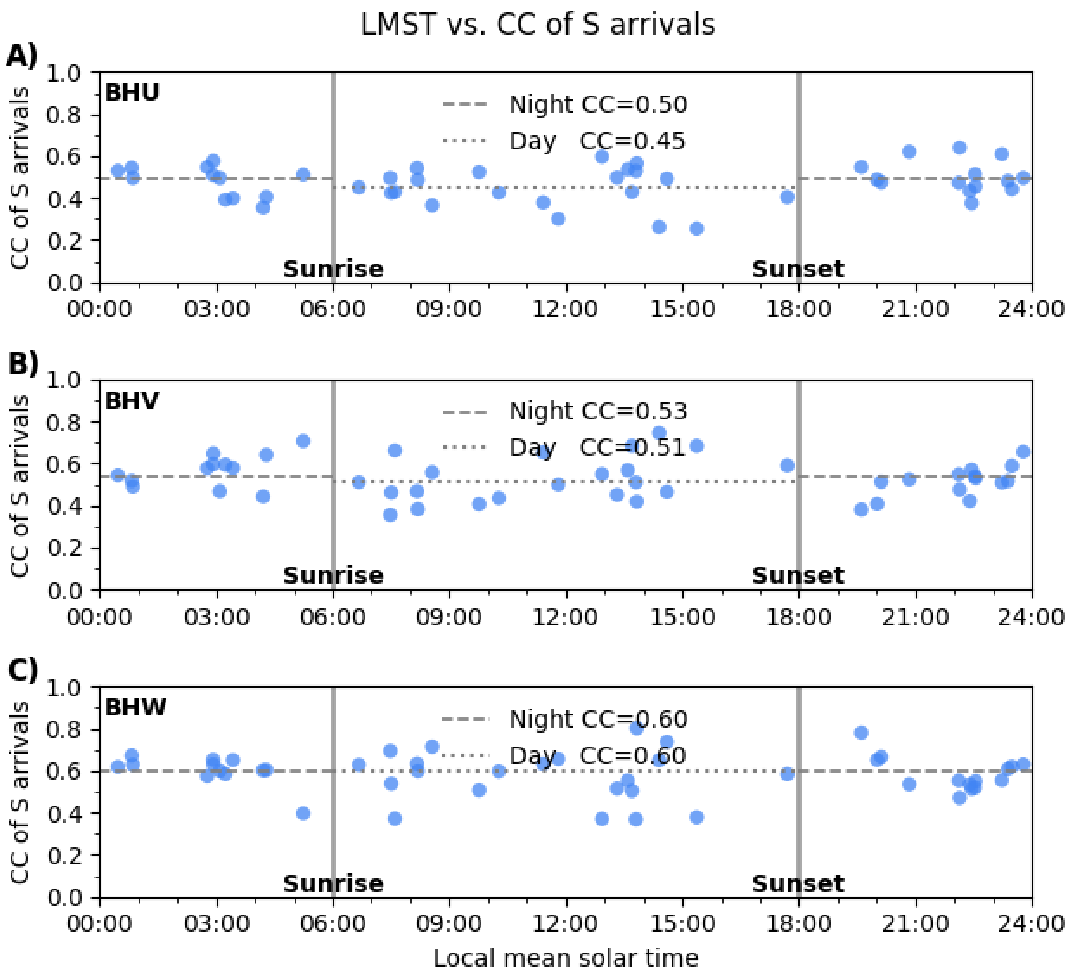

Supplementary Figure 79. The normalized cross-correlation coefficient (CC) of S-wave arrivals as a function of LMST on three seismograph components: A) BHU, B) BHV, C) BHW. The approximate sunrise and sunset times are given in thick gray lines. The dotted and dashed lines mark the median CC values of S arrivals during the day and night, respectively.

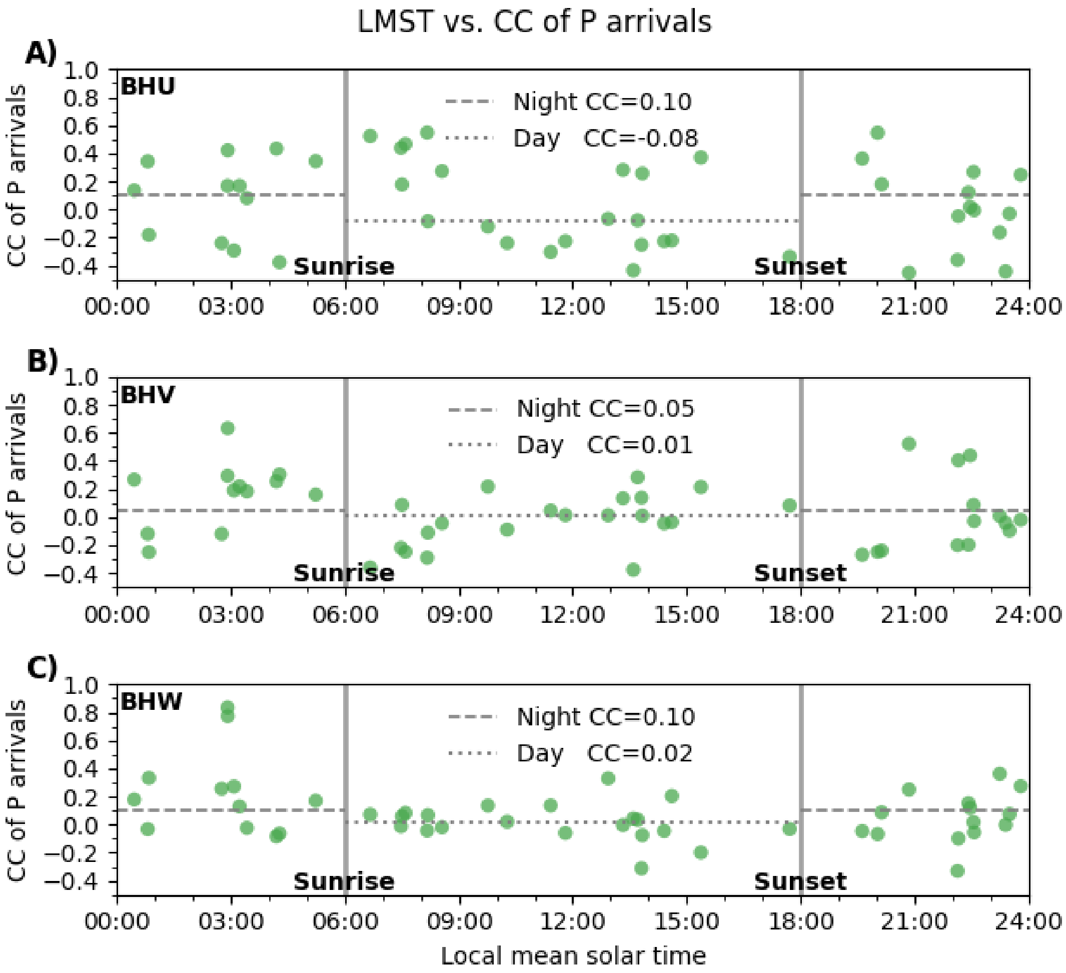

Supplementary Figure 80. The normalized cross-correlation coefficient (CC) of P-wave arrivals as a function of LMST on three seismograph components: A) BHU, B) BHV, C) BHW.

Thick gray lines show the approximate sunrise and sunset times. The dotted and dashed lines mark the median CC values of P arrivals during the day and night, respectively.

Supplementary Figures 79 and 80 are similar to Supplementary Figures 77 and 78, i.e., with no obvious trend for CC as a function of LMST, and most of the small CCs fall between sunrise and sunset, and the CC during the day is slightly smaller than during the night.

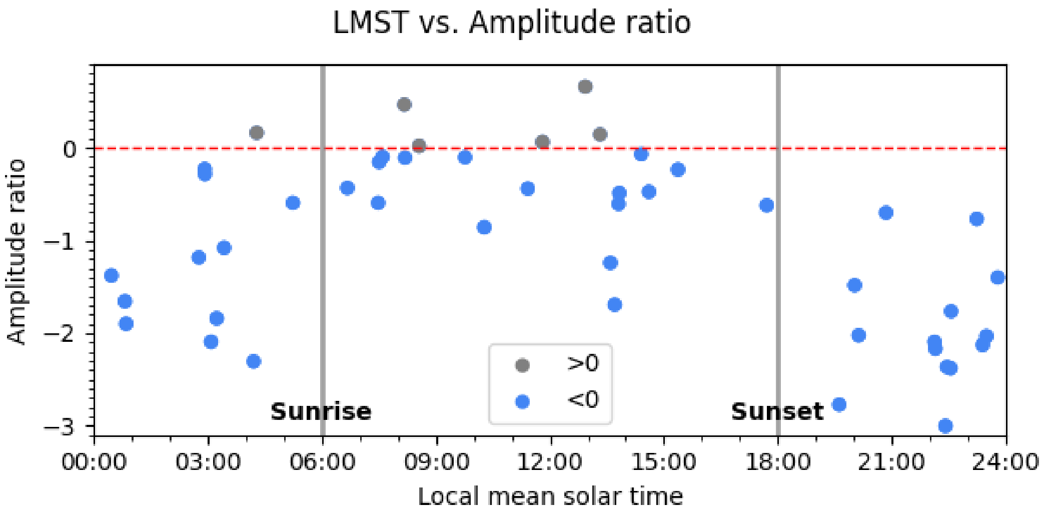

Supplementary Figure 81. The detected vs. template amplitude ratios as a function of LMST. Thick gray lines show the approximate sunrise and sunset times. The amplitude ratios of the newly detected and the template events during the Martian day are above 0 (red dashed line), whereas those at night are below 0. Most amplitude ratios are smaller than the ones for the events S0173a and S0235b (0), shown by the red dashed line.

Supplementary Figure 81 displays the amplitude ratio calculated from Equation (5) as a function of LMST. Most amplitude ratios of the newly detected events are smaller than the template event, i.e., 0 (the red dashed line in Supplementary Figure 81). Thus, the logarithm in

Equation (5) is negative and gives the amplitude ratio of the newly detected events smaller than the amplitude ratio of the template events in most cases.

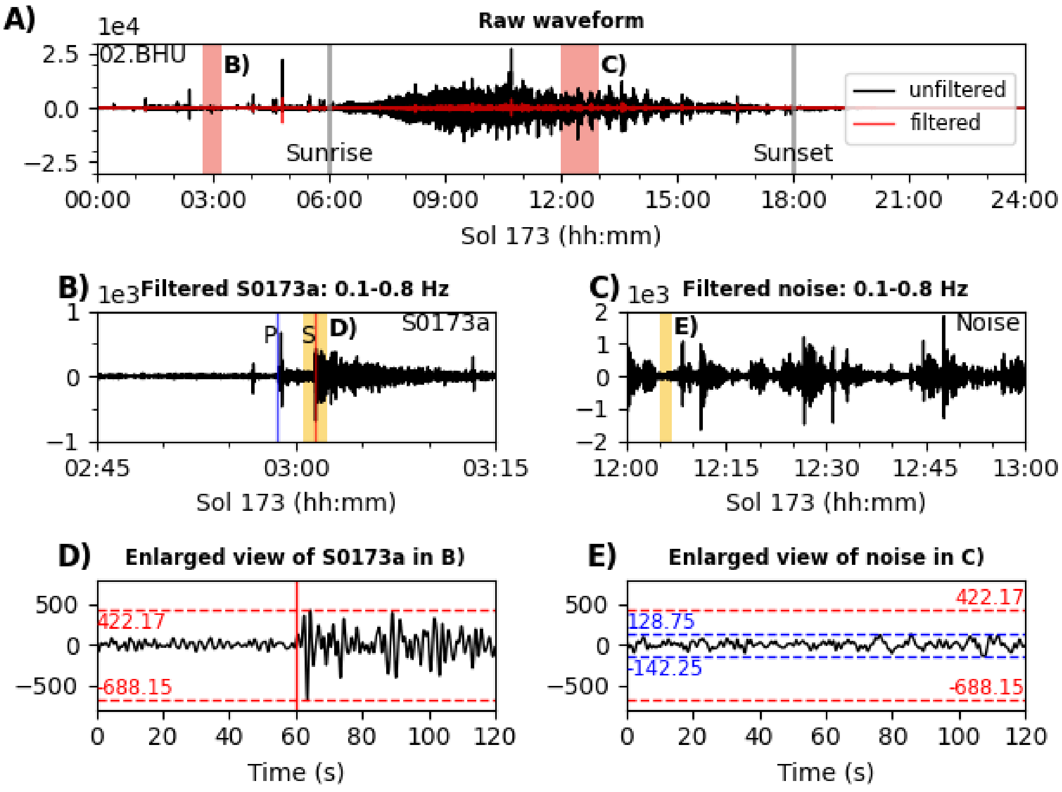

Supplementary Figure 82. Analysis of the absolute amplitudes of 02.BHU waveform for Sol 173: A) Raw waveform of the 02.BHU component for the entire Martian day. The unfiltered and filtered waveforms are in black and red. The two red-shaded areas are enlarged in B) and C). The gray lines show the approximate sunrise and sunset times. B) The filtered waveform of S0173a in the frequency range 0.1-0.8 Hz. Blue and red vertical lines show the P and S arrivals. C) The filtered waveform of noise in the frequency range 0.1-0.8 Hz. D) The enlarged view of the filtered event S0173a, shaded in yellow in B). Horizontal dashed lines show the minimum and maximum amplitudes of S arrival of S0173a. The vertical line marks the S-wave arrival. E) The enlarged view of the filtered noise, shaded in yellow in C). The red lines are the minimum and

maximum amplitude of S0173a from D), and the blue lines are the minimum and maximum amplitudes of the noise.

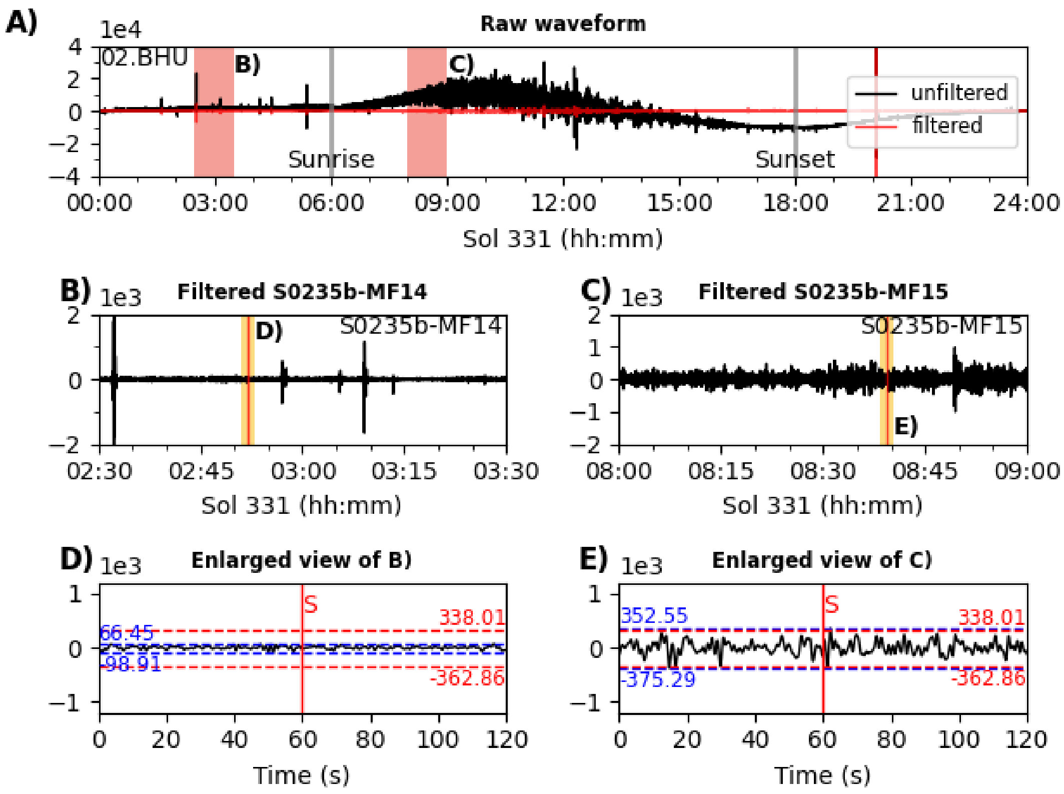

Supplementary Figure 83. Analysis of the absolute amplitudes of 02.BHU waveform for Sol 331 containing S0235b-MF14 and S0235b-MF15: A) Raw waveform of the 02.BHU component for the entire Martian day. The unfiltered and filtered waveforms are in black and red. The two red-shaded areas are shown enlarged in B) and C). The gray lines show the approximate sunrise and sunset times. B) The filtered waveform of S0235b-MF14 in the frequency range 0.1-0.8 Hz. The red vertical line marks the S arrival. C) The filtered waveform of S0235b-MF15 in the frequency range 0.1-0.8 Hz. D) The enlarged view of filtered S0235b-MF14, shaded in yellow in B). The minimum and maximum amplitudes of S arrival of S0235b-MF14 are shown by the blue dashed lines, while the template S0235b minimum and maximum are shown in red dashed lines.

A vertical line notes the S-wave arrival of S0235b-MF14. E) The enlarged view of filtered S0235b-MF15, shaded in yellow in C). The blue shaded lines are the minimum and maximum amplitudes of S0235b-MF15 in D), and the red dashed lines are the minimum and maximum amplitudes of S0235b S-wave arrival. A vertical line notes the S-wave arrival of S0235b-MF15.

To further probe the amplitude ratios, we investigate the waveforms for Sol 173 containing S0173a (Supplementary Figure 82) and Sol 331 containing S0235b-MF14 and S0235b-MF15 (Supplementary Figure 83). Indeed, the amplitudes of the original waveforms (the black lines) are much more prominent during the day (approximately during the time interval 06:00-18:00) than during the night, as seen in Supplementary Figures 82A and 83A. However, strong noises resulted from wind and pressure variation can be significantly suppressed after a two-way bandpass Butterworth filter in the frequency band 0.1-0.8 Hz. The filtered waveforms shown by red lines have much more similar amplitudes for the Martian day and night (Supplementary Figures 82A and 83A). This supports the choice of the frequency band in Supplementary Note 2.

Supplementary Figures 82B and 83D illustrate the enlarged views of S0173a, which gives the minimum and maximum amplitude of S-wave arrival in the range [-688.15, 422.17]. For comparison, we zoom in a noise segment in Supplementary Figures 82C (Sol 173 12:00-13:00) and 82E (Sol 173 12:05-~12:07). The filtered daily noise shown in Supplementary Figure 82E has a much smaller amplitude, namely in the range [-142.25, 128.75], than S0173a during the quiet night. This suggests that 1) the filter can significantly remove the wind and pressure noise, and 2) the wind during the day might not be continuous but intermittent, with significant time intervals as quiet as during the night. Supplementary Figures 82A and 82E also provide insights into the daily SNRs, comparable to but slightly smaller than the nightly SNRs, suggested in Supplementary

Figures 77 and 78. This is further verified by the analysis of Sol 331, including S0235b-MF14 and S0235b-MF15.

We repeat for Sol 331, containing S0235b-MF14 and S0235b-MF15 in Supplementary Figure 83. The strong daily noise is significantly suppressed after the bandpass filter in Supplementary Figure 83A, leading to comparable amplitudes between the day and night. The S-wave amplitude of S0235b-MF14, in the range [-98.91, 66.45], is much smaller than its template S0235b event (Supplementary Figure 83D). This comparison results in the amplitude ratio of -1.185 for S0235b-MF14, much smaller than 0 for S0235b. In addition, the S-wave amplitude of S0235b-MF15 ranges from -375.29 to 352.55, which is comparable to its template S0235b so that, according to Equation (5), the amplitude ratio of S0235b-MF15 (0.018) approaches that of the S0235b, i.e., 0.

We conclude that the SNR and CC have not direct relationships with LMST because the strong wind and pressure noise are effectively suppressed via the two-way bandpass Butterworth filter in the band of 0.1-0.8 Hz. The intermittent wind during the day also contributes to the success of MF detections.

Supplementary Note 4: Traveltime of the SS and SSS phases

We identify two detections of new events (S0173a-MF01, S0173a-MF02), about 24.36 s and 39.56 s after the template of the S0173a event. The two detections bear good cross-correlation coefficients for both P- and S-wave arrivals. In the main text, we interpret these two detections as aftershocks of the main S0173a event.

In addition, it is possible that the two detections could be later arrivals of the S0173a marsquake, like SS and SSS. With the aid of the 1-D Martian models [8], as shown in Supplementary Figure 84, we can predict the traveltime of SS and SSS. We calculate the travel times using these models and assume the focal depth of 60 km, shown in Supplementary Table 4.

As seen, the delays of SS and SSS vary significantly with different models. The observed traveltime differences involving S0173a-MF01, S0173a-MF02, and S0173a have a relatively large bias from the predicted travel times. The Martian structural models may have rather significant uncertainties given the sparseness of the data used to construct them. Given a relatively large number of other newly detected events, we consider the two detections as the aftershocks of S0173a. However, we cannot rule out the possibility of the two detections being the later arrivals of S0173a.

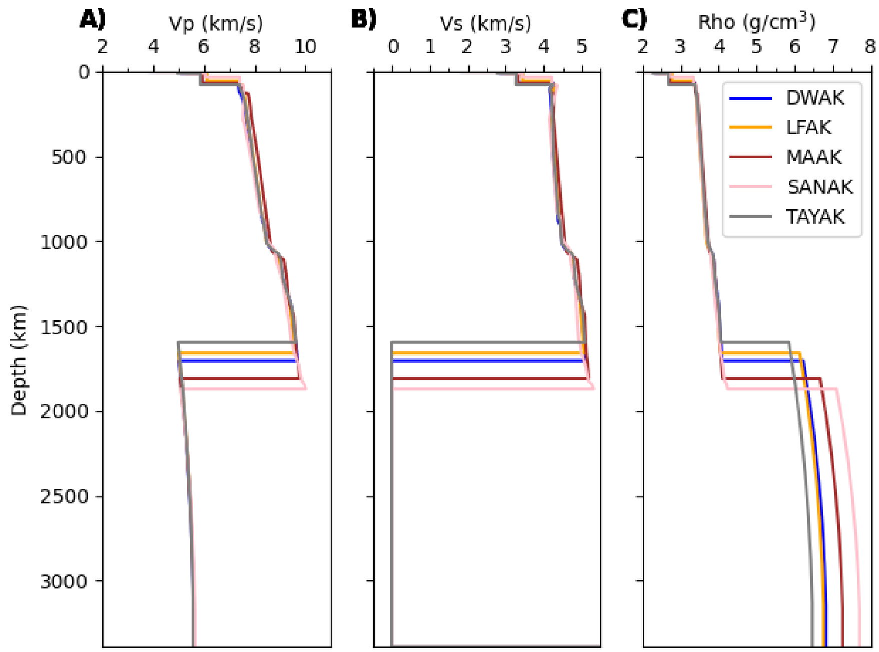

Supplementary Figure 84. The 1-D Martian velocity and density models from [8]: A) P-wave velocity, B) S-wave velocity, and C) density. The model names are labeled in the top right corner.

Supplementary Table 4. Traveltime predicted by the 1-D Martian velocity model in Supplementary Figure 84. MF01_S and MF02_S represent the S arrival of S0173a-MF01 and S0173a-MF02. The SANAK model has a shadow zone for the first S arrival so that no S arrival time is given.

Model	P	S	SS	SSS	S-P	SS-S	SSS-S	MF01_S-S	MF02_S-S
DWAK	232.668	416.546	526.764	470.4	183.878	110.218	53.8536	24.36	39.56
LFAK	228.394	407.078	436.558	457.598	178.684	29.4798	50.5207	24.36	39.56
MAAK	226.86	411.414	447.073	471.305	184.554	35.6593	59.8919	24.36	39.56
SANAK	227.937	-	-	-	-	-	-	24.36	39.56
TAYAK	232.525	417.941	531.89	543.93	185.416	113.949	125.989	24.36	39.56

Supplementary References

1. Lognonne, P. *et al.* SEIS: Insight's Seismic Experiment for Internal Structure of Mars. *Space Sci Rev* **215**, 12, doi:10.1007/s11214-018-0574-6 (2019).
2. Garcia, R. F. *et al.* Pressure Effects on the SEIS‐InSight Instrument, Improvement of Seismic Records, and Characterization of Long Period Atmospheric Waves From Ground Displacements. *Journal of Geophysical Research: Planets* **125**, doi:10.1029/2019je006278 (2020).
3. Giardini, D. *et al.* The seismicity of Mars. *Nature Geoscience* **13**, 205-212, doi:10.1038/s41561-020-0539-8 (2020).
4. Dahmen, N. L. *et al.* Super High Frequency Events: A New Class of Events Recorded by the InSight Seismometers on Mars. *Journal of Geophysical Research: Planets* **126**, e2020JE006599, doi:10.1029/2020je006599 (2021).
5. Sambridge, M., Tkalčić, H. & Jackson, A. Benford's law in the natural sciences. *Geophysical Research Letters* **37**, L22301, doi:10.1029/2010gl044830 (2010).
6. Clinton, J. F. *et al.* The Marsquake catalogue from InSight, sols 0–478. *Physics of the Earth and Planetary Interiors* **310**, 106595, doi:10.1016/j.pepi.2020.106595 (2021).
7. Scholz, J. R. *et al.* Detection, Analysis, and Removal of Glitches From InSight's Seismic Data From Mars. *Earth and Space Science* **7**, doi:10.1029/2020ea001317 (2020).
8. Khan, A. *et al.* Single-station and single-event marsquake location and inversion for structure using synthetic Martian waveforms. *Physics of the Earth and Planetary Interiors* **258**, 28-42, doi:10.1016/j.pepi.2016.05.017 (2016).
