# Supplementary material for: Repetitive marsquakes in Martian upper mantle
Source: Nat Commun. 2022 Mar 30;13:1695. doi: 10.1038/s41467-022-29329-x (PMC8967838; doi:10.1038/s41467-022-29329-x)
Supplement: Supplementary file 4 — Description of Additional Supplementary Files [file 41467_2022_29329_MOESM4_ESM.pdf]

Description of Additional Supplementary files

File name: Supplementary Software

Description: The codes are used to perform the matched-filter method on the broadband waveform recorded by the InSight SEIS.
